# Formulation and biological evaluation of a bacterial nanocellulose-based nanoemulsion hydrogel loaded with *Persea americana* Mill. seed extract: An anti-aging assessment

**DOI:** 10.1371/journal.pone.0359807

**Published:** 2026-11-05

**Authors:** Diah Ramadhani, Tri Suciati, Elfahmi, Ajeng Rana Wulan Sujilah, Heni Rachmawati

**Affiliations:** 1 Department of Pharmaceutics, School of Pharmacy, Bandung Institute of Technology, Bandung, West Java, Indonesia; 2 Faculty of Pharmacy, Universitas 17 Agustus 1945 Jakarta, Jakarta, Indonesia; 3 Research Center for Nanoscience and Nanotechnology, Bandung Institute of Technology, Bandung, West Java, Indonesia; 4 Department of Pharmaceutical Biology, School of Pharmacy, Bandung Institute of Technology, Bandung, West Java, Indonesia; Strides Pharma Science Limited, UNITED STATES MINOR OUTLYING ISLANDS

## Abstract

Avocado (*Persea americana* Mill.) seed is an underutilized by-product rich in catechins and fatty acids, with potential anti-aging properties. However, the utilization of avocado seed in topical formulations is limited by its poor solubility, physicochemical instability, and minimal skin penetration. Therefore, this study aimed to develop and evaluate a bacterial nanocellulose (BNC)-based nanoemulsion hydrogel incorporating avocado seed extract as a topical anti-aging delivery system. Nanoemulsions were prepared using avocado seed oil, Cremophor® RH40, and PEG 400, followed by incorporation into a BNC matrix to obtain a hydrogel-like formulation. Physicochemical characteristics were evaluated by globule size, polydispersity index (PDI), transmittance, pH, viscosity, morphology (TEM), FT-IR profile, and freeze-thaw stability. Skin permeation study was conducted using Franz diffusion cells, while anti-aging activities were assessed using the pinch test, skin hydration, and collagen density assessment in UVB-induced photoaging rat model.The optimized nanoemulsion exhibited a globule size of 20.3 ± 0.10 nm, PDI of 0.109 ± 0.029, transmittance of 99.62 ± 0.27%, and pH of 4.92 ± 0.01 with 1:6:3 ratio of oil, surfactant, and co-surfactant subsequently. The formulation was stable in globule size, PDI, or pH after three freeze–thaw cycles. Incorporation of avocado seed extract up to 5% did not adversely affect nanoemulsion characteristics. The cumulative permeation of BNC-based nanoemulsion was significantly higher than BNC-based conventional emulsion, reaching 198.28 ± 0.54 µg/cm^2^ after 120 min compared with 121.35 ± 0.54 µg/cm^2^. In vivo studies showed that the formulation significantly improved skin elasticity, hydration, and collagen density while reducing UVB-induced wrinkle formation (p < 0.05). In conclusion, the developed BNC-based nanoemulsion hydrogel successfully enhanced the delivery of avocado seed extract and provides a stable and effective platform for topical delivery of natural bioactive compound in anti-aging cosmetic applications.

## Introduction

Avocado (*Persea americana* Mill.) is a fruit with high nutritional value, characterized by a high content of bioactive compounds, vitamins, and fatty acids. However, the utilization of avocado is generally limited to its pulp, while the seeds are discarded as household and food-industry waste.Avocado seeds are rich in bioactive compounds, including phenolic compounds, flavonoids, vitamin C, vitamin E, and approximately 15% of fatty acids [1].In addition, avocado seeds have been identified to the presence of phytochemicals such as catechin and epicatechin, 3-*O*-caffeoylquinic acid (a chlorogenic acid isomer), and glutathione which reported to exhibit potent antioxidant activity by scavenging DPPH radicals, inhibiting lipid oxidation, and neutralizing hydrogen peroxide [2–4].Click or tap here to enter text.These properties of avocado seed extract represent as potential natural ingredients for topical anti-aging formulations by protecting collagen and elastin-forming proteins from oxidative stress [5].However, the topical application of avocado seed extract may be limited by inadequate dispersion within a formulation, physicochemical instability, and limited permeation of bioactive constituents across the topical delivery barrier.

Photoaging, or premature skin aging is induced by ultraviolet (UV) radiation, which promotes oxidative stress and stimulates the production of matrix metalloproteinases (MMPs), leading to the degradation of collagen fibers in the dermal layer [6]. This condition is particularly prevalent in tropical countries such as Indonesia, where high levels of solar radiation lead to frequent UV exposure, highlighting the importance of protecting skin against oxidative damage with effective topical anti-aging formulations. Although numerous cosmetic products claim anti-aging effects, effective delivery of active antioxidant compounds to deeper layers of the skin remains a major challenge [7].

Nanoemulsions are particularly suitable for cosmetic applications as their nanoscale droplet size, high stability, low viscosity, and transparent appearance can support the development of aesthetically acceptable topical formulations. Their homogeneous droplet dispersion may improve the solubilization and distribution of lipophilic or poorly water-dispersible constituents. Their large surface area facilitates efficient delivery of active compounds to penetrate the stratum corneum effectively, thereby enhancing the bioavailability of active ingredients [8]. Oil-in-water (o/w) nanoemulsion systems, are advantageous for incorporating lipophilic active compounds while maintaining a non-greasy texture suitable for facial application and exhibit superior absorption and deeper skin penetration [9].However, inherently low viscosity nanoemulsion may limit their residence time on the skin. Therefore, an appropriate gelling agent should be incorporated to obtain a semisolid dosage form with suitable viscosity and spreadability, thereby maintaining efficient delivery of the nanoemulsion.

One of the most critical factors in semisolid dosage form is the selection of an appropriate gelling agent material, which determines the consistency, stability, spreadability, and overall performance. Gelling agents may be obtained from natural, synthetic, or semisynthetic sources [10].Gelling agents such as hydroxypropyl methylcellulose (HPMC) and sodium carboxymethyl cellulose (CMC-Na) are widely used; however, they mainly function as structural components and exhibit limited bioactivity [11–15]. An alternative gelling agent is the use of bacterial nanocellulose (BNC) [16] which is biopolymer characterized by high biocompatibility, excellent water-holding capacity, superior film-forming ability, and a unique three-dimensional nanofibrillar network [17,18]. These properties enable BNC not only to function as a gelling agent but also to serve as a hydrated nanofibrillar matrix that enhances skin hydration, prolongs residence time on the skin, and supports the modulated releaseof incorporated nanoemulsion droplets [19].

In the present study, avocado seed oil was used as the oil phase, while Cremophor(R) RH40 and PEG 400 were selected as the surfactant and co-surfactant, respectively, to reduce interfacial tension and promote homogeneous nanoemulsion. The nanoemulsion composition was developed by varying the ratio of avocado seed oil, Cremophor^(R)^ RH40, and PEG 400., followed by extract loading screening and incorporation into a BNC-based hydrogel matrix. The predefined critical quality attributes (CQA) of the formulation are nanoscale globule size, narrow size distribution (PDI), skin-compatible pH, high transmittance, suitable viscosity, and good physical stability. The controlled process conditions included the order of ingredient addition, mixing speed, and duration, sonication time, oil phase to water ratio, final homogenization, and incorporation ratio into the BNC matrix.The optimized formulation evaluated for its physicochemical properties, in-vitro skin permeation, and in vivo evaluation against UVB-induced photoaging.

Despite the reported antioxidant potential of avocado seeds, studies on the development of a BNC-based nanoemulsion hydrogel containing avocado seed extract as topical formulation remain limited. Moreover, there is a scarcity of research integrating avocado seed nanoemulsions with BNC-based hydrogel for anti-aging applications, particularly with respect to in vivo evaluation against UVB-induced photoaging. The nanoemulsion was intended to improve solubilization, dispersion, and in vitro permeation, whereas BNC was incorporated as a hydrated topical matrix to provide hydrogel-like consistency and serum-like application properties. Thus, the developed product is more appropriately described as a BNC-based nanoemulsion hydrogel with serum-like topical properties rather than as a conventional liquid serum.

Therefore, we hypothesized that incorporation of avocado seed extract nanoemulsion into a BNC-based hydrogel would provide complementary functions for topical delivery of avocado seed constituents. This study aimed to develop and characterize a BNC-based nanoemulsion hydrogel containing avocado seed extract and oil and to evaluate its in vitro permeation and anti-aging activity in a UVB-induced photoaging rat model.

## Materials and methods

### Preparation of avocado seed extracts

Avocado fruits were obtained from local farmers in Lembang, West Bandung Regency, West Java, Indonesia. The plant material was identified at the National Research and Innovation Agency, Indonesia (identification number: B-1821/II.6.2/DI.05.07/6/2022). The fruits were cut in half, and the seeds were separated and cleaned. The seeds were dried in a drying oven at 60 °C for at least five days and subsequently ground into a powder using a grinder. A total of 500 g of avocado seed powder was extracted by maceration using ethanol with ratio of 1:10 (w/v) for three cycles (24 h/cycle) with intermittent manual agitation. For each maceration cycle, 5 L of ethanol was used. The combined filtrates were concentrated using a rotary evaporator (230 mbar; 10 °C chiller; 50 °C heating bath) [20,21].

### Metabolite profiling using UPLC-MS/MS

The avocado seed extract was dissolved in methanol and analyzed using UPLC-MS/MS (ACQUITY UPLC I-Class System coupled with a Xevo G2-XS Q-Tof Mass Spectrometer, Waters, USA). Chromatographic separation was achieved using gradient elution with water and acetonitrile, both containing 0.1% formic acid. Mass spectral data were acquired and processed using MassLynx software (version 4.1). Metabolites were tentatively identified by comparing their m/z values and spectral data with those available in the ChemSpider and PubChem databases [22,23].

### Preparation of nanoemulsion

The nanoemulsion containing avocado seed extract was prepared by initially forming an oil phase consisting of avocado seed oil (ASO), Cremophor® RH40 (BASF, Ludwigshafen, Germany) as the surfactant, and PEG 400 (Brataco, Jakarta, Indonesia) as the co-surfactant. The nanoemulsion formulations were developed by varying the proportions of avocado seed oil (ASO), Cremophor® RH40, and PEG 400 presented in Table 1.

**Table 1:** Formula of nanoemulsion.

Formula	ASO (ratio)	Cremophor® RH40 (ratio)	PEG 400 (ratio)
F1	1	8	1
F2	1	7	2
F3	1	6	3
F4	2	7	1
F5	3	6	1

Various concentrations of avocado seed extract, ranging from 0.1% to 5.0%, were incorporated into the optimum nanoemulsion oil phase formula. The extract, oil, surfactant, and co-surfactant were mixed using a magnetic stirrer at 500 rpm for 2 h. Subsequently, the mixture was sonicated for 1 h using a bath sonicator [24]. Deionized water was then added to the oil phase at a 1:3 ratio (oil phase to water), and the mixture was stirred continuously until a clear and homogenous nanoemulsion was obtained. The order of ingredient addition, mixing speed and duration, sonication time, and oil-phase-to-water ratio were controlled during nanoemulsion preparation. The resulting nanoemulsion was characterized for visual appearance globule size, polydispersity index, transmittance, and pH. The optimum nanoemulsion formulation was selected from the tested formulations based on predefined criteria, including clear visual appearance, globule size below 100 nm, PDI below 0.3, high transmittance, and a pH suitable for topical application.

### Physicochemical properties of nanoemulsion

The physical characteristics of the nanoemulsion, including particle size, polydispersity index (PDI), percent transmittance, viscosity, and morphology, were systematically evaluated.

Particle size and PDI were determined using a particle size analyzer (Delsa™ Nano, Beckman Coulter, Brea, CA) at 25 °C, following a previously reported method [25]. The optical clarity of the nanoemulsion was assessed by measuring the percent transmittance (%T) at 650 nm using a UV–Visible spectrophotometer, with distilled water as the blank. The nanoemulsion’s viscosity was measured using a Brookfield viscometer at 25 °C with a shear rate of 6 rpm. Morphological characterization of the nanoemulsion droplets was conducted using a transmission electron microscope (JEOL JEM1400, Tokyo, Japan) according to a previously established procedure, with observations performed at magnifications of 30,000× and 120,000× [26].

### FT-IR characterization of nanoemulsion

Fourier transform infrared (FT-IR) spectroscopy was done at room temperature using Shimadzu IR (Shimadzu, UK). FT-IR analysis was carried out for pure avocado seed extract, pure avocado seed oil, pure Cremophor® RH40, pure PEG 400, nanoemulsion without extract, and nanoemulsion-avocado seed extract. The spectral range was 400−4000 cm^-1^ [27].

### Stability of nanoemulsion

The stability of the nanoemulsion was evaluated using a freeze–thaw method over three cycles: storage at −20 °C for 48 h, followed by storage at 25 °C for 48 h. The parameters observed included phase separation, particle size, polydispersity index, and pH value [28].

### Preparation of Bacterial nanocellulose

Bacterial nanocellulose (BNC) was produced using G*luconacetobacter xylinus* InaCC B407 obtained from the National Research and Innovation Agency, Indonesia. The starter culture was prepared in Hestrin-Schramm medium containing glucose (2.0%, w/v), peptone (0.5%, w/v), yeast extract (0.5%, w/v), disodium hydrogen phosphate (0.27%, w/v), and citric acid (0.115%, w/v). All components were dissolved in warm distilled water and sterilized by autoclaving at 121 °C for 15 min. After cooling to room temperature under aseptic conditions, two inoculation loops of *G. xylinus* stock culture were transferred into the sterile medium. The starter culture was incubated at 30 °C [29] for 20 hours in a shaker [30].

For BNC production, a nira-based fermentation medium was prepared using nira as the main carbon source, supplemented with disodium hydrogen phosphate (0.27%, w/v) and citric acid (0.115%, w/v). The medium was sterilized by autoclaving at 121 °C for 15 min and allowed to cool to room temperature under aseptic conditions. The activated *G. xylinus* starter culture was inoculated into the sterile nira-based medium at 10% (v/v) of the total fermentation volume. Fermentation was conducted under static culture conditions at 30^o^C for 9 days.

The resulting BNC pellicles were harvested and washed with running water to remove residual medium and adhering impurities. Purification was performed by soaking the BNC in 1 M NaOH at 80^o^C for 1 h, followed by bleaching in 1% NaOCl for 1 h. The purified BNC was washed repeatedly with water to remove residual alkali and bleaching agent, the stored at 4^o^C until formulation use.

The purified BNC was characterized by FTIR spectroscopy and pH measurement. Bacterial nanocellulose yield was calculated by dividing the mass of purified BNC by the fermentation-medium volume and was expressed as g/L.

### Preparation of BNC + NE-Ehydrogel

For preparation of the BNC-based nanoemulsion hydrogel, BNC was incorporated into the nanoemulsion after the other formulation components had been dissolved. The mixture was stirred at 300 rpm and 75°C for 15 min using a magnetic stirrer, followed by mechanical homogenization at 700 rpm for 15 min [31]. Formulation was evaluated based on homogeneity, absence of phase separation, viscosity, spreadability, physical stability, pH, and in vitro permeation performance.

### In-vitro skin permeation study

The diffusion study was conducted using Franz diffusion cells. The receptor compartment was filled with phosphate-buffered saline (PBS, pH 7.4) to a total volume of 15 mL. Approximately 500 mg of the sample formulation was uniformly applied onto a cellophane membrane. The temperature was maintained at 37 ± 1 °C, and the stirring speed was set at 120 rpm. Aliquots were withdrawn from the receptor compartment at predetermined time intervals of 5, 10, 15, 30, 45, 60, 90, and 120 min. At each sampling point, 3 mL of the receptor medium was withdrawn and immediately replaced with an equal volume of fresh PBS to maintain sink conditions. The samples were analyzed using a UV–Visible spectrophotometer at 450 nm. The cumulative amount of flavonoids permeated per unit area was calculated using the regression equation derived from the calibration curve [28,32].

### In-vivo evaluation of anti-aging effect

#### Ethics statement.

All animal procedures were reviewed and approved by the Animal Research Ethics Committee, Institut Teknologi Bandung (KEPHP-ITB), Indonesia (approval number: KEP/I/2024/III/H310124DR/BANA). All procedures involving animals were conducted in accordance with the approved research protocol.

#### Animals.

Male Wistar rats (*Rattus norvegicus*), aged 42–45 days and weighing 190 ± 10 g, were obtained from CV. Kencana Animal Breeding and Riset Toksikologi, Indonesia. The rats were acclimatized for 7 days before the experiment to allow adaptation to the laboratory environment. The rats were housed in plastic cages with wire partitions and bedding made of rice husk. Each cage contained four rats. All animals were provided with ad libitum access to food and water under standard environmental conditions, including controlled room temperature and a 12 h light/12 h dark cycle [33]. The animals were randomly divided into six groups: normal control, model control, positive control, and three treatment groups receiving the test formulations [34]. The minimum sample size was determined using Federer’s formula [35]. Based on this calculation, each group consisted of four rats.

#### UVB-Induced photoaging model in rats.

In this study, the dorsal surface of the rat skin was shaved with a razor over an area of 5 × 5 cm^2^, leaving the area hairless during the experimental period. The frequency of exposure was determined three times a week for four weeks. Radiation exposure was performed using a Philips NB UVB lamp (model PL-S 9W/01-2P) with a wavelength of 305–311 nm. The exposure doses were 50 mJ/cm^2^ in the first and second weeks, 70 mJ/cm^2^ in the third week, and 80 mJ/cm^2^ in the fourth week, for a total of 840 mJ/cm^2^. At the end of the experimental period, rats were anesthetized using diethyl ether in an anesthesia chamber before dorsal skin photography and photoaging assessment. Adequate anesthesia was confirmed by the absence of response to toe-pinch stimulus. Immediately after the photoaging assessment, the Rats were sacrificed using cervical dislocation and death was confirmed before dorsal skin tissue collection for histological analysis. Wrinkle formation was evaluated and quantified using ImageJ 1.53e [36].

#### Photoaging evaluation of dorsal skin.

At the end of the experimental period, the UV-induced dorsal skin of each rat, which was under anesthesia, was photographed; the grade of photodamage was determined by referring to the grading scale described in Table 2. The skin photoaging level ranged from 0 (normal skin) to 6 (severely damaged skin).

**Table 2:** Grading scale for evaluation of photoaging.

Grade	Evaluation Criteria
0	Smoothness without any wrinkles; fine striations running the length of the body
1	Fine striations
2	A few shallow wrinkles; disappearance of all fine striations
3	Shallow wrinkles across the dorsal skin
4	Deep and coarse wrinkles with laxity
5	Increased deep wrinkles
6	Surface accompanied by severe wrinkles; development of lesions

#### Evaluation of skin elasticity using the pinch test.

A pinch test was performed on a rat using the method of Hou [34]. Briefly, after the end of the experimental period, the midline of the dorsal skin of the anesthetized rat was grasped with the fingers as much as possible (without lifting the animal into the air), the pinch was released, and the skin recovery time was immediately calculated.

#### Skin hydration measurement.

At the end of the experimental period, the rats were sacrificed by cervical dislocation. The exposed skin on the back of the depilated rats was removed, about 0.2 g. The fatty tissue in the dermis was removed, and the wet weight of the skin was measured. Place the skin in an oven and dry to constant weight at 70° C, then weigh the dry skin. The skin hydration was calculated using the following equation: [34]


$$\begin{array}{c} Skin\;hydration\;(\%)=\frac{M\;(wet\;weight-dry\;weight)}{M\;(wet\;weight)}\;\times \text{1}\text{0}\text{0}\% \end{array}$$

#### Analysis of collagen density.

Observation was conducted microscopically on Masson’s trichrome-stained slides using an Olympus® viewer at 12x20 magnification. Collagen density was measured in the dermal layer of the skin. Measurement of collagen area was done through software (ImageJ®) and displayed as a percentage. Measurements were taken in 4 fields of view, and the average value was calculated [34].

### Statistical analysis

All data are presented as mean ± standard deviation (SD). ANOVA and Tukey’s post hoc multiple comparison using Minitab were used for statistical analysis, and a p-value of <0,05 was considered statistically significant.

## Results

### Avocado seed extraction yield

The maceration of 500 g of dried avocado seed powder with ethanol produced a viscous extract with an extraction yield of 13.44% (w/w).

### Metabolite profiling using UPLC-MS/MS

The metabolite profile of the avocado seed extract was characterized based on chromatographic and mass-spectral data obtained during analysis. Several compounds were tentatively identified based on their retention times, molecular masses, and predicted molecular formulas (Table 3).

**Table 3:** Compounds identified in avocado seed extract by LC–ESI–MS analysis.

Peak	Retention Time (minute)	Mass	Formula	Compound
1	3.81	578.520	C_30_H_26_O_12_	Procyanidin
2	5.72	222.194	C_11_H_10_O_5_	Isofraxidin
3	6.66	205.210	C_11_H_11_NO_3_	5-Methoxyindoleacetate
4	9.39	266.419	C_17_H_30_O_2_	Heptadecadienoic acid
5	11.25	303.438	C_16_H_33_NO_4_	dodecanoic acid (2-hydroxy-1,1-Bis-hydroxymethyl-ethyl) amide
6	11.32	266.419	C_17_H_30_O_2_	Heptadecadienoic acid
7	17.45	954.200	C_47_H_83_N_7_O_13_	18-[2-[2-[2-[2-[2-[2-[[(5S)-5-Carboxy-5-[[3-[3-(1H-imidazol-5- yl)propanoylamino]-3-methyl-2-oxobutyl]amino]pentyl]amino]-2- oxoethoxy]ethoxy]ethylamino]-2-oxoethoxy]ethoxy]ethylamino]-18- oxooctadecanoic acid

### Formulation and characterization of nanoemulsion

To provide a clear overview of the formulation strategy, the predefined critical quality attributes (CQAs), critical material attributes (CMAs), and critical process parameters (CPPs) are summarized in the Supplementary Information (S1 Table). These factors served as the basis for optimizing the nanoemulsion formulation described in the following sections.

The characteristics of various nanoemulsion formulations are presented in Table 4 and Fig 1.

**Table 4:** Characteristics of nanoemulsions with varying oil–surfactant–co-surfactant compositions.

Formula	Visual appearance	Globule size (nm)	PI	%Transmittance	pH
FI	Clear	259.4 ± 3.26	0.584 ± 0.069	99.62 ± 0.13	4.67 ± 0.01
FII	Clear	22.8 ± 0.40	0.224 ± 0.017	99.62 ± 0.27	4.81 ± 0.00
FIII	Clear	20.3 ± 0.10	0.109 ± 0.029	99.62 ± 0.27	4.92 ± 0.01
FIV	Cloudy	3524.9	0.813	75.30 ± 2.21	4.40 ± 0.02
FV	Cloudy	3301.7	0.703	9.36 ± 5.76	4.20 ± 0.02

**Fig 1:**
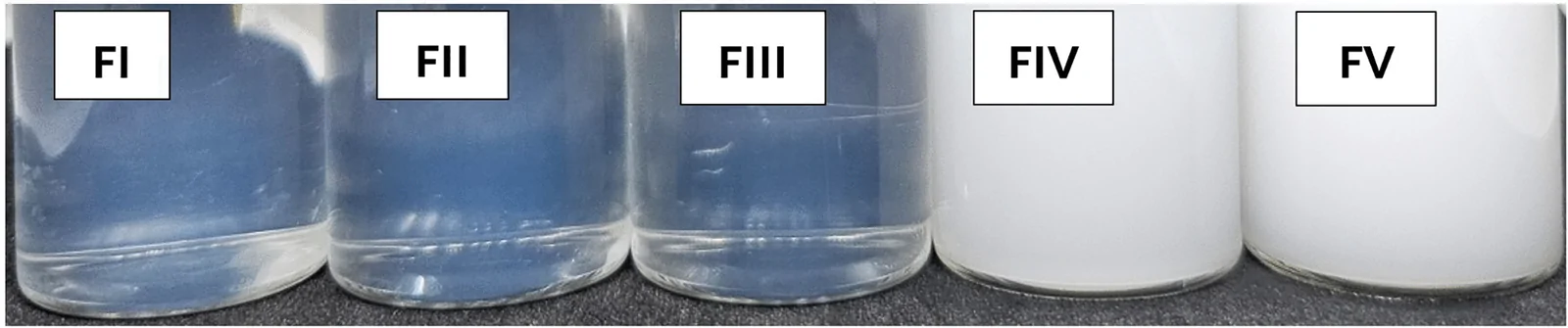
Visual appearance of nanoemulsion formula FI to FV (left to right).

Based on the data presented in Table 4, clear nanoemulsions (NEs) were obtained upon water addition in formulations FI–FIII, as evidenced by their clear and transparent appearance. In contrast, formulations FIV and FV failed to form nanoemulsions, as indicated by their cloudy visual appearance. All clear formulations (FI–FIII) demonstrated high transmittance values of approximately 99.6%, while cloudy formulations FIV and FV showed substantially lower transmittance. Among the clear formulations, FIII showed the most favorable physicochemical characteristics, with a mean globule size of 20.3 ± 0.10 nm, a polydispersity index (PDI) of 0.109 ± 0.029, a transmittance value of 99.62 ± 0.27%, and pH values of 4.92. Therefore, FIII was selected as the nanoemulsion precursor for subsequent avocado seed extract loading and incorporation into the BNC-based nanoemulsion hydrogel.

Optimization of avocado seed extract loading in the nanoemulsion system was conducted using FIII as the optimized oil nanoemulsion formulation. The concentration of extract incorporated and the loading capacity were evaluated based on several parameters summarized in Table 5 and Fig 2.

**Table 5:** Characteristics of avocado seed extract nanoemulsion.

Formula	Avocado seed extract in 10 g oil phase (mg)	Visual appearance	Globule size (nm)	Polydispersity Index	%Transmittance	pH
NE 0	–	Clear	20.3 ± 0.10	0.109 ± 0.029	99.62 ± 0.27	4.92 ± 0.01
NE I	10	Clear	20.7 ± 0.10	0.019 ± 0.017	99.77 ± 0.00	4.93 ± 0.04
NE II	100	Clear	20.2 ± 0.65	0.051 ± 0.062	99.77 ± 0.00	4.94 ± 0.03
NE III	250	Clear	22.4 ± 0.31	0.095 ± 0.080	98.93 ± 0.26	4.87 ± 0.01
NE IV	500	Clear	23.7 ± 1.01	0.196 ± 0.045	98.55 ± 0.47	4.79 ± 0.01

**Fig 2:**
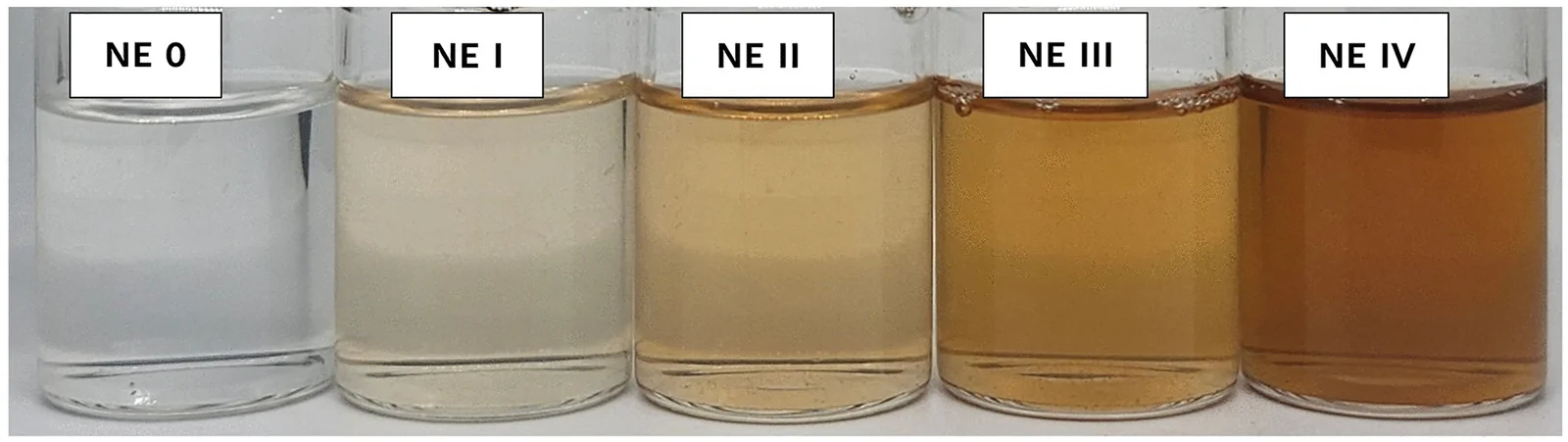
Visual appearance of avocado seed extract nanoemulsion formula NE 0 to NE IV (left to right).

The incorporation of avocado seed extract up to 500 mg did not result in any significant changes in the nanoemulsion's visual appearance, globule size, PDI, transmittance values, or pH. Increasing the concentration of avocado seed extract led to a gradual colour change in the formulation from colourless to dark brown, as shown in Fig 2. Therefore, it can be concluded that the solubility of avocado seed extract was relatively high, reaching up to 5%, and was evenly distributed throughout the external phase of the NE system without compromising the formulation's clarity. The polydispersity index (PDI) values of all formulations were less than 0.3, indicating that the formed globules were relatively uniform in size.

### Morphology of nanoemulsion

The morphology of the nanoemulsion was characterized using TEM, as shown in Fig 3. The globules of the avocado seed extract nanoemulsion appeared spherical with a homogeneous size distribution. These results indicate that the nanoemulsion remained stable and did not aggregate.

**Fig 3:**
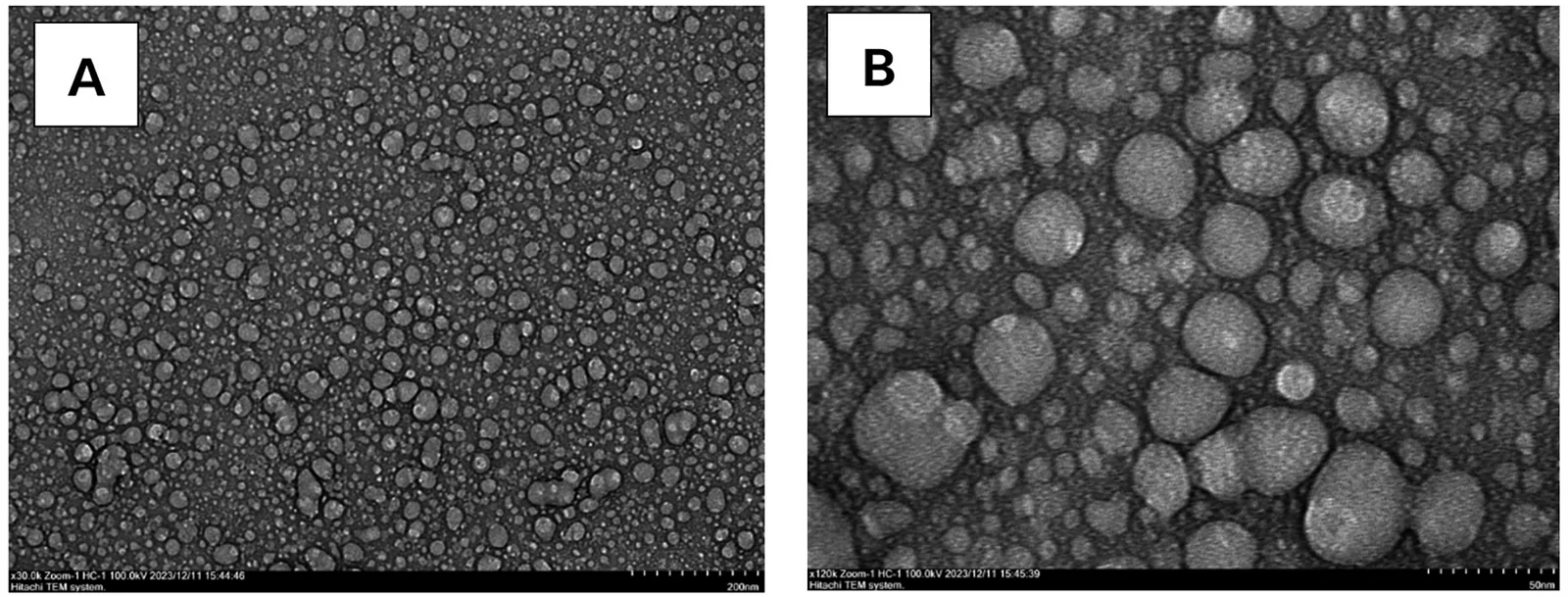
Morphology of extract avocado seed nanoemulsion (NE EAS) observed using TEM. (a) NE EAS at 30.0k× magnification with a scale bar of 200 nm; (b) NE EAS at 120k× magnification with a scale bar of 50 nm.

### FT-IR characterization of nanoemulsion

Analysis of FT-IR confirmed the successful incorporation of avocado seed oil (ASO), Cremophor RH-40 and PEG-400 into the nanoemulsion system, as it reflected a combination of their functional groups (Fig 4). Avocado seed oil (ASO) showed a prominent ester carbonyl band at approximately 1740 cm^-1^ and aliphatic C-H stretching vibrations at 2920 and 2850 cm^-1^, confirming its triglyceride structure. Cremophor RH-40 and PEG-400 displayed dominant C-O-C stretching bands around 1100 cm^-1^, characteristic of polyoxyethylene-based surfactants. The FT-IR spectrum of EAS exhibited a broad absorption band around 3400 cm^-1^ attributed to O-H stretching of phenolic compounds, along with characteristic aromatic C = C vibrations at 1600−1630 cm^-1^ and C-O stretching bands in the 1240−1100 cm^-1^ region. The FT-IR spectrum of EAS-loaded nanoemulsion (NE-EAS) retained all major characteristic absorption bands of EAS and ASO without the appearance of significant band shifts.FT-IR analysis of the EAS-loaded nanoemulsion (NE-EAS) retained all major characteristic absorption bands of the extract and oil phase without the appearance of new peaks or significant band shifts, indicating the absence of chemical interactions between EAS and the excipients. These findings suggest that the extract was physically encapsulated within the nanoemulsion matrix, preserving its chemical integrity.

**Fig 4:**
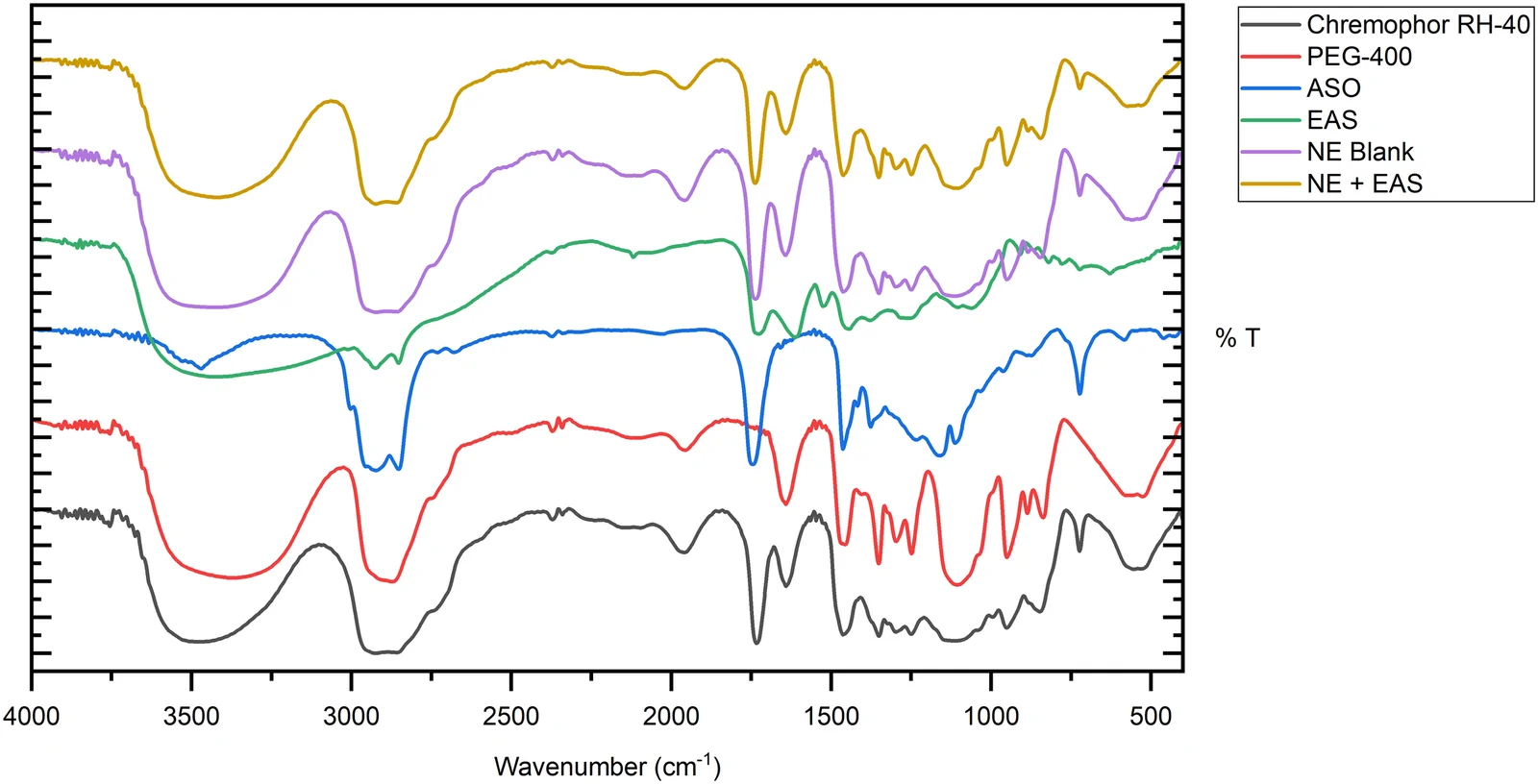
FT-IR characterization of nanoemulsion.

### Stability of nanoemulsion

The physical stability of the nanoemulsion was evaluated using a freeze–thaw method by monitoring changes in globule size, polydispersity index (PDI), and pH (Fig 5). All formulations exhibited relatively stable globule sizes, with a slight increase throughout the freeze–thaw cycles. Among all formulations, FIII demonstrated the most stable in globule size across cycles, indicating superior physical stability compared with FI and FII. The PDI values of all formulations varied within the ranges of 0.193–0.526 for FI, 0.344–0.663 for FII, and 0.326–0.397 for FIII. Consistent with the globule size results, FIII exhibited lower PDI values, reflecting a more homogeneous particle size distribution. The pH values of all formulations remained relatively constant during the freeze–thaw testing, with ranges of 4.50–4.67 for FI, 4.64–4.81 for FII, and 4.78–4.92 for FIII. These results indicate that the pH of the nanoemulsion formulations remained stable and within a skin-compatible range under thermal stress conditions.

**Fig 5:**
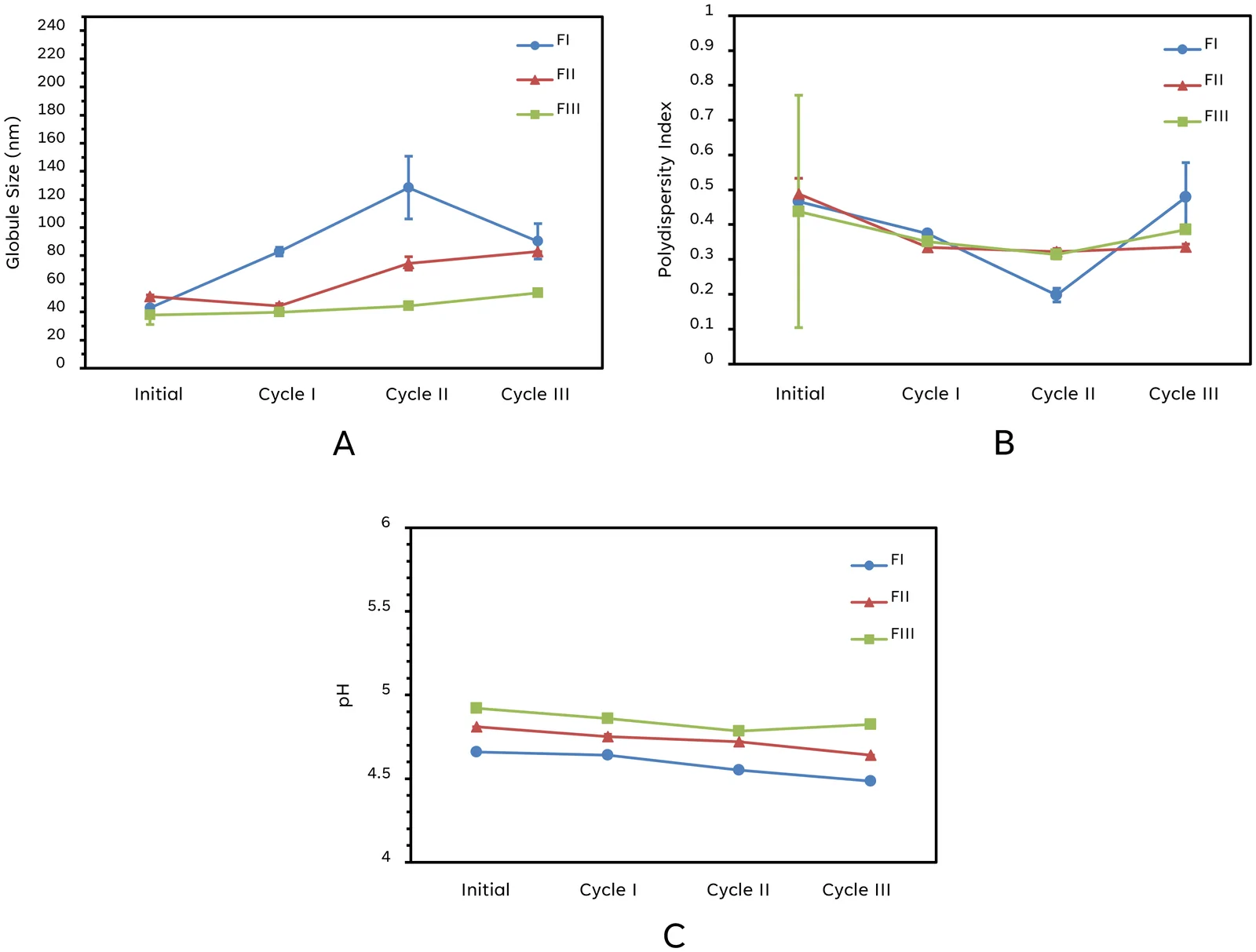
Freeze-thaw stability for 3 cycles. (a) globule size; (b) polydispersity index; and (c) pH.

### BNC yield determination and characterization

After 9 days of static fermentation, the wet purified BNC obtained from 100 mL of fermentation medium was 10.79 ± 0,55 g (n = 3), corresponding to a wet purified BNC yield of 107.93 ± 5.50 g/L.

The FTIR spectrum of purified BNC is shown in Fig 7 (black line). Purified BNC exhibited a broad absorption band at approximately 3330.35 cm^-1^, corresponding to O-H stretching vibrations from the abundant hydroxyl groups in cellulose. A band at approximately 1633.08 cm^-1^ was associated with O-H bending of absorbed water, which is commonly observed in hydrated bacterial nanocellulose. Several characteristic cellulose bands were observed in the fingerprint region. The absorption bands at 1163.08, 1111, and 1058.92 cm^-1^ were attributed to C-O-C and C-O stretching vibrations of the cellulose polysaccharide backbone. The band around 897 cm^-1^ indicated β-1,4-glycosidic linkage, supporting the cellulose structure of the purified BNC. These characteristic peaks confirmed that the purified material showed the typical functional groups of bacterial nanocellulose.

### Formulation and characterization of BNC + NE-E hydrogel

As shown in Table 6 and Fig 6, three hydrogel formulations with different gelling systems were evaluated for physical appearance, spreadability, viscosity, and pH. All formulations (Hydrogel1 – Hydrogel3) exhibited a brown, transparent gel appearance, indicating good homogeneity and absence of phase separation or precipitation.Hydrogel1 showed the highest spreadability (5.300 ± 0.200 cm), indicating a softer gel structure, while Hydrogel1 and Hydrogel3 exhibited lower spreadability values of 4.801 ± 0.505 cm and 4.659 ± 0.140 cm, respectively. Viscosity measurements demonstrated notable differences among formulations. Hydrogel1 showed the lowest viscosity (352.62 mPa·s), followed by Hydrogel2 (388.24 mPa·s), while Hydrogel3 exhibited the highest viscosity (510.81 mPa·s). These results reflect the influence of the gelling agent type on the rheological properties of the formulations. The pH values of all formulations ranged from 5.12 to 5.41. No extreme pH deviations were observed, indicating that all formulations are suitable for topical application.

**Table 6:** Characteristics of NE-E Hydrogel.

Formula	Carbomer 940	HPMC	BNC	Glycerin	Propyleneglycol	Physical Appearance	Spreadability (cm)	Viscosity (mPa.s)	pH
Hydrogel1	–	–	20	–	–	Brown Transparent gel	5.300 ± 0.200	352.62	5.41 ± 0.05
Hydrogel2	–	5	–	5	15	Brown Transparent gel	4.801 ± 0.505	388.24	5.19 ± 0.02
Hydrogel3	1	–	–	5	15	Brown Transparent gel	4.659 ± 0.140	510.81	5.12 ± 0.01

**Fig 6:**
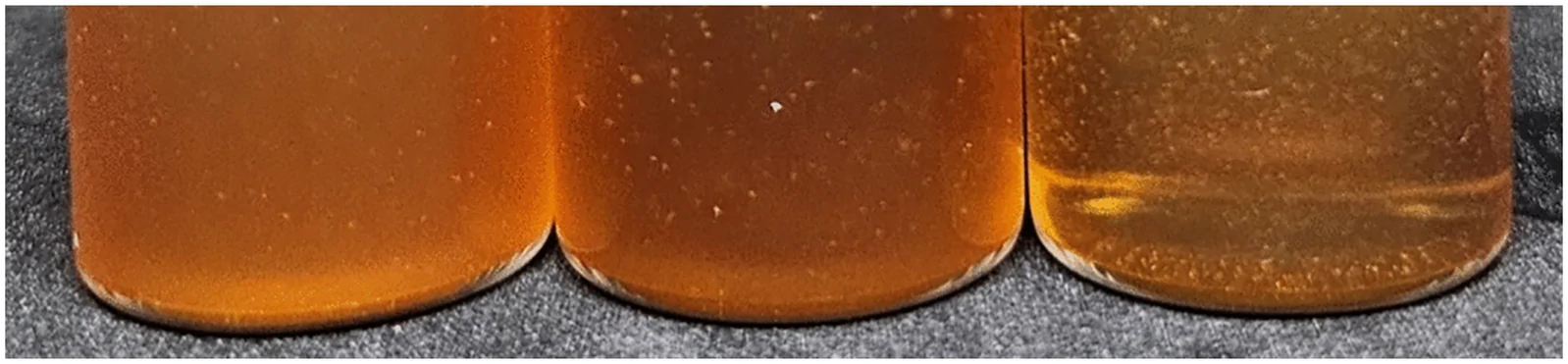
Visual appearance of serum avocado seed extract formula Ser1 to Ser3 (left to right).

### FT-IR characterization

The FT-IR spectra illustrate a comparison of BNC (black line), NE (blue line), and BNC + NE (red line) (Fig 7). The observed shifts in absorption bands indicate potential chemical interactions between the avocado seed extract nanoemulsion and the BNC matrix.

**Fig 7:**
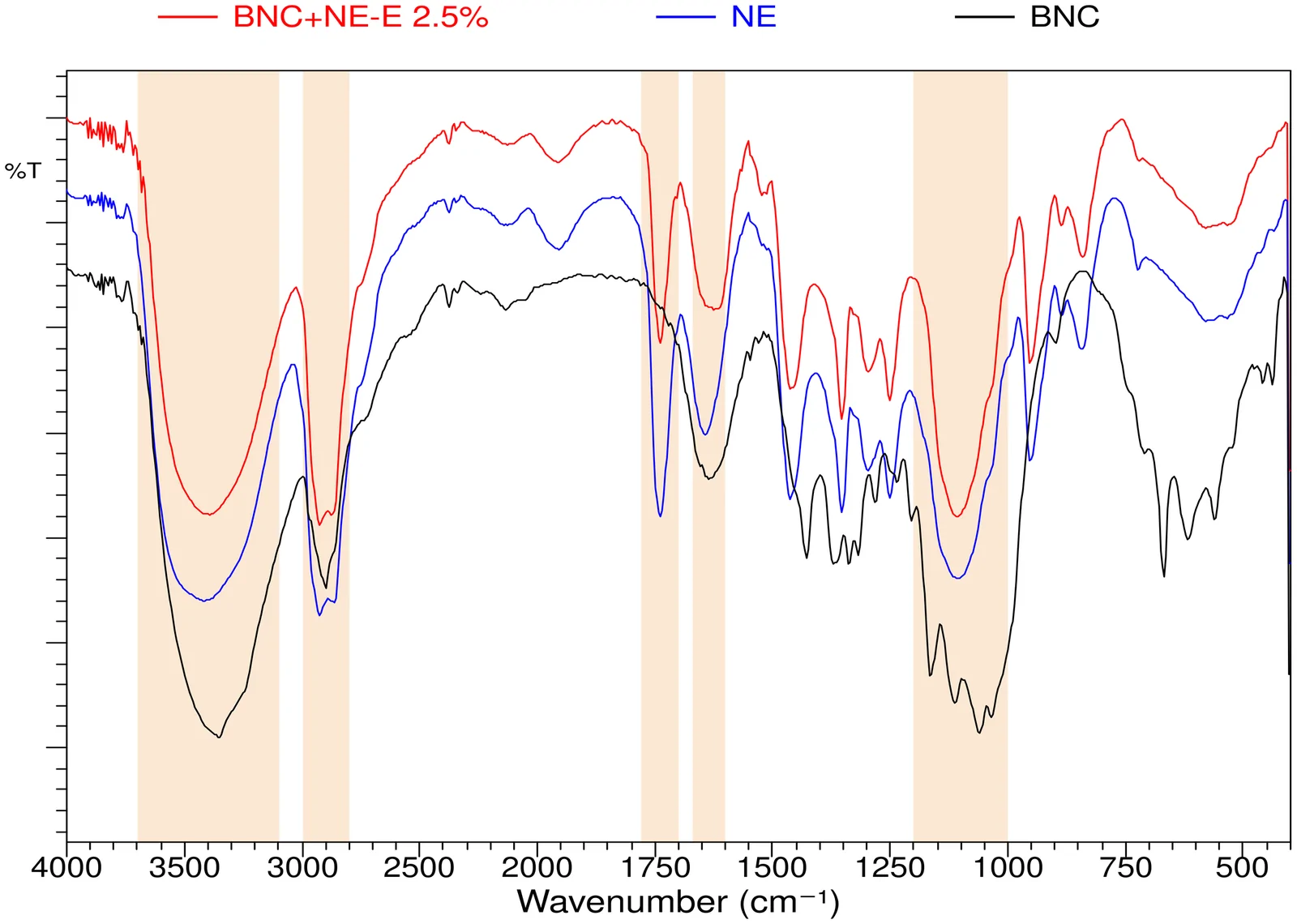
FT-IR spectrum of BNC and BNC-based nanoemulsion hydrogel.

The NE spectrum exhibited distinct absorption bands at approximately 2924, 2856, 1955, 1737, 950, 885, 842 cm ⁻ ¹, which are attributed to the stretching vibrations of C-H and C = O stretching. In the BNC + NE spectrum, these bands were still observed, although with altered intensities, indicating the successful incorporation of the oil phase into the BNC-based system. The absorption band at 1737 cm^-1^, which indicates the C = O group derived from avocado seed oil, was absent in the pure BNC spectrum, confirming that its presence serves as a marker for the successful incorporation of the extract-containing nanoemulsion into the system. A band observed at approximately 1633 cm ⁻ ¹ is associated with aromatic C = C stretching vibrations or bound water vibrations in BNC. The shift of this band in the BNC + NE spectrum suggests interactions between polyphenolic components of the avocado seed extract and the BNC structure.

### In-vitro skin permeation study

Permeation studies were conducted to compare the cumulative penetration ability of two hydrogel formulations, namely BNC combined with a conventional emulsion (BNC + E) and BNC combined with a nanoemulsion (BNC + NE), both containing avocado seed extract (Fig 8).

**Fig 8:**
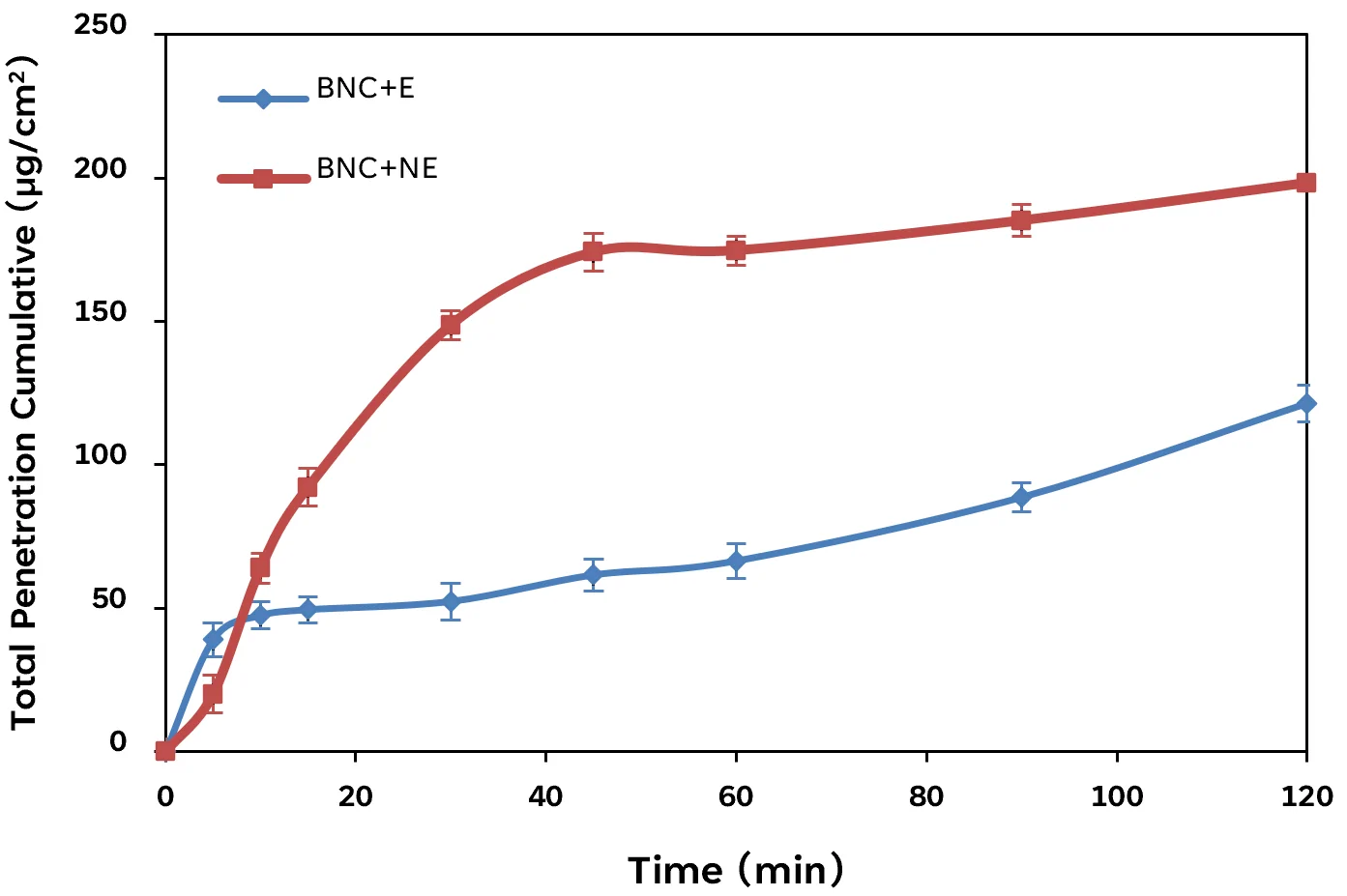
In-vitro diffusion profile of BNC-based nanoemulsion hydrogel.

As shown in the diffusion profile, the BNC + NE formulation consistently exhibited a higher penetration rate and cumulative penetration than BNC + E throughout the observation period. At 10 minutes, the cumulative penetration of BNC + NE reached 63.94 ± 0.60 µg/cm^2^, whereas BNC + E showed 47.47 ± 0.71 µg/cm^2^. This difference became more pronounced at 30 minutes, where BNC + NE reached 148.69 ± 0.54 µg/cm^2^, compared to 52.34 ± 0.43 µg/cm^2^ for BNC + E. After 120 minutes, the cumulative penetration of BNC + NE further increased to 198.28 ± 0.54 µg/cm^2^, while BNC + E reached 121.35 ± 0.54 µg/cm^2^.These findings demonstrate that incorporating avocado seed extract into a nanoemulsion-based system enhances its penetration performance compared with a conventional emulsion system, suggesting the advantage of nanoscale delivery for improving diffusion.

### In-vivo evaluation of anti-aging

#### Macroscopic evaluation of dorsal skins.

Based on the Fig 9, UVB irradiation for 4 weeks significantly affected rat skin, as indicated by an increase in the visual wrinkle score in the model control group from 1 to 4.5 ± 0.6. The results also demonstrate that treatment with the tested formulations affected one anti-aging parameter, namely wrinkle formation. Macroscopic observation revealed that all treatment groups prevented wrinkle formation on the dorsal skin of rats after 4 weeks of UVB exposure.

**Fig 9:**
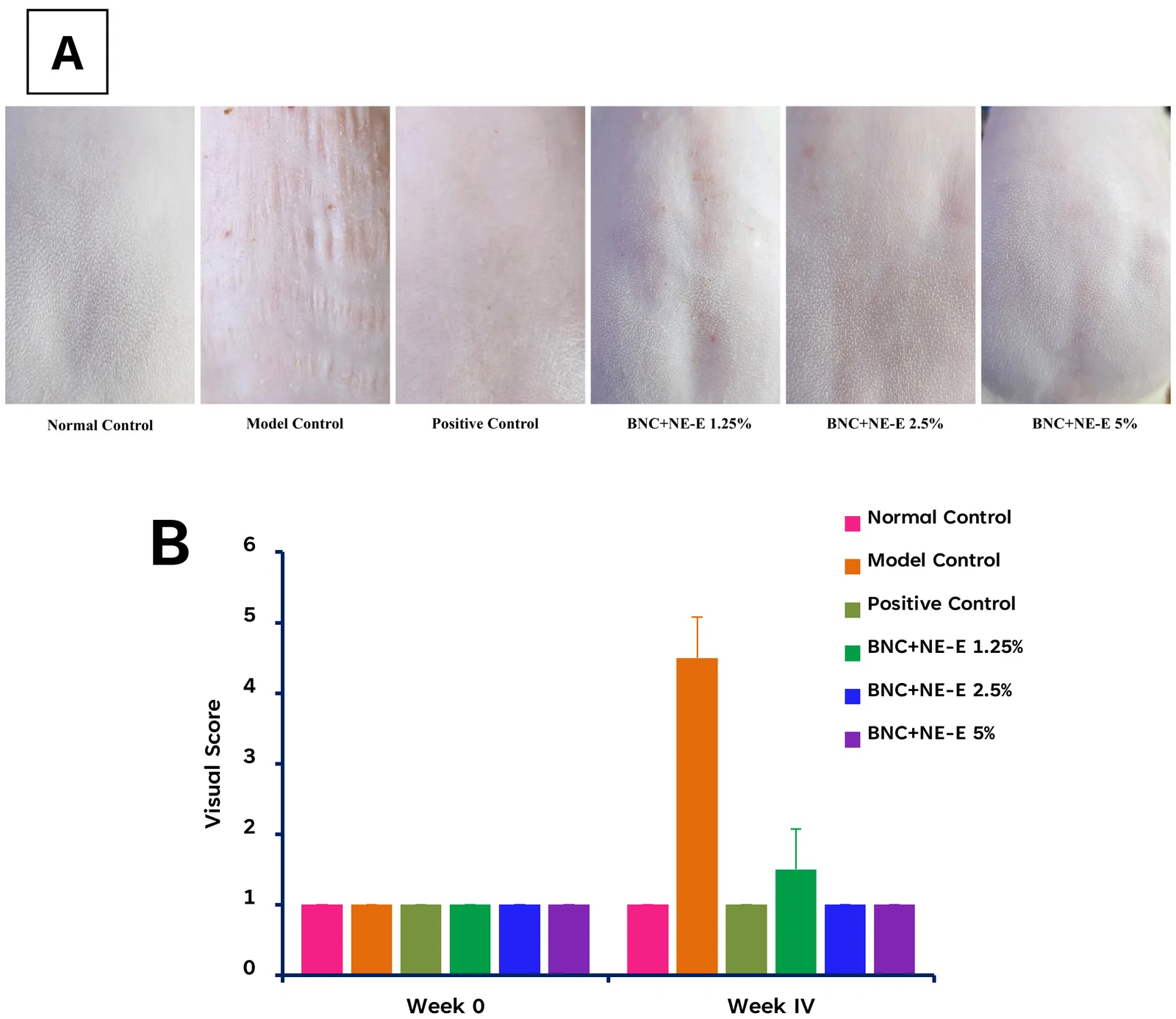
Macroscopic evaluation of dorsal skins. (a) Physical appearances of UV-irradiated rats with various treatments at the last week; (b) Results of the visual score of different experimental groups at the last week. All data shown are mean±SD of 4 rats; p < 0,05.

#### Evaluation of skin elasticity using the pinch test.

Skin elasticity was assessed using the pinch test by measuring the recovery time required for the dorsal skin to return to its original position. The normal control group maintained relatively stable recovery times, indicating normal skin elasticity. In contrast, model control group exhibited a progression in recovery time throughout observation period, indicating reduced skin elasticity as a result of UVB exposure (Fig 10). Compared with model control, rats in BNC + NE group showed significant reductions in recovery time compared to model control group, showed that BNC + NE could promote the skin elasticity.

**Fig 10:**
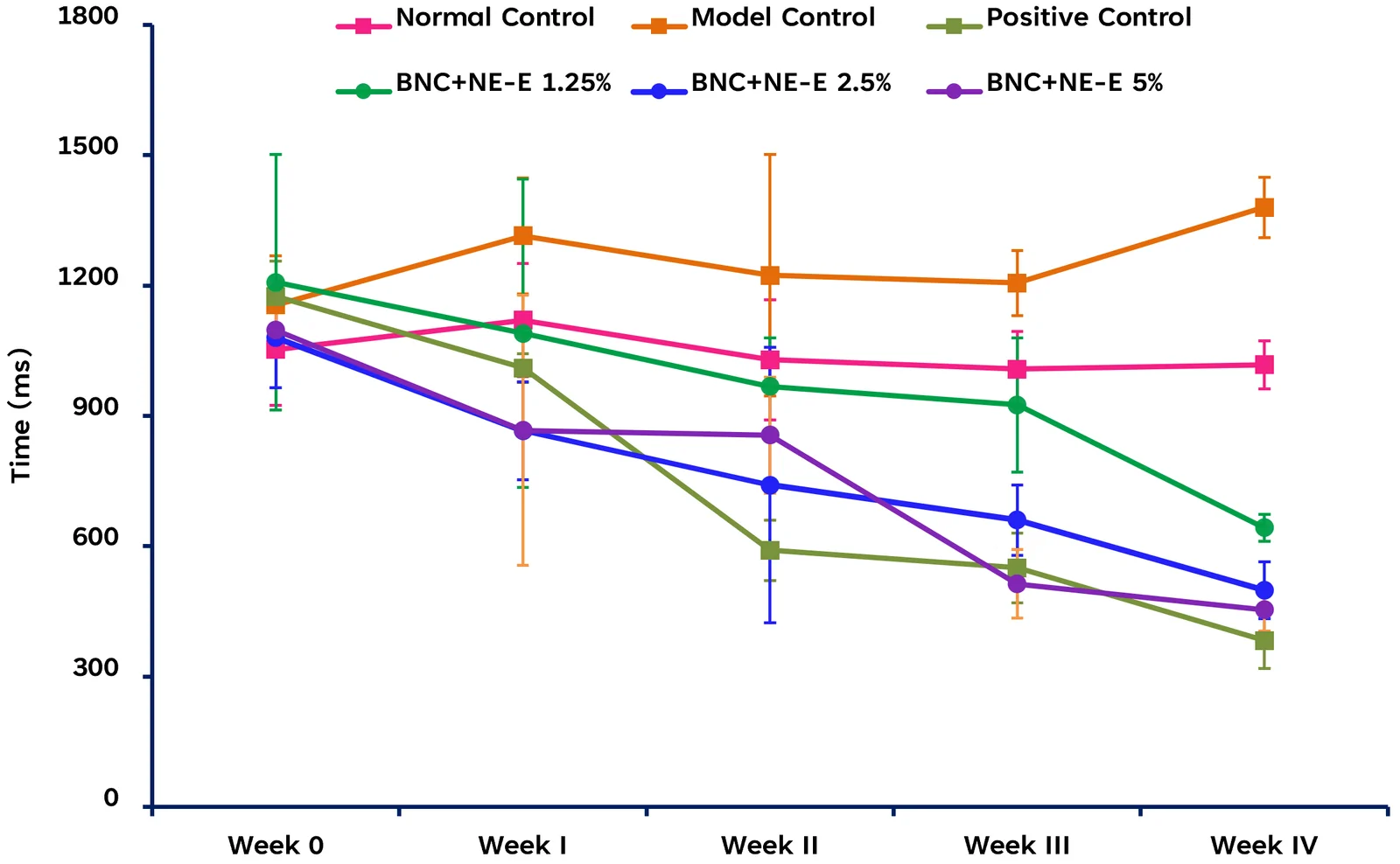
Evaluation of skin elasticity based on pinch test recovery time in UVB-induced photoaging rats treated with BNC-based nanoemulsion hydrogel. All data shown are mean±SD of 4 rats.

#### Skin hydration measurement.

The results showed the percentage of in vivo skin hydration measurement among the treatment groups (Fig 11). Compared to normal group, model group exhibited decreased skin hydration with 31.29% and 27.91%, respectively. Positive control All BNC + NE-E treated groups showed increased skin hydration compared to the model group, with 29.09%, 31.41%, and 31.97% of skin hydration level alongside an increase of extract concentration. However, each group showed no statistically significant in skin hydration.

**Fig 11:**
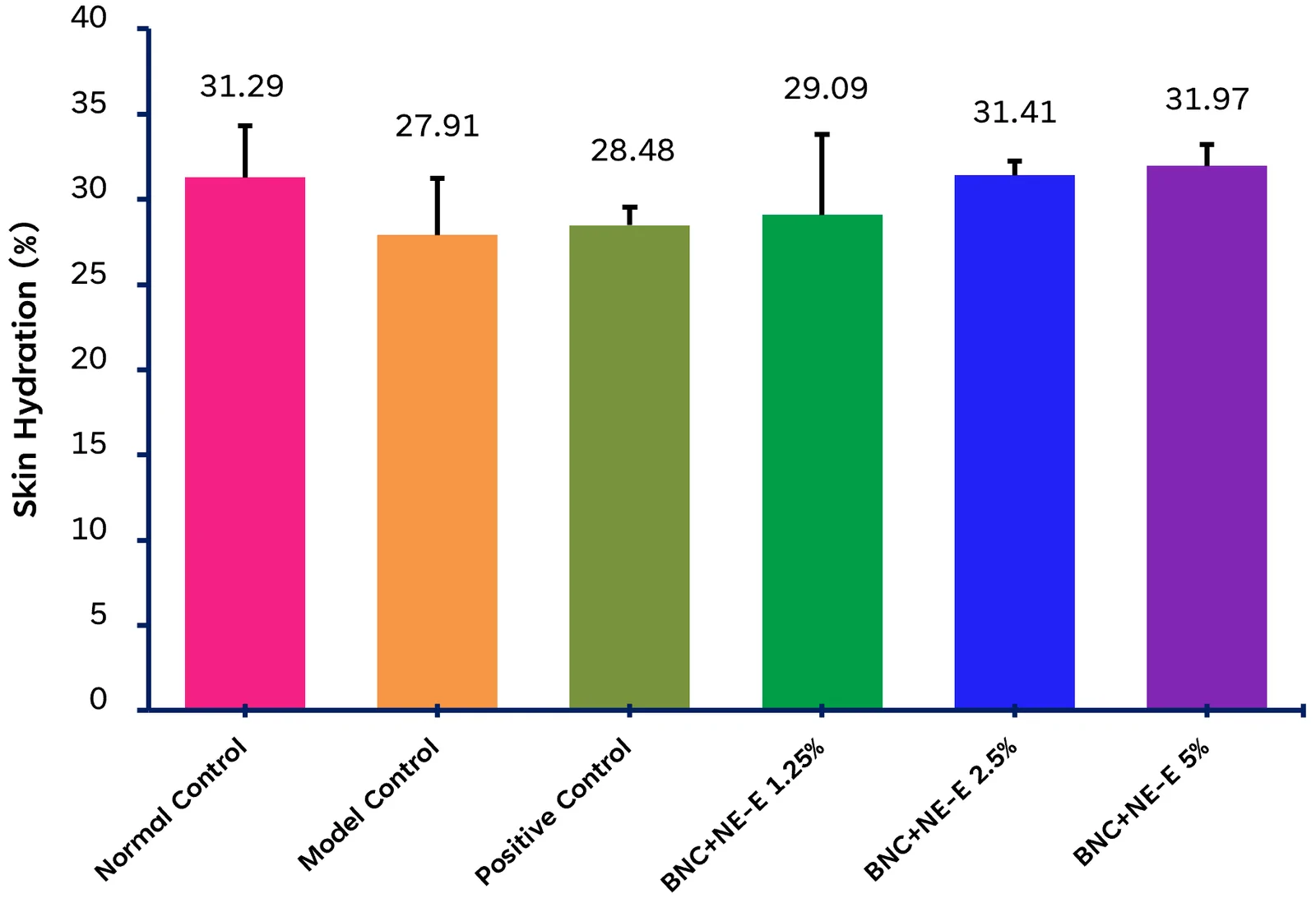
Evaluation of skin hydration of UV irradiation on rats. All data shown are the mean±SD of 4 rats. One-way ANOVA (*p* < 0,05) with *post hoc* Tukey’s multiple comparison test was used for statistical analysis.

#### Analysis of collagen density.

The histological study of the normal control group exhibited a relatively loose dermal structure with abundant collagen fibers (Fig 12A). Following UVB exposure, the model control group showed marked histological alterations with significant reduction in collagen density. In contrast, the positive control group and all BNC-based nanoemulsion extract (BNC + NE-E) treatment groups demonstrated improved collagen density compared to the model control. An increase in collagen intensity and a progressively denser dermal structure were observed with increasing concentrations of avocado seed extract.Tissue sections from the positive control group and the BNC-based nanoemulsion treatment group at 5% concentration displayed a broader distribution of collagen fibers compared with the other treatment groups.

**Fig 12:**
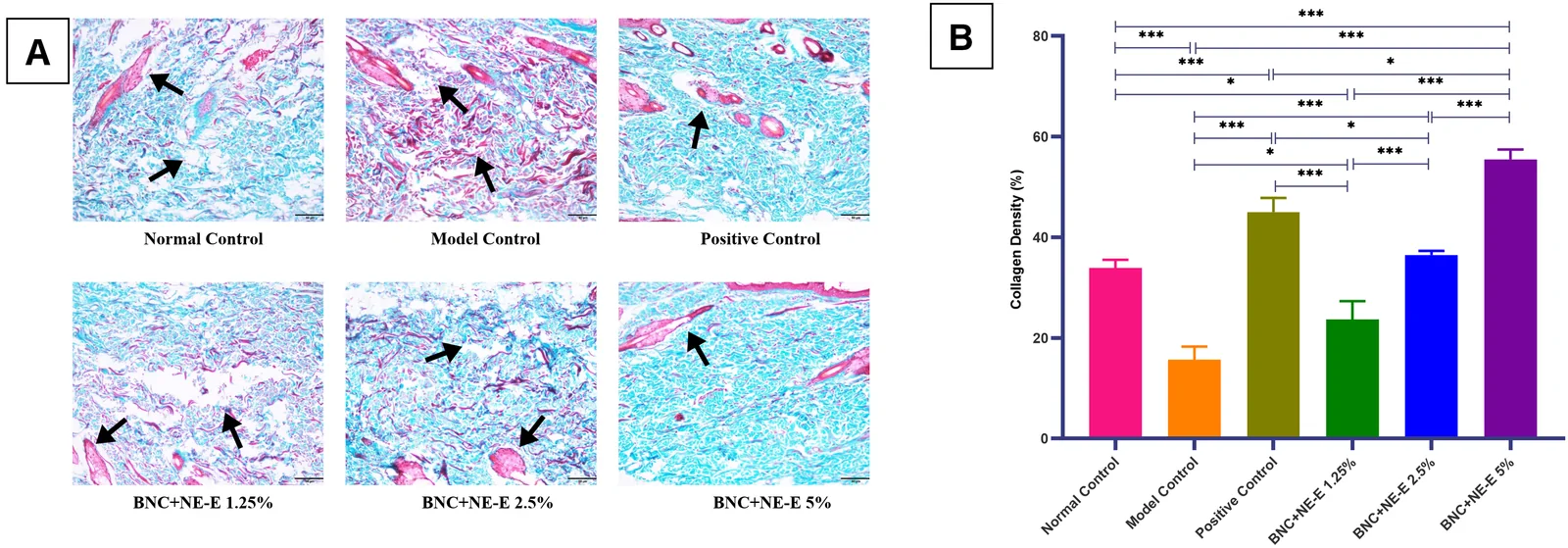
Evaluation of collagen density. (a) Rat skin tissue observed by Masson’s trichrome staining. (b)Percentage of collagen density. All data shown are mean±SD of 4 rats; *p < 0,05 and ***p < 0.0001. One-way ANOVA with *post hoc* Tukey’s multiple comparison test was used for statistical analysis.

Collagen density was assessed by green color intensity and quantified as a percentage (%) using ImageJ software, as shown in Fig 12B. Significant differences in collagen density were observed among the treatment groups, with the model control group showing the lowest collagen density. The BNC + NE-E 1.25% group exhibited lower collagen density compared to the normal control, whereas the BNC + NE-E 2.5% and 5% groups showed higher collagen density values than the normal control group. Statistical analysis revealed a significant difference (p < 0.0001) between the model control group and all treatment groups.Each BNC + NE-E formulation exhibited significantly different collagen density values across concentrations. The BNC + NE-E 2.5% group showed no significant difference compared with the normal control, suggesting that formulation restored UVB-exposed skin to normal conditions. In contrast, the BNC + NE-E 5% formulation demonstrated a significantly higher collagen density than the normal control, indicating not only recovery from UVB-induced damage but also an enhancement of dermal structural integrity.

## Discussion

The identified compounds belonged to various chemical classes, including phenolic compounds, fatty acid derivatives, amino acid derivatives, alkaloid-related structures, and lipid-related molecules. Among the detected compounds, procyanidin which is the polymer of catechin and epictachinwas identified as a prominent polyphenolic compound [37]. It has been widely reported to exhibit strong antioxidant properties and protective effects against oxidative stress-induced skin damage [38–40]. Several fatty acid derivatives, i.e., heptadecadienoic acid and hexadecanedioic acid were also observed. The presence of fatty acid derivatives may support the skin barrier and enhance hydration, both of which are essential factors in preventing premature aging [41]. These findings suggest that the observed biological effects of the formulation may arise from the combined action of multiple bioactive constituents rather than a single compound.

The nanoemulsion exhibited a clear, transparent appearance, attributed to its globule diameter being significantly smaller than the wavelength of visible light, resulting in minimal light scattering [42]. Avocado seed oil is inherently lipophilic, with an HLB value of approximately 7, was successfully formulated using Cremophor® RH40, a non-ionic surfactant with an HLB value of 14–16, and PEG 400 as a co-surfactant. This surfactant system effectively reduced interfacial tension and promoted spontaneous nanoemulsion formation, resulting in a physically stable system [43–46].

The nanoscale size and spherical morphology increase the surface area available for skin contact, thereby facilitating enhanced interaction with the membrane and promoting diffusion of active compounds. This mechanistic interpretation is in agreement with the diffusion study outcomes and the observed in vivo anti-aging performance. Similar structural characteristics have also been reported for Cremophor® RH40–PEG 400–based nanoemulsion system, supporting the stability and effectiveness of this formulation approach [47].

Particle size distribution and morphological analyses further confirmed the uniformity and stability of the nanoemulsion system. The low PDI values obtained indicate a relatively homogeneous dispersion and good physical stability [48]. These results correlated well with TEM observations, which showed uniformly distributed, spherical-to-oval nanoemulsion droplets with well-defined interfacial layers, indicating effective oil encapsulation and minimal aggregation [49]. Consistent with these findings, the freeze–thaw stability test showed that globule size and PDI remained relatively stable up to the last cycle, confirming the robustness of the nanoemulsion system.

The incorporation of the nanoemulsion into the BNC matrix produced a transparent hydrogel, despite the presence of humectants, attributable to the nanofibrillar BNC network forming a three-dimensional structure with nanoscale pores that minimized light scattering [50]. Increased viscosity was associated with reduced spreadability, consistent with rheological behavior. However, the formulations maintained adequate spreadability to ensure uniform skin coverage and a lightweight sensory profile. Furthermore, the pH values of all formulations remained within the physiological skin range, indicating suitability for prolonged topical application [51].

FT-IR analysis of the BNC-based nanoemulgel revealed slight changes in the absorption bands corresponding to –OH, C-H, C = O, and C-O-C groups, suggesting physicochemical interactions such as hydrogen bonding and possible hydrophobic interactions between the bioactive compounds of the extract (such as phenolics and triglycerides) and the hydroxyl groups of BNC [52]. The absence of new absorption bands confirms that these interactions were predominantly physical rather than covalent. Such interactions indicate successful incorporation of the nanoemulsion into the BNC matrix and support its potential application as a stable topical delivery system for pharmaceutical or cosmetic formulations [53].

The enhanced diffusion behavior observed with the BNC + NE formulation can be attributed to the small, homogeneous droplets of the nanoemulsion, which facilitate penetration of active compounds across the diffusion membrane. Nanoemulsions with particle size below 80 nm can efficiently fill the whole hair follicle canals, reach underneath the dermal surfaces, and diffuse into the surrounding dermal tissues by temporarily alter lipid organization within membrane, thereby increasing their availability [54,55]. In contrast, the conventional emulsion system (BNC + E), characterized by larger droplet sizes, exhibited slower diffusion behavior and lower cumulative penetration. Overall, these findings suggest that incorporation of a nanoemulsion system enhances the diffusion performance of avocado seed extract in BNC-based formulations [56,57].

Furthermore, the BNC matrix itself may prolong the residence time of nanoemulsion droplets on the membrane surface, enabling sustained diffusion. Similar reservoir-like behavior and prolonged release effects have been reported for nanocellulose-based hydrogel systems in topical delivery applications [58]. Although the diffusion study was conducted using a synthetic membrane, it provides a reliable preliminary assessment of comparative penetration performance between formulations [59].

The in vivo findings support the enhanced delivery performance observed in the diffusion study. UVB irradiation successfully induced photoaging characteristics, as evidenced by increased wrinkle formation, reduced skin hydration, and decreased collagen density in the model control group, consistent with previous reports on UVB-induced skin photoaging and barrier disruption [60,61]. In contrast, treatment with the BNC-based nanoemulsion containing avocado seed extract reduced wrinkle formation and improved overall skin appearance, indicating a protective effect against UVB-induced skin damage.

Treatment with the BNC-based nanoemulsion hydrogel resulted in a gradual decrease in recovery time across all concentrations, suggesting improved skin elasticity. At the end of the treatment period, the 5% formulation exhibited the shortest recovery time among all treated groups, approaching or surpassing the normal control group. These findings suggest that the BNC-based nanoemulsion containing avocado seed extract effectively improved skin elasticity in a concentration-dependent manner and mitigated UVB-induced loss of skin elasticity.Skin elasticity assessment using the pinch test further demonstrated the formulation's protective effect with reduced recovery time observed particularly in the 2.5% and 5% groups, reflecting improved skin elasticity. Enhanced penetration by the nanoemulsion system and prolonged contact time supported by BNC matrix may contribute to this effect [62–64].

Skin hydration measurements showed a decreasing trend in the model control group compared with the normal control, reflecting impairment of the skin barrier following UVB exposure. Treatment with the BNC-based nanoemulsion demonstrated a concentration-dependent increase in hydration values, with the 2.5% and 5% formulations approaching normal skin conditions. Although statistical differences were not significant, the observed trend suggests a protective effect against UVB-induced dehydration, potentially associated with the high water-retention capacity of the BNC matrix and enhanced delivery of bioactive compounds [65–67].

Histological evaluation demonstrated that UVB exposure markedly reduced dermal collagen density, whereas treatment with the BNC-based nanoemulsion significantly restored collagen levels compared with the model control. UV-induced reactive oxygen species (ROS) are known to upregulate matrix metalloproteinases (MMPs), resulting in accelerated collagen degradation [68]. The 2.5% formulation restored collagen density, while the 5% formulation produced the highest collagen deposition. Collagen improvement is attributed to antioxidant which scavenge ROS, reduce oxidative stress, inhibit MMP-1, MMP-3, and MMP-9, and protect dermal fibroblasts, thereby promoting type I collagen synthesis [69]. Quantitative ImageJ analysis was consistent with the histological observations, confirming the significant influence of BNC-based nanoemulsion treatment on collagen density.

Beyond the contribution of the bioactive compounds, the enhanced in vivo efficacy may also be attributed to improved skin penetration facilitated by the nanoscale globule size and low PDI of the nanoemulsion system. Increased penetration enhances bioavailability, thereby amplifying in vivo therapeutic outcomes [59]. Similar findings have been reported in previous studies, in which nanoemulsion-based formulations significantly improved topical efficacy by enhancing penetration compared with conventional systems [64]. Bacterial nanocellulose (BNC) serves as an effective delivery matrix, enhancing formulation retention and prolonging skin contact [19]. Its synergistic integration with nanoemulsion technology improves anti-aging efficacy by enhancing skin hydration and dermal penetration.

## Conclusion

This study demonstrated that a BNC-based nanoemulsion hydrogel formulation loaded with *Persea americana* seed extract provides a stable, biocompatible, and effective anti-aging. The nanoemulsion precursor exhibited favorable physicochemical properties, preserved key bioactive compounds, and remained stable under stress thermal conditions. The nanoemulsion was successfully adsorbed into the BNC matrix, demonstrating the desired physicochemical characteristics and enhancing in vitro diffusion performance by more than 1.5-fold compared with the BNC loaded with a conventional emulsion. Notably, the BNC-based nanoemulsion hydrogel demonstrated superior collagen preservation compared to the model control, indicating its potential as an effective natural alternative for anti-aging applications. Overall, the findings support the BNC-based nanoemulsion loaded with *Persea americana* seed extract as a promising plant by-product and alternative strategy for anti-aging and improving skin integrity.

## Supporting information

S1 Table Formulation development and selection framework for the BNC-based nanoemulsion hydrogel: material variables, process conditions, quality attributes, and selection considerations. (DOCX)
