# Supplementary material for: Formulation and biological evaluation of a bacterial nanocellulose-based nanoemulsion hydrogel loaded with Persea americana Mill. seed extract: An anti-aging assessment
Source: PLoS One. 2026 Nov 5;21(11):e0359807. doi: 10.1371/journal.pone.0359807 (PMC13739224; doi:10.1371/journal.pone.0359807)

PLOS One

Formulation and biological evaluation of a bacterial nanocellulose-based nanoemulsion loaded with Persea americana Mill. seed extract: An anti-aging assessment

--Manuscript Draft--

**Manuscript Number:**	PONE-D-26-10500
**Article Type:**	Research Article
**Full Title:**	Formulation and biological evaluation of a bacterial nanocellulose-based nanoemulsion loaded with Persea americana Mill. seed extract: An anti-aging assessment
**Short Title:**	Formulation and anti-aging assessment of a BNC-based nanoemulsion loaded with avocado seed extract
**Corresponding Author:**	Heni Rachmawati, Doctor<br>Bandung Institute of Technology: Institut Teknologi Bandung<br>Bandung, West Java INDONESIA
**Keywords:**	anti-aging; avocado seed; bacterial nanocellulose (BNC); collagen density; nanoemulsion
**Abstract:**	Avocado (Persea americana Mill.) seed is an underutilized by-product rich in catechins and fatty acids, with potential anti-aging properties. However, the utilization of avocado seed in cosmetic formulations is limited by its poor solubility, physicochemical instability, and minimal skin penetration. Integrating nanoemulsion with bacterial nanocellulose (BNC) represents a practical approach to enhance bioactive solubilization, skin penetration, and formulation stability while providing a biocompatible, highly hydrated matrix for advanced topical delivery. Therefore, this study aimed to develop a BNC-based nanoemulsion incorporating avocado seed extract, determine its physicochemical characteristics, stability, skin penetration, and in vivo anti-aging activity. Physicochemical characteristics were evaluated by particle size, polydispersity index (PDI), transmittance, pH, viscosity, morphology (TEM), and FT-IR. Physical stability was assessed by phase separation, particle size, polydispersity index, and pH using a freeze–thaw method for three cycles. The diffusion study was conducted using Franz diffusion cells, and anti-aging activities were assessed using the pinch test, skin hydration, and collagen density assesment. The nanoemulsion exhibited favorable nanoscale characteristics, remained stable over three freeze–thaw cycles, penetrate over 1.5-fold greater compared to BNC-based conventional emulsion, and showed significant improvements in pinch test recovery, skin hydration, and collagen density ($p < 0.05$), indicating notable anti-aging efficacy. The developed BNC-based nanoemulsion serum highlights the potential of avocado seed as an effective topical carrier for anti-aging cosmetic applications.
**Order of Authors:**	Diah Ramadhani, M.Si
	Tri Suciati, Doctor
	Elfahmi Elfahmi, Doctor
	Ajeng Rana Wulan Sujilah, M.S.Pharm
	Heni Rachmawati, Doctor
**Additional Information:**	
**Question**	**Response**
**Financial Disclosure**<br><br>Enter a financial disclosure statement that describes the sources of funding for the work included in this submission. Review the submission guidelines for detailed requirements. View published research articles from *PLOS ONE* for specific	Yes

Powered by Editorial Manager® and ProduXion Manager® from Aries Systems Corporation

examples.<br><br>This statement is required for submission and **will appear in the published article** if the submission is accepted. Please make sure it is accurate.<br><br>**Funded studies**<br>Enter a statement with the following details:<br>• Initials of the authors who received each award<br>• Grant numbers awarded to each author<br>• The full name of each funder<br>• URL of each funder website<br>• Did the sponsors or funders play any role in the study design, data collection and analysis, decision to publish, or preparation of the manuscript?<br><br>Did you receive funding for this work?	
Please add funding details.<br>as follow-up to "**Financial Disclosure**<br><br>Enter a financial disclosure statement that describes the sources of funding for the work included in this submission. Review the submission guidelines for detailed requirements. View published research articles from *PLOS ONE* for specific examples.<br><br>This statement is required for submission and **will appear in the published article** if the submission is accepted. Please make sure it is accurate.<br><br>**Funded studies**<br>Enter a statement with the following details:<br>• Initials of the authors who received each award<br>• Grant numbers awarded to each author<br>• The full name of each funder<br>• URL of each funder website<br>• Did the sponsors or funders play any role in the study design, data collection and analysis, decision to publish, or preparation of the manuscript?	This work was supported by Beasiswa Pendidikan Indonesia (BPI) (Grant No. 202101121555), Center for Higher Education Funding and Assessment (PPAPT) under the Ministry of Higher Education, Science, and Technology, Indonesia and the Indonesia Endowment Funds for Education (LPDP) under the Ministry of Finance, Indonesia.

Powered by Editorial Manager® and ProduXion Manager® from Aries Systems Corporation

Did you receive funding for this work?"	
Please select the country of your main research funder (please select carefully as in some cases this is used in fee calculation).<br>as follow-up to "**Financial Disclosure**<br><br>Enter a financial disclosure statement that describes the sources of funding for the work included in this submission. Review the submission guidelines for detailed requirements. View published research articles from *PLOS ONE* for specific examples.<br><br>This statement is required for submission and **will appear in the published article** if the submission is accepted. Please make sure it is accurate.<br><br>**Funded studies**<br>Enter a statement with the following details:<br>• Initials of the authors who received each award<br>• Grant numbers awarded to each author<br>• The full name of each funder<br>• URL of each funder website<br>• Did the sponsors or funders play any role in the study design, data collection and analysis, decision to publish, or preparation of the manuscript?<br><br>Did you receive funding for this work?"	INDONESIA - ID
**Competing Interests**<br><br>Use the instructions below to enter a competing interest statement for this submission. On behalf of all authors, disclose any competing interests that could be perceived to bias this work—acknowledging all financial support and any other relevant financial or non-financial competing interests.	The authors have declared that no competing interests exist.

This statement is required for submission and **will appear in the published article** if the submission is accepted. Please make sure it is accurate and that any funding sources listed in your Funding Information later in the submission form are also declared in your Financial Disclosure statement.<br><br>View published research articles from *PLOS ONE* for specific examples.<br><br>**NO authors have competing interests**<br><br>Enter: *The authors have declared that no competing interests exist.*<br><br>**Authors with competing interests**<br><br>Enter competing interest details beginning with this statement:<br><br>*I have read the journal's policy and the authors of this manuscript have the following competing interests: [insert competing interests here]*<br><br>* typeset	
**Ethics Statement**<br><br>Enter an ethics statement for this submission. This statement is required if the study involved:<br><br>• Human participants<br>• Human specimens or tissue<br>• Vertebrate animals or cephalopods<br>• Vertebrate embryos or tissues<br>• Field research<br><br>Write "N/A" if the submission does not require an ethics statement.<br><br>General guidance is provided below. Consult the submission guidelines for detailed instructions. **Make sure that all information entered here is included in the Methods section of the manuscript.**	This study was approved by The Ethics Committee for Animal Research of Institute of Technology Bandung with regard to the protection and welfare of animals used in this research (Reference No. KEP/I/2024/III/H310124DR/BANA).

**Format for specific study types**<br><br>**Human Subject Research (involving human participants and/or tissue)**<br>• Give the name of the institutional review board or ethics committee that approved the study<br>• Include the approval number and/or a statement indicating approval of this research<br>• Indicate the form of consent obtained (written/oral) or the reason that consent was not obtained (e.g. the data were analyzed anonymously)<br><br>**Animal Research (involving vertebrate animals, embryos or tissues)**<br>• Provide the name of the Institutional Animal Care and Use Committee (IACUC) or other relevant ethics board that reviewed the study protocol, and indicate whether they approved this research or granted a formal waiver of ethical approval<br>• Include an approval number if one was obtained<br>• If the study involved *non-human primates*, add *additional details* about animal welfare and steps taken to ameliorate suffering<br>• If anesthesia, euthanasia, or any kind of animal sacrifice is part of the study, include briefly which substances and/or methods were applied<br><br>**Field Research**<br><br>Include the following details if this study involves the collection of plant, animal, or other materials from a natural setting:<br>• Field permit number<br>• Name of the institution or relevant body that granted permission	
**Data Availability**<br><br>Authors are required to make all data underlying the findings described fully available, without restriction, and from the time of publication. PLOS allows rare exceptions to address legal and ethical concerns. See the PLOS Data Policy and FAQ for detailed information.	Yes - all data are fully available without restriction

A Data Availability Statement describing where the data can be found is required at submission. Your answers to this question constitute the Data Availability Statement and **will be published in the article**, if accepted.<br><br>**Important:** Stating 'data available on request from the author' is not sufficient. If your data are only available upon request, select 'No' for the first question and explain your exceptional situation in the text box.<br><br>Do the authors confirm that all data underlying the findings described in their manuscript are fully available without restriction?	
**Describe where the data may be found in full sentences. If you are copying our sample text, replace any instances of XXX with the appropriate details.**<br><br>• If the data are **held or will be held in a public repository**, include URLs, accession numbers or DOIs. If this information will only be available after acceptance, indicate this by ticking the box below. For example: *All XXX files are available from the XXX database (accession number(s) XXX, XXX.*).<br>• If the data are all contained **within the manuscript and/or Supporting Information files**, enter the following: *All relevant data are within the manuscript and its Supporting Information files.*<br>• If neither of these applies but you are able to provide **details of access elsewhere**, with or without limitations, please do so. For example:<br><br>*Data cannot be shared publicly because of [XXX]. Data are available from the XXX Institutional Data Access / Ethics Committee (contact via XXX) for researchers who meet the criteria for access to confidential data.*<br><br>*The data underlying the results presented in the study are available*	All relevant data are within the manuscript and its Supporting Information files.

from (include the name of the third party and contact information or URL).<br>• This text is appropriate if the data are owned by a third party and authors do not have permission to share the data.<br><br>* typeset	
Additional data availability information:	

Manuscript Click here to access/download;Manuscript;PLOS one manuscript draft nanoavocado 4-3-2026.docx

Formulation and biological evaluation of a bacterial nanocellulose-based nanoemulsion loaded with *Persea americana* Mill. seed extract: An anti-aging assessment

Diah Ramadhani[1,2], Tri Suciati[1], Elfahmi[4], Ajeng Rana Wulan Sujilah[1], Heni Rachmawati[1, 3*]

[1] Department of Pharmaceutics, School of Pharmacy, Bandung Institute of Technology, Bandung, West Java, Indonesia

[2] Faculty of Pharmacy, Universitas 17 Agustus 1945 Jakarta, Jakarta, Indonesia

[3] Research Center for Nanoscience and Nanotechnology, Bandung Institute of Technology, Bandung, West Java, Indonesia

[4] Department of Pharmaceutical Biology, School of Pharmacy, Bandung Institute of Technology, Bandung, West Java, Indonesia

* Corresponding author

E-mail: h_rachmawati@itb.ac.id (HR)

Abstract

Avocado (*Persea americana* Mill.) seed is an underutilized by-product rich in catechins and fatty acids, with potential anti-aging properties. However, the utilization of avocado seed in cosmetic formulations is limited by its poor solubility, physicochemical instability, and minimal skin penetration. Integrating nanoemulsion with bacterial nanocellulose (BNC) represents a practical approach to enhance bioactive solubilization, skin penetration, and formulation stability while providing a biocompatible, highly hydrated matrix for advanced topical delivery. Therefore, this study aimed to develop a BNC-based nanoemulsion incorporating avocado seed extract, determine its physicochemical characteristics, stability, skin penetration, and in vivo anti-aging activity. Physicochemical characteristics were evaluated by particle size, polydispersity index (PDI), transmittance, pH, viscosity, morphology (TEM), and FT-IR. Physical stability was assessed by phase separation, particle size, polydispersity index, and pH using a freeze–thaw method for three cycles. The diffusion study was conducted using Franz diffusion cells, and anti-aging activities were assessed using the pinch test, skin hydration, and collagen density assesment. The nanoemulsion exhibited favorable nanoscale characteristics, remained stable over three freeze–thaw cycles, penetrate over 1.5-fold greater compared to BNC-based conventional emulsion, and showed significant improvements in pinch test recovery, skin hydration, and collagen density ($p < 0.05$), indicating notable anti-aging efficacy. The developed BNC-based nanoemulsion serum highlights the potential of avocado seed as an effective topical carrier for anti-aging cosmetic applications.

Introduction

Avocado (*Persea americana* Mill.) is a fruit with high nutritional value, characterized by a high content of bioactive compounds, vitamins, and fatty acids. However, the utilization of

avocado is generally limited to its pulp, while the seeds are discarded as household and food-industry waste. In addition, avocado seeds are rich in bioactive compounds, including phenolic compounds, flavonoids, vitamin C, vitamin E, and approximately 15% of fatty acids [1]. Avocado seeds have been reported to exhibit potent antioxidant activity by scavenging DPPH radicals, inhibiting lipid oxidation, and neutralizing hydrogen peroxide [2]. The antioxidant activity of avocado seeds has been attributed to the presence of catechin and epicatechin, 3-*O*-caffeoylquinic acid (a chlorogenic acid isomer), and glutathione [3,4]. The consumption or topical application of antioxidants is recognized as an effective strategy to prevent premature aging by protecting collagen- and elastin-forming proteins from oxidative damage [5].

Photoaging, or premature skin aging induced by ultraviolet (UV) radiation, is a significant factor that stimulates the production of matrix metalloproteinases (MMPs), leading to the degradation of collagen fibers in the dermal layer [6]. This condition is particularly prevalent in tropical countries such as Indonesia, where high levels of solar radiation lead to frequent UV exposure. Althoug numerous cosmetic products claim anti-aging effects, effective delivery of active antioxidant compounds to deeper layers of the skin remains a major challenge [7].

Nanoemulsions are particularly suitable for cosmetic applications due to their high stability, low viscosity, and transparent appearance. Their large surface area facilitates efficient delivery of active compounds to penetrate the stratum corneum effectively, thereby enhancing the bioavailability of active ingredients [8]. Emulsion-type serums, particularly oil-in-water (o/w) systems, are advantageous for incorporating lipophilic active compounds while maintaining a non-greasy texture suitable for facial application and exhibit superior absorption and deeper skin penetration [9]. One of the most critical factors in serum formulation is the selection of an appropriate gelling agent, which determines the consistency, stability, spreadability, and overall performance [10].

Conventional gelling agents such as hydroxypropyl methylcellulose (HPMC) and sodium carboxymethyl cellulose (CMC-Na) are widely used; however, they mainly function as structural components and exhibit limited bioactivity [11–15]. An alternative to conventional gelling agents is the use of bacterial nanocellulose (BNC) [16] which is biopolymer characterized by high biocompatibility, excellent water-holding capacity, superior film-forming ability, and a unique three-dimensional nanofibrillar network [17,18]. These properties enable BNC not only to function as a gelling agent but also to serve as an innovative topical carrier that enhances skin hydration, prolongs residence time on the skin, and supports the controlled release of active compounds [19].

Despite the reported antioxidant potential of avocado seeds, studies on the development of avocado seed extract as topical nanoemulsions remain limited. Moreover, there is a scarcity of research integrating avocado seed nanoemulsions with BNC-based matrices for anti-aging applications, particularly with respect to in vivo evaluation against UVB-induced photoaging. Therefore, we hypothesized that incorporation of avocado seed extract nanoemulsion into a BNC-based serum would enhance skin penetration and improve anti-aging efficacy. This study aimed to develop and characterize a nanoemulsion-based serum containing avocado seed extract and oil within a BNC matrix and to evaluate its skin penetration and anti-aging activity in a UVB-induced photoaging rat model.

Materials and methods

Preparation of avocado seed extracts

Avocados (Lembang, West Java, Indonesia) were cut in half, and the seeds were separated and cleaned. The seeds were dried in a drying oven at 60 °C for at least five days and subsequently ground into a powder using a grinder. The powdered avocado seeds were extracted by

maceration using ethanol as the solvent for three cycles (24 h/cycle). The filtrate was separated from the residue using a rotary evaporator (230 mbar; 10 °C chiller; 50 °C heating bath) [20,21].

Metabolite profiling using UPLC-MS/MS

The avocado seed extract was dissolved in methanol and analyzed using UPLC-MS/MS (ACQUITY UPLC I-Class System coupled with a Xevo G2-XS Q-Tof Mass Spectrometer, Waters, USA). Chromatographic separation was achieved using gradient elution with water and acetonitrile, both containing 0.1% formic acid. Mass spectral data were acquired and processed using MassLynx software (version 4.1). Metabolites were tentatively identified by comparing their m/z values and spectral data with those available in the ChemSpider and PubChem databases [22,23].

Preparation of nanoemulsion

The nanoemulsion containing avocado seed extract was prepared by initially forming an oil phase consisting of avocado seed oil (ASO), Cremophor® RH40 (BASF, Ludwigshafen, Germany) as the surfactant, and PEG 400 (Brataco, Jakarta, Indonesia) as the co-surfactant. The nanoemulsion formulations were developed by varying the proportions of avocado seed oil (ASO), Cremophor® RH40, and PEG 400 presented in Table 1.

Table 1. Formula of nanoemulsion

Formula	ASO (ratio)	Cremophor® RH40 (ratio)	PEG 400 (ratio)
F1	1	8	1
F2	1	7	2
F3	1	6	3
F4	2	7	1
F5	3	6	1

Various concentrations of avocado seed extract, ranging from 0.1% to 5.0%, were incorporated into the optimum nanoemulsion oil phase formula. The extract, oil, surfactant, and co-

surfactant were mixed using a magnetic stirrer at 500 rpm for 2 h. Subsequently, the mixture was sonicated for 1 h using a bath sonicator [24]. Deionized water was then added to the oil phase at a 1:3 ratio, and the mixture was stirred continuously until a clear nanoemulsion formed. The resulting nanoemulsion was characterized for visual appearance and droplet size to determine the optimum formulation.

Physicochemical properties of nanoemulsion

The physical characteristics of the nanoemulsion, including particle size, polydispersity index (PDI), percent transmittance, viscosity, and morphology, were systematically evaluated. Particle size and PDI were determined using a particle size analyzer (Delsa™ Nano, Beckman Coulter, Brea, CA) at 25 °C, following a previously reported method [25]. The optical clarity of the nanoemulsion was assessed by measuring the percent transmittance (%T) at 650 nm using a UV–Visible spectrophotometer, with distilled water as the blank. The nanoemulsion's viscosity was measured using a Brookfield viscometer at 25 °C with a shear rate of 6 rpm. Morphological characterization of the nanoemulsion droplets was conducted using a transmission electron microscope (JEOL JEM1400, Tokyo, Japan) according to a previously established procedure, with observations performed at magnifications of 30,000× and 120,000× [26].

FT-IR characterization of nanoemulsion

Fourier transform infrared (FT-IR) spectroscopy was done at room temperature using Shimadzu IR (Shimadzu, UK). FT-IR analysis was carried out for pure avocado seed extract, pure avocado seed oil, pure chremophore RH40, pure PEG 400, nanoemulsion without extract, and nanoemulsion-avocado seed extract. The spectral range was 400-4000 cm^{-1} [27].

Stability of Nanoemulsion

The stability of the nanoemulsion was evaluated using a freeze–thaw method over three cycles: storage at −20 °C for 48 h, followed by storage at 25 °C for 48 h. The parameters observed included phase separation, particle size, polydispersity index, and pH value [28].

Preparation of Bacterial Nanocellulose

Bacteria were cultivated in Hestrin-Schramm medium supplemented with 10% G*luconacetobacter xylinus* InaCCB407.[29] The culture was stirred and incubated for 20 hours in a shaker [30]. The result was transferred to 100% Nira Sap as a new substrate. Bacterial Nanocellulose was cultivated in a new medium using the static culture fermentation method. The Bacterial Nanocellulose purification method was conducted using 1 M NaOH and 1% NaOCl for 1 h, respectively.

Preparation of BNC+NE-E serum

Avocado seed extract nanoemulsion serum was formulated by mixing BNC into the nanoemulsion. After other raw materials were added to the nanoemulsion and dissolved by mixing, BNC was added, stirred at 300 rpm and 75 °C for 15 minutes using a magnetic stirrer. The nanoemulsion was then transferred to a mechanical mixer and stirred for an additional 15 minutes at 700 rpm [31].

In-Vitro Skin Permeation Study

The diffusion study was conducted using Franz diffusion cells. The receptor compartment was filled with phosphate-buffered saline (PBS, pH 7.4) to a total volume of 15 mL. Approximately 500 mg of the sample formulation was uniformly applied onto a cellophane membrane. The temperature was maintained at 37 ± 1 °C, and the stirring speed was set at 120 rpm. Aliquots were withdrawn from the receptor compartment at predetermined time intervals of 5, 10, 15, 30, 45, 60, 90, and 120 min. At each sampling point, 3 mL of the receptor medium was withdrawn and immediately replaced with an equal volume of fresh PBS to maintain sink

conditions. The samples were analyzed using a UV–Visible spectrophotometer at 450 nm. The cumulative amount of flavonoids permeated per unit area was calculated using the regression equation derived from the calibration curve [28,32].

In-vivo Evaluation of Anti-Aging Effect

Animals

Male Wistar rats (*Rattus norvegicus*) were acclimatized for 7 days before the experiment to allow adaptation to the laboratory environment. The rats were housed in plastic cages with wire partitions and bedding made of rice husk. Each cage contained four rats. All animals were provided with ad libitum access to food and water under standard environmental conditions, including controlled room temperature and a 12 h light/12 h dark cycle [33]. The animals were randomly divided into six groups: normal control, model control, positive control, and three treatment groups receiving the test formulations [34]. The minimum sample size was determined using Federer's formula [35]. Based on this calculation, each group consisted of four rats.

UVB-Induced Photoaging Model in Rats

In this study, the dorsal surface of the rat skin was shaved with a razor over an area of 5 × 5 cm^2, leaving the area hairless during the experimental period. The frequency of exposure was determined three times a week for four weeks. Radiation exposure was performed using a Philips NB UVB lamp (model PL-S 9W/01-2P) with a wavelength of 305-311 nm. The exposure doses were 50 mJ/cm^2 in the first and second weeks, 70 mJ/cm^2 in the third week, and 80 mJ/cm^2 in the fourth week, for a total of 840 mJ/cm^2. Rats were sacrificed using cervical dislocation, and skin tissue was collected for histological analysis. Wrinkle formation was evaluated and quantified using ImageJ 1.53e [36].

Photoaging evaluation of dorsal skin

At the end of the experimental period, the UV-induced dorsal skin of each rat, which was under anesthesia, was photographed; the grade of photodamage was determined by referring to the grading scale described in Table 2. The skin photoaging level ranged from 0 (normal skin) to 6 (severely damaged skin).

Table 2. Grading scale for evaluation of photoaging

Grade	Evaluation Criteria
0	Smoothness without any wrinkles; fine striations running the length of the body
1	Fine striations
2	A few shallow wrinkles; disappearance of all fine striations
3	Shallow wrinkles across the dorsal skin
4	Deep and coarse wrinkles with laxity
5	Increased deep wrinkles
6	Surface accompanied by severe wrinkles; development of lesions

Evaluation of skin elasticity using the pinch test

A pinch test was performed on a rat using the method of Hou [34]. Briefly, after the end of the experimental period, the midline of the dorsal skin of the anesthetized rat was grasped with the fingers as much as possible (without lifting the animal into the air), the pinch was released, and the skin recovery time was immediately calculated.

Skin hydration measurement

At the end of the experimental period, the rats were sacrificed by cervical dislocation. The exposed skin on the back of the depilated rats was removed, about 0.2 g. The fatty tissue in the dermis was removed, and the wet weight of the skin was measured. Place the skin in an oven and dry to constant weight at 70° C, then weigh the dry skin. The skin hydration was calculated using the following equation: [34]

$$Skin\ hydration\ (\%) = \frac{M\ (wet\ weight - dry\ weight)}{M\ (wet\ weight)} \times 100\%$$

Analysis of collagen density

Observation was conducted microscopically on Masson's trichrome-stained slides using an Olympus® viewer at 12x20 magnification. Collagen density was measured in the dermal layer of the skin. Measurement of collagen area was done through software (ImageJ®) and displayed as a percentage. Measurements were taken in 4 fields of view, and the average value was calculated [34].

Statistical analysis

All data are presented as mean ± standard deviation (SD). ANOVA and Tukey's post hoc multiple comparison using Minitab were used for statistical analysis, and a p-value of <0,05 was considered statistically significant.

Results

Metabolite profiling using UPLC-MS/MS

The metabolite profile of the avocado seed extract was characterized based on chromatographic and mass-spectral data obtained during analysis. Several compounds were tentatively identified based on their retention times, molecular masses, and predicted molecular formulas (Table 3).

Table 3. Compounds identified in avocado seed extract by LC–ESI–MS analysis.

Peak	Retention Time (minute)	Mass	Formula	Compound
1	3.81	578.520	$C_{30}H_{26}O_{12}$	Procyanidin
2	5.72	222.194	$C_{11}H_{10}O_5$	Isofraxidin
3	6.66	205.210	$C_{11}H_{11}NO_3$	5-Methoxyindoleacetate
4	9.39	266.419	$C_{17}H_{30}O_2$	Heptadecadienoic acid
5	11.25	303.438	$C_{16}H_{33}NO_4$	dodecanoic acid (2-hydroxy-1,1-Bis-hydroxymethyl-ethyl) amide
6	11.32	266.419	$C_{17}H_{30}O_2$	Heptadecadienoic acid
7	17.45	954.200	$C_{47}H_{83}N_7O_{13}$	18-[2-[2-[2-[2-[2-[2-[[(5S)-5-Carboxy-5-[[3-[3-(1H-imidazol-5-yl)propanoylamino]-3-methyl-2-oxobutyl]amino]pentyl]amino]-2-oxoethoxy]ethoxy]ethylamino]-2-oxoethoxy]ethoxy]ethylamino]-18-oxooctadecanoic acid

Formulation and characterization of nanoemulsion

The characteristics of various nanoemulsion formulations are presented in Table 4 and Fig **1**.

Table 4. Characteristics of nanoemulsions with varying oil–surfactant–co-surfactant compositions

Formula	Visual appearance	Globule size (nm)	PI	%Transmittance	pH
FI	Clear	259.4±3.26	0.584±0.069	99.62±0.13	4.67±0.01
FII	Clear	22.8±0.40	0.224±0.017	99.62±0.27	4.81±0.00
FIII	Clear	20.3±0.10	0.109±0.029	99.62±0.27	4.92±0.01
FIV	Cloudy	3524.9	0.813	75.30±2.21	4.40±0.02
FV	Cloudy	3301.7	0.703	9.36±5.76	4.20±0.02

Fig 1. Visual appearance of nanoemulsion formula FI to FV (left to right).

Based on the data presented in Table 4, nanoemulsions (NEs) were formed spontaneously upon water addition in formulations FI–FIII, as evidenced by their clear and transparent appearance. In contrast, formulations FIV and FV failed to form nanoemulsions, as indicated by their cloudy visual appearance. All clear formulations (FI–FIII) demonstrated high transmittance values of approximately 99.6%, while cloudy formulations FIV and FV showed substantially lower transmittance. FIII showed the most favorable nanoscale characteristics, with a mean globule size of 20.3 ± 0.10 nm, a polydispersity index (PDI) of 0.109 ± 0.029, and a transmittance value of 99.62 ± 0.27%. The pH values of all formulations ranged from 4.20 to 4.92.

Optimization of avocado seed extract loading in the nanoemulsion system was conducted using FIII as the optimized oil nanoemulsion formulation. The concentration of extract incorporated and the loading capacity were evaluated based on several parameters summarized in Table 5 and Fig **2**.

Table 5. Characteristics of avocado seed extract nanoemulsion.

Formula	Avocado seed extract in 10 g oil phase (mg)	Visual appearance	Globule size (nm)	Polydispersity Index	%Transmittance	pH
NE 0	-	Clear	20.3±0.10	0.109±0.029	99.62±0.27	4.92±0.01
NE I	10	Clear	20.7±0.10	0.019±0.017	99.77±0.00	4.93±0.04
NE II	100	Clear	20.2±0.65	0.051±0.062	99.77±0.00	4.94±0.03
NE III	250	Clear	22.4±0.31	0.095±0.080	98.93±0.26	4.87±0.01
NE IV	500	Clear	23.7±1.01	0.196±0.045	98.55±0.47	4.79±0.01

Fig 2. Visual appearance of avocado seed extract nanoemulsion formula NE 0 to NE IV (left to right).

The incorporation of avocado seed extract up to 500 mg did not result in any significant changes in the nanoemulsion's visual appearance, globule size, PDI, transmittance values, or pH. Increasing the concentration of avocado seed extract led to a gradual colour change in the formulation from colourless to dark brown, as shown in Fig **2**. Therefore, it can be concluded that the solubility of avocado seed extract was relatively high, reaching up to 5%, and was evenly distributed throughout the external phase of the NE system without compromising the formulation's clarity. The polydispersity index (PDI) values of all formulations were less than 0.3, indicating that the formed globules were relatively uniform in size.

Morphology of Nanoemulsion

The morphology of the nanoemulsion was characterized using TEM, as shown in Fig 3. The globules of the avocado seed extract nanoemulsion appeared spherical with a homogeneous size distribution. These results indicate that the nanoemulsion remained stable and did not aggregate.

Fig 3. Morphology of extract avocado seed nanoemulsion (NE EAS) observed using TEM. (a) NE EAS at 30.0k× magnification with a scale bar of 200 nm; (b) NE EAS at 120k× magnification with a scale bar of 50 nm.

FT-IR characterization of nanoemulsion

Analysis of FT-IR confirmed the successful incorporation of avocado seed oil (ASO), Cremophor RH-40 and PEG-400 into the nanoemulsion system, as it reflected a combination of their functional groups (
Fig **4**). Avocado seed oil (ASO) showed a prominent ester carbonyl band at approximately 1740 cm^{-1} and aliphatic C-H stretching vibrations at 2920 and 2850 cm^{-1}, confirming its triglyceride structure. Cremophor RH-40 and PEG-400 displayed dominant C-O-C stretching bands around 1100 cm^{-1}, characteristic of polyoxyethylene-based surfactants. The FT-IR spectrum of EAS exhibited a broad absorption band around 3400 cm^{-1} attributed to O-H stretching of phenolic compounds, along with characteristic aromatic C=C vibrations at 1600-1630 cm^{-1} and C-O stretching bands in the 1240-1100 cm^{-1} region. The FT-IR spectrum of EAS-loaded nanoemulsion (NE-EAS) retained all major characteristic absorption bands of EAS and ASO without the appearance of significant band shifts. FT-IR analysis of the EAS-loaded nanoemulsion (NE-EAS) retained all major characteristic absorption bands of the extract and oil phase without the appearance of new peaks or significant band shifts, indicating

the absence of chemical interactions between EAS and the excipients. These findings suggest that the extract was physically encapsulated within the nanoemulsion matrix, preserving its chemical integrity.

Fig 4. FT-IR characterization of nanoemulsion.

Stability of Nanoemulsion

The physical stability of the nanoemulsion was evaluated using a freeze–thaw method by monitoring changes in globule size, polydispersity index (PDI), and pH (Fig 5). All formulations exhibited relatively stable globule sizes, with a slight increase throughout the freeze–thaw cycles. Among all formulations, FIII demonstrated the most stable in globule size across cycles, indicating superior physical stability compared with FI and FII. The PDI values of all formulations varied within the ranges of 0.193–0.526 for FI, 0.344–0.663 for FII, and 0.326–0.397 for FIII. Consistent with the globule size results, FIII exhibited lower PDI values, reflecting a more homogeneous particle size distribution. The pH values of all formulations remained relatively constant during the freeze–thaw testing, with ranges of 4.50–4.67 for FI, 4.64–4.81 for FII, and 4.78–4.92 for FIII. These results indicate that the pH of the nanoemulsion formulations remained stable and within a skin-compatible range under thermal stress conditions.

Fig 5. Freeze-thaw stability for 3 cycles. (a) globule size; (b) polydispersity index; and (c) pH.

Formulation and characterization of BNC+NE-E serum

As shown in Table 6 and Fig 6, three serum formulations with different gelling systems were evaluated for physical appearance, spreadability, viscosity, and pH. All formulations (Ser1–

Ser3) exhibited a brown, transparent gel appearance, indicating good homogeneity and absence of phase separation or precipitation. Ser1 showed the highest spreadability (5.300 ± 0.200 cm), indicating a softer gel structure, while Ser2 and Ser3 exhibited lower spreadability values of 4.801 ± 0.505 cm and 4.659 ± 0.140 cm, respectively. Viscosity measurements demonstrated notable differences among formulations. Ser1 showed the lowest viscosity (352.62 mPa·s), followed by Ser2 (388.24 mPa·s), while Ser3 exhibited the highest viscosity (510.81 mPa·s). These results reflect the influence of the gelling agent type on the rheological properties of the serum formulations. The pH values of all formulations ranged from 5.12 to 5.41. No extreme pH deviations were observed, indicating that all formulations are suitable for topical application.

Table 6. Characteristics of NE-E Serum

Formula	Carbomer 940	HPMC	BNC	Gliserin	Propilenglikol	Physical Appearance	Spreadability (cm)	Viscosity (mPa.s)	pH
Ser1	-	-	+	-	-	Brown Transparent gel	5.300±0.200	352.62	5.41±0.05
Ser2	-	5	-	5	15	Brown Transparent gel	4.801±0.505	388.24	5.19±0.02
Ser3	1	-	-	5	15	Brown Transparent gel	4.659±0.140	510.81	5.12±0.01

Fig 6. Visual appearance of serum avocado seed extract formula Ser1 to Ser3 (left to right).

FT-IR characterization of serum BNC

The FT-IR spectra illustrate a comparison of BNC (black line), NE (blue line), and BNC+NE (red line) (Fig 7). The observed shifts in absorption bands indicate potential chemical interactions between the avocado seed extract nanoemulsion and the BNC matrix.

Fig 7. FT-IR spectrum of BNC Serum

The NE spectrum exhibited distinct absorption bands at approximately 2924, 2856, 1955, 1737, 950, 885, 842 cm^{-1}, which are attributed to the stretching vibrations of C-H and C=O stretching. In the BNC+NE spectrum, these bands were still observed, although with altered intensities, indicating the successful incorporation of the oil phase into the BNC-based system. The absorption band at 1737 cm^{-1}, which indicates the C=O group derived from avocado seed oil, was absent in the pure BNC spectrum, confirming that its presence serves as a marker for the successful incorporation of the extract-containing nanoemulsion into the system. A band observed at approximately 1633 cm^{-1} is associated with aromatic C=C stretching vibrations or bound water vibrations in BNC. The shift of this band in the BNC+NE spectrum suggests interactions between polyphenolic components of the avocado seed extract and the BNC structure.

In-Vitro Skin Permeation Study

Permeation studies were conducted to compare the cumulative penetration ability of two serum formulations, namely BNC combined with a conventional emulsion (BNC+E) and BNC combined with a nanoemulsion (BNC+NE), both containing avocado seed extract (Fig 8).

Fig 8. In-vitro diffusion profile of BNC Serum.

As shown in the diffusion profile, the BNC+NE formulation consistently exhibited a higher penetration rate and cumulative penetration than BNC+E throughout the observation period. At 10 minutes, the cumulative penetration of BNC+NE reached 63.94 ± 0.60 μg/cm², whereas BNC+E showed 47.47 ± 0.71 μg/cm². This difference became more pronounced at 30 minutes, where BNC+NE reached 148.69 ± 0.54 μg/cm², compared to 52.34 ± 0.43 μg/cm² for BNC+E. After 120 minutes, the cumulative penetration of BNC+NE further increased to 198.28 ± 0.54 μg/cm², while BNC+E reached 121.35 ± 0.54 μg/cm². These findings demonstrate that incorporating avocado seed extract into a nanoemulsion-based system enhances its penetration performance compared with a conventional emulsion system, suggesting the advantage of nanoscale delivery for improving diffusion.

In-Vivo Evaluation of Anti-aging

Macroscopic evaluation of dorsal skins

Based on the

Fig **9**, UVB irradiation for 4 weeks significantly affected rat skin, as indicated by an increase in the visual wrinkle score in the model control group from 1 to 4.5 ± 0.6. The results also demonstrate that treatment with the tested formulations affected one anti-aging parameter, namely wrinkle formation. Macroscopic observation revealed that all treatment groups prevented wrinkle formation on the dorsal skin of rats after 4 weeks of UVB exposure.

Fig 9. Macroscopic evaluation of dorsal skins. (a) Physical appearances of UV-irradiated rats with various treatments at the last week; (b) Results of the visual score of different experimental groups at the last week. All data shown are mean±SD of 4 rats; p<0,05.

Evaluation of skin elasticity using the pinch test

Skin elasticity was assessed using the pinch test by measuring the recovery time required for the dorsal skin to return to its original position. The normal control group maintained relatively stable recovery times, indicating normal skin elasticity. In contrast, model control group exhibited a progression in recovery time throughout observation period, indicating reduced skin elasticity as a result of UVB exposure (Fig 10). Compared with model control, rats in BNC+NE group showed significant reductions in recovery time compared to model control group, showed that BNC+NE could promote the skin elasticity.

Fig 10. Evaluation of skin elasticity based on pinch test recovery time in UVB-induced photoaging rats treated with BNC-based nanoemulsion serum. All data shown are mean±SD of 4 rats.

Skin hydration measurement

Fig 11. Evaluation of skin hydration of UV irradiation on rats. All data shown are the mean±SD of 4 rats. One-way ANOVA ($p<0,05$) with *post hoc* Tukey's multiple comparison test was used for statistical analysis.

The results showed the percentage of in vivo skin hydration measurement among the treatment groups (Fig 11). Compared to normal group, model group exhibited decreased skin hydration with 31.29% and 27.91%, respectively. Positive control All BNC+NE-E treated groups showed increased skin hydration compared to the model group, with 29.09%, 31.41%, and 31.97% of skin hydration level alongside an increase of extract concentration. However, each group showed no statistically significant in skin hydration.

Analysis of collagen density

Fig 12. Evaluation of collagen density. (a) Rat skin tissue observed by Masson's trichrome staining. (b) Percentage of collagen density. All data shown are mean±SD of 4 rats; *p<0,05 and ***p<0.0001. One-way ANOVA with *post hoc* Tukey's multiple comparison test was used for statistical analysis.

The histological study of the normal control group exhibited a relatively loose dermal structure with abundant collagen fibers (

Fig **12**A). Following UVB exposure, the model control group showed marked histological alterations with significant reduction in collagen density. In contrast, the positive control group and all BNC-based nanoemulsion extract (BNC+NE-E) treatment groups demonstrated improved collagen density compared to the model control. An increase in collagen intensity and a progressively denser dermal structure were observed with increasing concentrations of avocado seed extract. Tissue sections from the positive control group and the BNC-based nanoemulsion treatment group at 5% concentration displayed a broader distribution of collagen fibers compared with the other treatment groups.

Collagen density was assessed by green color intensity and quantified as a percentage (%) using ImageJ software, as shown in

Fig **12**B. Significant differences in collagen density were observed among the treatment groups, with the model control group showing the lowest collagen density. The BNC+NE-E 1.25% group exhibited lower collagen density compared to the normal control, whereas the BNC+NE-E 2.5% and 5% groups showed higher collagen density values than the normal control group. Statistical analysis revealed a significant difference ($p < 0.0001$) between the model control group and all treatment groups. Each BNC+NE-E formulation exhibited significantly different collagen density values across concentrations. The BNC+NE-E 2.5%

group showed no significant difference compared with the normal control, suggesting that formulation restored UVB-exposed skin to normal conditions. In contrast, the BNC+NE-E 5% formulation demonstrated a significantly higher collagen density than the normal control, indicating not only recovery from UVB-induced damage but also an enhancement of dermal structural integrity.

Discussion

The identified compounds belonged to various chemical classes, including phenolic compounds, fatty acid derivatives, amino acid derivatives, alkaloid-related structures, and lipid-related molecules. Among the detected compounds, procyanidin which is the polymer of catechin and epictachin was identified as a prominent polyphenolic compound [37]. It has been widely reported to exhibit strong antioxidant properties and protective effects against oxidative stress-induced skin damage [38–40]. Several fatty acid derivatives, i.e heptadecadienoic acid and hexadecanedioic acid were also observed. The presence of fatty acid derivatives may support the skin barrier and enhance hydration, both of which are essential factors in preventing premature aging [41]. These findings suggest that the observed biological effects of the formulation may arise from the combined action of multiple bioactive constituents rather than a single compound.

The nanoemulsion exhibited a clear, transparent appearance, attributed to its globule diameter being significantly smaller than the wavelength of visible light, resulting in minimal light scattering [42]. Avocado seed oil is inherently lipophilic, with an HLB value of approximately 7, was successfully formulated using Cremophor® RH40, a non-ionic surfactant with an HLB value of 14–16, and PEG 400 as a co-surfactant. This surfactant system effectively reduced interfacial tension and promoted spontaneous nanoemulsion formation, resulting in a physically stable system [43–46].

The nanoscale size and spherical morphology increase the surface area available for skin contact, thereby facilitating enhanced interaction with the membrane and promoting diffusion of active compounds. This mechanistic interpretation is in agreement with the diffusion study outcomes and the observed in vivo anti-aging performance. Similar structural characteristics have also been reported for Cremophor® RH40–PEG 400–based nanoemulsion system, supporting the stability and effectiveness of this formulation approach [47].

Particle size distribution and morphological analyses further confirmed the uniformity and stability of the nanoemulsion system. The low PDI values obtained indicate a relatively homogeneous dispersion and good physical stability [48]. These results correlated well with TEM observations, which showed uniformly distributed, spherical-to-oval nanoemulsion droplets with well-defined interfacial layers, indicating effective oil encapsulation and minimal aggregation [49]. Consistent with these findings, the freeze–thaw stability test showed that globule size and PDI remained relatively stable up to the last cycle, confirming the robustness of the nanoemulsion system.

The incorporation of the nanoemulsion into the BNC matrix produced a transparent hydrogel, despite the presence of humectants, attributable to the nanofibrillar BNC network forming a three-dimensional structure with nanoscale pores that minimized light scattering [50]. Increased viscosity was associated with reduced spreadability, consistent with rheological behavior. However, the formulations maintained adequate spreadability to ensure uniform skin coverage and a lightweight sensory profile. Furthermore, the pH values of all formulations remained within the physiological skin range, indicating suitability for prolonged topical application [51].

FT-IR analysis of the BNC-based nanoemulgel revealed slight changes in the absorption bands corresponding to –OH, C-H, C=O, and C-O-C groups, suggesting physicochemical interactions such as hydrogen bonding and possible hydrophobic interactions between the bioactive

compounds of the extract (such as phenolics and triglycerides) and the hydroxyl groups of BNC [52]. The absence of new absorption bands confirms that these interactions were predominantly physical rather than covalent. Such interactions indicate successful incorporation of the nanoemulsion into the BNC matrix and support its potential application as a stable topical delivery system for pharmaceutical or cosmetic formulations [53].

The enhanced diffusion behavior observed with the BNC+NE formulation can be attributed to the small, homogeneous droplets of the nanoemulsion, which facilitate penetration of active compounds across the diffusion membrane. Nanoemulsions with particle size below 80 nm can efficiently fill the whole hair follicle canals, reach underneath the dermal surfaces, and diffuse into the surrounding dermal tissues by temporarily alter lipid organization within membrane, thereby increasing their availability [54,55]. In contrast, the conventional emulsion system (BNC+E), characterized by larger droplet sizes, exhibited slower diffusion behavior and lower cumulative penetration. Overall, these findings suggest that incorporation of a nanoemulsion system enhances the diffusion performance of avocado seed extract in BNC-based formulations [56,57].

Furthermore, the BNC matrix itself may prolong the residence time of nanoemulsion droplets on the membrane surface, enabling sustained diffusion. Similar reservoir-like behavior and prolonged release effects have been reported for nanocellulose-based hydrogel systems in topical delivery applications [58]. Although the diffusion study was conducted using a synthetic membrane, it provides a reliable preliminary assessment of comparative penetration performance between formulations [59].

The in vivo findings support the enhanced delivery performance observed in the diffusion study. UVB irradiation successfully induced photoaging characteristics, as evidenced by increased wrinkle formation, reduced skin hydration, and decreased collagen density in the model control group, consistent with previous reports on UVB-induced skin photoaging and

barrier disruption [60,61]. In contrast, treatment with the BNC-based nanoemulsion containing avocado seed extract reduced wrinkle formation and improved overall skin appearance, indicating a protective effect against UVB-induced skin damage.

Treatment with the BNC-based nanoemulsion serum resulted in a gradual decrease in recovery time across all concentrations, suggesting improved skin elasticity. At the end of the treatment period, the 5% formulation exhibited the shortest recovery time among all treated groups, approaching or surpassing the normal control group. These findings suggest that the BNC-based nanoemulsion containing avocado seed extract effectively improved skin elasticity in a concentration-dependent manner and mitigated UVB-induced loss of skin elasticity. Skin elasticity assessment using the pinch test further demonstrated the formulation's protective effect with reduced recovery time observed particularly in the 2.5% and 5% groups, reflecting improved skin elasticity. Enhanced penetration by the nanoemulsion system and prolonged contact time supported by BNC matrix may contribute to this effect [62–64].

Skin hydration measurements showed a decreasing trend in the model control group compared with the normal control, reflecting impairment of the skin barrier following UVB exposure. Treatment with the BNC-based nanoemulsion demonstrated a concentration-dependent increase in hydration values, with the 2.5% and 5% formulations approaching normal skin conditions. Although statistical differences were not significant, the observed trend suggests a protective effect against UVB-induced dehydration, potentially associated with the high water-retention capacity of the BNC matrix and enhanced delivery of bioactive compounds [65–67].

Histological evaluation demonstrated that UVB exposure markedly reduced dermal collagen density, whereas treatment with the BNC-based nanoemulsion significantly restored collagen levels compared with the model control. UV-induced reactive oxygen species (ROS) are known to upregulate matrix metalloproteinases (MMPs), resulting in accelerated collagen degradation [68]. The 2.5% formulation restored collagen density, while the 5% formulation produced the

highest collagen deposition. Collagen improvement is attributed to antioxidant which scavenge ROS, reduce oxidative stress, inhibit MMP-1, MMP-3, and MMP-9, and protect dermal fibroblasts, thereby promoting type I collagen synthesis [69]. Quantitative ImageJ analysis was consistent with the histological observations, confirming the significant influence of BNC-based nanoemulsion treatment on collagen density.

Beyond the contribution of the bioactive compounds, the enhanced in vivo efficacy may also be attributed to improved skin penetration facilitated by the nanoscale globule size and low PDI of the nanoemulsion system. Increased penetration enhances bioavailability, thereby amplifying in vivo therapeutic outcomes [59]. Similar findings have been reported in previous studies, in which nanoemulsion-based formulations significantly improved topical efficacy by enhancing penetration compared with conventional systems [64]. Bacterial nanocellulose (BNC) serves as an effective delivery matrix, enhancing formulation retention and prolonging skin contact [19]. Its synergistic integration with nanoemulsion technology improves anti-aging efficacy by enhancing skin hydration and dermal penetration.

Conclusion

This study demonstrated that a BNC-based nanoemulsion serum formulation loaded with *Persea americana* seed extract provides a stable, biocompatible, and effective anti-aging. The nanoemulsion precursor exhibited favorable physicochemical properties, preserved key bioactive compounds, and remained stable under stress thermal conditions. The nanoemulsion was successfully adsorbed into the BNC matrix, demonstrating the desired physicochemical characteristics and enhancing in vitro diffusion performance by more than 1.5-fold compared with the BNC loaded with a conventional emulsion. Notably, the BNC-based nanoemulsion serum demonstrated superior collagen preservation compared to the model control, indicating its potential as an effective natural alternative for anti-aging applications. Overall, the findings

support the BNC-based nanoemulsion loaded with *Persea americana* seed extract as a promising plant by-product and alternative strategy for anti-aging and improving skin integrity.

Acknowledgement

This work was supported by Beasiswa Pendidikan Indonesia (BPI) (Grant No. 202101121555), Center for Higher Education Funding and Assessment (PPAPT) under the Ministry of Higher Education, Science, and Technology, Indonesia and the Indonesia Endowment Funds for Education (LPDP) under the Ministry of Finance, Indonesia.

References

[1] Atikah Risyad, Resi Levi Permadani, Siswarni MZ. EKSTRAKSI MINYAK DARI BIJI ALPUKAT (Persea Americana Mill) MENGGUNAKAN PELARUT N-HEPTANA. Jurnal Teknik Kimia USU 2016;5:34–9. https://doi.org/10.32734/jtk.v5i1.1522.

[2] Segovia FJ, Hidalgo GI, Villasante J, Ramis X, Almajano MP. Avocado Seed: A Comparative Study of Antioxidant Content and Capacity in Protecting Oil Models from Oxidation. Molecules 2018;23:2421. https://doi.org/10.3390/molecules23102421.

[3] Laksmiani NPL, Sanjaya IKN, Leliqia NPE. The activity of avocado (Persea americana Mill.) seed extract containing catechin as a skin lightening agent. J Pharm Pharmacogn Res 2020;8:449–56. https://doi.org/10.56499/jppres20.836_8.5.449.

[4] Segovia FJ, Corral-Pérez JJ, Almajano MP. Avocado seed: Modeling extraction of bioactive compounds. Ind Crops Prod 2016;85:213–20. https://doi.org/10.1016/j.indcrop.2016.03.005.

[5] Masaki H. Role of antioxidants in the skin: Anti-aging effects. J Dermatol Sci 2010;58:85–90. https://doi.org/10.1016/j.jdermsci.2010.03.003.

[6] Castro GA, Coelho ALLR, Oliveira CA, Mahecha GAB, Oréfice RL, Ferreira LAM. Formation of ion pairing as an alternative to improve encapsulation and stability and to reduce skin irritation of retinoic acid loaded in solid lipid nanoparticles. Int J Pharm 2009;381:77–83. https://doi.org/10.1016/j.ijpharm.2009.07.025.

[7] Sahu G, Sahu S, Sharma H, Jha AK. A review of current and novel trends for anti-ageing formulation. International Journal of Pharmaceutical, Chemical and Biological Sciences 2014;4:118–25.

[8] Gupta PK, Pandit JK, Kumar A, Swaroop P, Gupta S. Novel nanoemulsion – high energy emulsification: preparation, evaluation and application. The Pharma Research 2010;3:117–38.

[9] Budiasih S, Masyitah I, Jiyauddin K, Kaleemullah M, Samer AD, Fadli AM, et al. Formulation and Characterization of Cosmetic Serum Containing Argan Oil as Moisturizing Agent. Proceedings of BROMO Conference, SCITEPRESS - Science and Technology Publications; 2018, p. 297–304. https://doi.org/10.5220/0008361702970304.

[10] Kar M, Chourasiya Y, Maheshwari R, Tekade RK. Current Developments in Excipient Science. Basic Fundamentals of Drug Delivery, Elsevier; 2019, p. 29–83. https://doi.org/10.1016/B978-0-12-817909-3.00002-9.

[11] Putri Rahmani SI, Zulkarnain AK. Optimization of HPMC and Na-CMC as Gelling Agents on Physical Properties and Stability in Sunflower Seed Oil Gel Formulation. Journal of Food and Pharmaceutical Sciences 2023;11:812–9.

[12] Ozon EA, Burloiu AM, Musuc AM, Manda G, Anuta V, Dinu-Pîrvu CE, et al. Cellulose-Derived Gels for Topical Delivery: HPMC as a Functional Matrix for Porphyrinic Photosensitizers. Gels 2025;11:824. https://doi.org/10.3390/gels11100824.

[13] Utami N, Amin MS, Angelia PT, Paramesti NA. Formulation of Palmarosa (Cymbopogon martinii) Essential Oil Emulgel Using Sodium Carboxymethyl Cellulose (Na-CMC) as a Gelling Agent. Indonesian Journal of Global Health Research 2025;7.

[14] Meneses L, Jesus AR. Eutectogels: Recent Advances, Design Strategies, and Emerging Applications in Biotechnology. Gels 2025;11:1013. https://doi.org/10.3390/gels11121013.

[15] Khare S, Kumar S, Urmaliya P, Yadav S. Recent advancement and applications of green hydrogels: Revolutionizing biomedicine and environmental sustainability. Results Chem 2025;18:102857. https://doi.org/10.1016/j.rechem.2025.102857.

[16] Garcia KR, Beck RCR, Brandalise RN, dos Santos V, Koester LS. Nanocellulose, the Green Biopolymer Trending in Pharmaceuticals: A Patent Review. Pharmaceutics 2024;16:145. https://doi.org/10.3390/pharmaceutics16010145.

[17] Shahriari-Khalaji M, Zarkesh M, Nozhat Z. Application of Bacterial Nanocellulose in Cancer Drug Delivery: A Review. Curr Pharm Des 2021;27:3656–65. https://doi.org/10.2174/1381612827666210412150445.

[18] Pötzinger Y, Kralisch D, Fischer D. Bacterial Nanocellulose: The Future of Controlled Drug Delivery? Ther Deliv 2017;8:753–61. https://doi.org/10.4155/tde-2017-0059.

[19] Silva NHCS, Mota JP, Santos de Almeida T, Carvalho JPF, Silvestre AJD, Vilela C, et al. Topical Drug Delivery Systems Based on Bacterial Nanocellulose: Accelerated Stability Testing. Int J Mol Sci 2020;21:1262. https://doi.org/10.3390/ijms21041262.

[20] Sanjaya IKN, Giantari NKM, Widyastuti MD, Laksmiani NPL. Extraction of catechins from avocado seeds with various solvents using maceration method. Jurnal Kimia 2020:1. https://doi.org/10.24843/JCHEM.2020.v14.i01.p01.

[21] Ginting B, Maulana I, Yahya M, Saidi N, Murniana M, Hasballah K, et al. Antioxidant and antiproliferative activities of n-hexane extract and its fractions from Blumea balsamifera L. leaves. J Adv Pharm Technol Res 2022;13:216. https://doi.org/10.4103/japtr.japtr_105_22.

[22] Sahlan M, Devina A, Pratami DK, Situmorang H, Farida S, Munim A, et al. Anti-inflammatory activity of Tetragronula species from Indonesia. Saudi J Biol Sci 2019;26:1531–8. https://doi.org/10.1016/j.sjbs.2018.12.008.

[23] Mutiah R, Rachmawati E, Fitrianingsih AA, Zahiro SR. Metabolite profiling of anticancer compounds in Saussure lappa based on UPLC-QToFMS/MS. Pharmacy Education 2023;23:37–42. https://doi.org/10.46542/pe.2023.234.3742.

[24] Sari TA, Adi AC, Rachmawati H. Development of nanoemulgel containing nanobentonite-purified catfish oil for candidate of a wound healing dosage form. J Appl Pharm Sci 2024. https://doi.org/10.7324/JAPS.2024.166275.

[25] Adi AC, Christanto C, Rachmawati H, Adlia A. Vitamin E-based Folic Acid Nanoemulsion: Formulation and Physical Evaluation for Oral Administration. Pharm Nanotechnol 2019;7:304–13. https://doi.org/10.2174/2211738507666190717154040.

[26] Nabila N, Suada NK, Denis D, Yohan B, Adi AC, Veterini AS, et al. Antiviral Action of Curcumin Encapsulated in Nanoemulsion against Four Serotypes of Dengue Virus. Pharm Nanotechnol 2020;8:54–62. https://doi.org/10.2174/2211738507666191210163408.

[27] Fatmawati S, Istiqomah SM, Hasanah N, Ina Kewa Helan ME, Santoso M, Nugraheni ZV, et al. Physico-chemical characterization of natural nano calcium extracted from different fish bones in catfish (Clarias gariepinus) and snakehead fish (Channa striata). Case Studies in Chemical and Environmental Engineering 2025;11:101080. https://doi.org/10.1016/j.cscee.2024.101080.

[28] Sungpud C, Panpipat W, Chaijan M, Sae Yoon A. Techno-biofunctionality of mangostin extract-loaded virgin coconut oil nanoemulsion and nanoemulgel. PLoS One 2020;15:e0227979. https://doi.org/10.1371/journal.pone.0227979.

[29] Schneider C, Gordon ON, Edwards RL, Luis PB. Degradation of Curcumin: From Mechanism to Biological Implications. J Agric Food Chem 2015;63:7606–14. https://doi.org/10.1021/acs.jafc.5b00244.

[30] Asthary PB, Saepulloh S, Sanningtyas A, Pertiwi GA, Purwita CA, Septiningrum K. Optimization of bacterial nanocellulose production in agitation culture methods. JURNAL SELULOSA 2021;10:89. https://doi.org/10.25269/jsel.v10i02.295.

[31] Kucuk SD, Groso A, Collet G, Daniellou R, Caliskan UK. Effect of bacterial nanocellulose and plant-containing facial serum on hyperpigmentation in in-vitro conditions. Bioresources 2024;19:3208–33. https://doi.org/10.15376/biores.19.2.3208-3233.

[32] Sapra A, Mus S, Dwirandy D, Malluka R, Khairi N. Diffusion Study of Cream Formula of Lyophilized Ethanol Extract of Moringa oleifera L. Leaves. Jurnal Farmasi Galenika (Galenika Journal of Pharmacy) (e-Journal) 2021;7:131–42. https://doi.org/10.22487/j24428744.2021.v7.i2.15275.

[33] Matsui M, Tanaka K, Higashiguchi N, Okawa H, Yamada Y, Tanaka K, et al. Protective and therapeutic effects of fucoxanthin against sunburn caused by UV irradiation. J Pharmacol Sci 2016;132:55–64. https://doi.org/10.1016/j.jphs.2016.08.004.

[34] Hou X, Qiu X, Wang Y, Song S, Cong Y, Hao J. Application and Efficacy of Melatonin Elastic Liposomes in Photoaging Mice. Oxid Med Cell Longev 2022;2022. https://doi.org/10.1155/2022/7135125.

[35] Federer WT. Statistics and Society: Data Collection and Interpretation. 2nd ed. New York: Marcel Dekker, Inc.; 1991.

[36] Mayangsari E, Mustika A, Nurdiana N, Samad NA. Comparison of UVA vs UVB Photoaging Rat Models in Short-term Exposure. Medical Archives 2024;78:88–91. https://doi.org/10.5455/medarh.2024.78.88-91.

[37] Rahman A-., Choudhary MIqbal. Applications of NMR spectroscopy. Volume 2. Bentham Science Publishers Ltd.; 2015.

[38] Yang L, Xian D, Xiong X, Lai R, Song J, Zhong J. Proanthocyanidins against Oxidative Stress: From Molecular Mechanisms to Clinical Applications. Biomed Res Int 2018;2018:1–11. https://doi.org/10.1155/2018/8584136.

[39] Ye H, Sun J, He L, Ai C, Jin W, Abd El-Aty AM. Beneficial effects of proanthocyanidins on skin aging: a review. Front Nutr 2025;12. https://doi.org/10.3389/fnut.2025.1650328.

[40] Dasiman R, Nor NM, Eshak Z, Mutalip SSM, Suwandi NR, Bidin H. A Review of Procyanidin: Updates on Current Bioactivities and Potential Health Benefits. Biointerface Res Appl Chem 2021;12:5918–40. https://doi.org/10.33263/BRIAC125.59185940.

[41] Nambidi S, Sennie NS, Kondaveeti SB, Banerjee A, Pathak S, Duttaroy AK. A review of short-Chain fatty acids in gut and skin: Possible implications in skin aging. J Funct Foods 2025;133:107010. https://doi.org/10.1016/j.jff.2025.107010.

[42] Zhang Y, Gao J, Zheng H, Zhang R, Han Y. The preparation of 3,5-dihydroxy-4-isopropylstilbene nanoemulsion and in vitro release. Int J Nanomedicine 2011:649. https://doi.org/10.2147/IJN.S17242.

[43] Venkata Ramana Rao S, Shao J. Self-nanoemulsifying drug delivery systems (SNEDDS) for oral delivery of protein drugs. Int J Pharm 2008;362:2–9. https://doi.org/10.1016/j.ijpharm.2008.05.018.

[44] GAYATRI PA, . K, SAHLAN M, PRATAMI DK, WIDAYATI R. STABILITY OF ZOLEDRONATE GEL EMULSION IN VIRGIN COCONUT OIL. International Journal of Applied Pharmaceutics 2019:201–6. https://doi.org/10.22159/ijap.2019.v11s1.18204.

[45] Chang Y, McClements DJ. Optimization of Orange Oil Nanoemulsion Formation by Isothermal Low-Energy Methods: Influence of the Oil Phase, Surfactant, and Temperature. J Agric Food Chem 2014;62:2306–12. https://doi.org/10.1021/jf500160y.

[46] Attwood D. Microemulsions in Colloidal Drug Delivery Systems. In: Kreuter J, editor. Microemulsions in Colloidal Drug Delivery Systems, New York, NY, USA: Marcel Dekker Inc.; 1994.

[47] RACHMAWATI H. In Vitro Study on Antihypertensive and Antihypercholesterolemic Effects of Curcumin Nanoemulsion. Sci Pharm 2016;84:131–40. https://doi.org/10.3797/scipharm.ISP.2015.05.

[48] Ding Z, Jiang Y, Liu X. Nanoemulsions-Based Drug Delivery for Brain Tumors. Nanotechnology-Based Targeted Drug Delivery Systems for Brain Tumors, Elsevier; 2018, p. 327–58. https://doi.org/10.1016/B978-0-12-812218-1.00012-9.

[49] Soyuçok A, Kılıç B, Kılıç GB. Assessment of In Vitro Antioxidant Capacity of Ginseng Extract and Its Effect on Inhibiting Lipid Oxidation and Physicochemical Properties of Cooked Ground Beef During Refrigerated Storage. Food Technol Biotechnol 2024;62:140–9. https://doi.org/10.17113/ftb.62.02.24.8244.

[50] Geiss O, Bianchi I, Blazevic I, Bucher G, El-Hadri H, Fumagalli F, et al. Characterisation of Nanocellulose Types Using Complementary Techniques and Its Application to Detecting Bacterial Nanocellulose in Food Products. Nanomaterials 2025;15:1565. https://doi.org/10.3390/nano15201565.

[51] Lambers H, Piessens S, Bloem A, Pronk H, Finkel P. Natural skin surface pH is on average below 5, which is beneficial for its resident flora. Int J Cosmet Sci 2006;28:359–70. https://doi.org/10.1111/j.1467-2494.2006.00344.x.

[52] Azeez S, Shenbagaraman R. Fourier transform infrared spectroscopy in characterization of bionanocomposites. Characterization Techniques in Bionanocomposites, Elsevier; 2025, p. 209–27. https://doi.org/10.1016/B978-0-443-22067-8.00008-3.

[53] Palacios-Hinestroza H, López-Jaramillo MC, Martínez-Galán JP, Molina-Ramírez C, Sánchez-Osorno DM. Physical Properties of Bacterial Nanocellulose as an Encapsulant Material of Vitamin B12. Molecules 2025;30:4172. https://doi.org/10.3390/molecules30214172.

[54] Su R, Fan W, Yu Q, Dong X, Qi J, Zhu Q, et al. Size-dependent penetration of nanoemulsions into epidermis and hair follicles: implications for transdermal delivery and immunization. Oncotarget 2017;8:38214–26. https://doi.org/10.18632/oncotarget.17130.

[55] Islam MdR, Uddin S, Chowdhury MdR, Wakabayashi R, Moniruzzaman M, Goto M. Insulin Transdermal Delivery System for Diabetes Treatment Using a Biocompatible Ionic Liquid-Based Microemulsion. ACS Appl Mater Interfaces 2021;13:42461–72. https://doi.org/10.1021/acsami.1c11533.

[56] Almeida T, Moreira P, Sousa FJ, Pereira C, Silvestre AJD, Vilela C, et al. Bioactive Bacterial Nanocellulose Membranes Enriched with Eucalyptus globulus Labill. Leaves Aqueous Extract for Anti-Aging Skin Care Applications. Materials 2022;15:1982. https://doi.org/10.3390/ma15051982.

[57] Kupnik K, Brezovec N, Knez Ž, Leitgeb M, Primožič M. Bacterial nanocellulose biohybrid membranes and beads for potential cosmetics, food, and drug delivery applications. Cellulose 2026;33:1001–21. https://doi.org/10.1007/s10570-025-06907-y.

[58] Silva NHCS, Rodrigues AF, Almeida IF, Costa PC, Rosado C, Neto CP, et al. Bacterial cellulose membranes as transdermal delivery systems for diclofenac: In vitro dissolution and permeation studies. Carbohydr Polym 2014;106:264–9. https://doi.org/10.1016/j.carbpol.2014.02.014.

[59] Hmingthansanga V, Singh N, Banerjee S, Manickam S, Velayutham R, Natesan S. Improved Topical Drug Delivery: Role of Permeation Enhancers and Advanced Approaches. Pharmaceutics 2022;14:2818. https://doi.org/10.3390/pharmaceutics14122818.

[60] Choi YJ, Cho E-C, Lim S, Lee J, Bae J, Oh TK, et al. Oral Centella asiatica Extract Attenuates UVB-Induced Skin Photoaging via Antioxidant, Anti-Inflammatory, and Extracellular Matrix-Preserving Effects in Hairless Mice. Int J Mol Sci 2025;27:204. https://doi.org/10.3390/ijms27010204.

[61] Li Z, Jiang R, Wang M, Zhai L, Liu J, Xu X, et al. Ginsenosides repair UVB-induced skin barrier damage in BALB/c hairless mice and HaCaT keratinocytes. J Ginseng Res 2022;46:115–25. https://doi.org/10.1016/j.jgr.2021.05.001.

[62] Zimowska K, Filipovic V, Nikodinovic-Runic J, Simic J, Ilic-Tomic T, Zimowska M, et al. Modulating the Release Kinetics of Natural Product Actinomycin from Bacterial Nanocellulose Films and Their Antimicrobial Activity. Bioengineering 2024;11:847. https://doi.org/10.3390/bioengineering11080847.

[63] Moraru A, Dima Ștefan-O, Tritean N, Oprița E-I, Prelipcean A-M, Trică B, et al. Bioactive-Loaded Hydrogels Based on Bacterial Nanocellulose, Chitosan, and Poloxamer for Rebalancing Vaginal Microbiota. Pharmaceuticals 2023;16:1671. https://doi.org/10.3390/ph16121671.

[64] Deveci E. Nanoemulsions in cosmetics: Enhancing efficacy and stability. Journal of Dermatologic Science and Cosmetic Technology 2025;2:100107. https://doi.org/10.1016/j.jdsct.2025.100107.

[65] Horue M, Silva JM, Berti IR, Brandão LR, Barud H da S, Castro GR. Bacterial Cellulose-Based Materials as Dressings for Wound Healing. Pharmaceutics 2023;15:424. https://doi.org/10.3390/pharmaceutics15020424.

[66] Costa L, Carvalho AF, Fernandes AJS, Campos T, Dourado N, Costa FM, et al. Bacterial nanocellulose as a simple and tailorable platform for controlled drug release. Int J Pharm 2024;663:124560. https://doi.org/10.1016/j.ijpharm.2024.124560.

[67] Odriozola-Serrano I, Oms-Oliu G, MartÃn-Belloso O. Nanoemulsion-Based Delivery Systems to Improve Functionality of Lipophilic Components. Front Nutr 2014;1. https://doi.org/10.3389/fnut.2014.00024.

[68] Siwik DA, Pagano PJ, Colucci WS. Oxidative stress regulates collagen synthesis and matrix metalloproteinase activity in cardiac fibroblasts. American Journal of Physiology-Cell Physiology 2001;280:C53–60. https://doi.org/10.1152/ajpcell.2001.280.1.C53.

[69] Hussen NH amin, Abdulla SK, Ali NM, Ahmed VA, Hasan AH, Qadir EE. Role of antioxidants in skin aging and the molecular mechanism of ROS: A comprehensive review. Aspects of Molecular Medicine 2025;5:100063. https://doi.org/10.1016/j.amolm.2025.100063.

Click here to access/download;Figure;Figure 12b.tiff

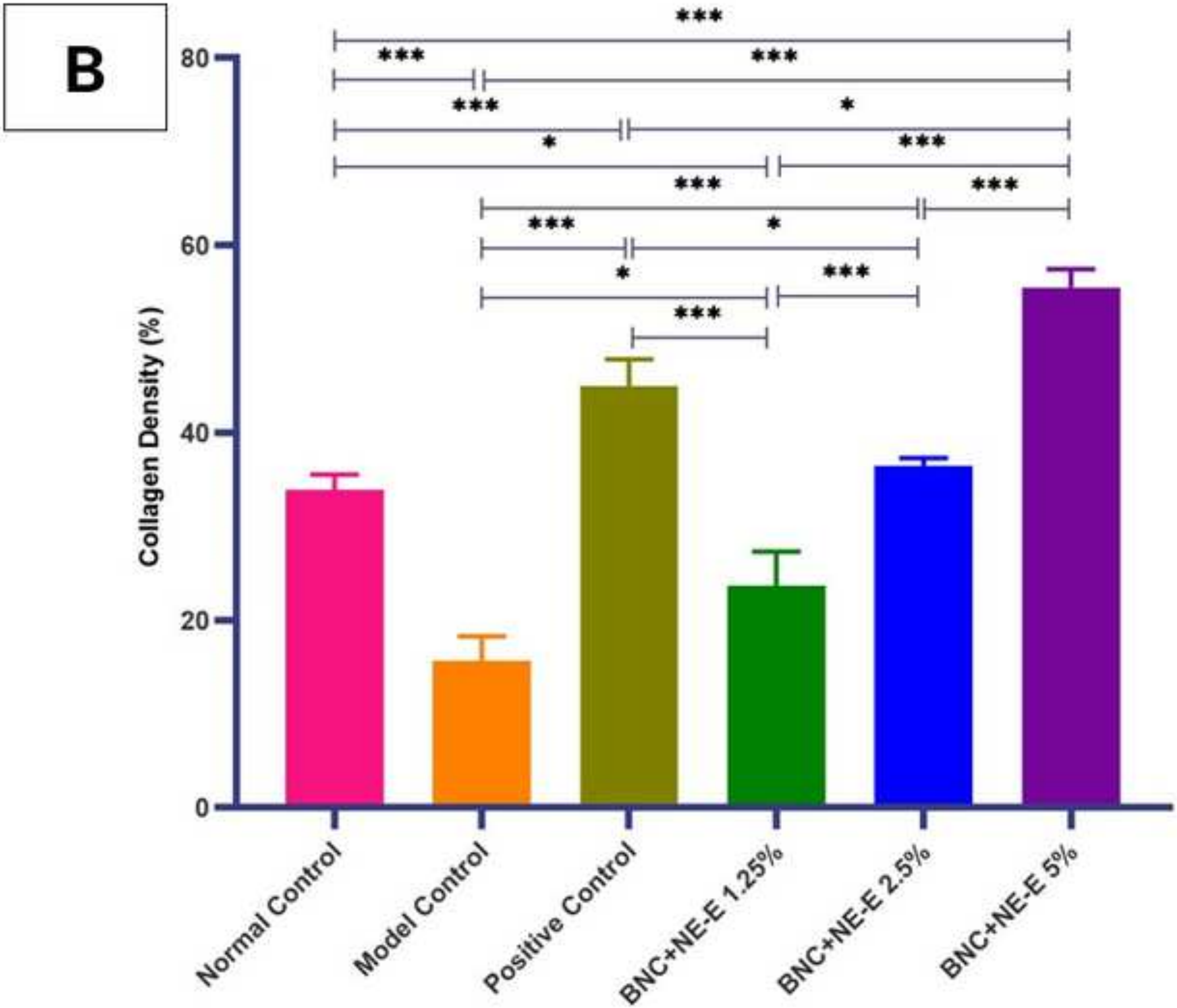

Click here to access/download;Figure;Figure 12b.tif

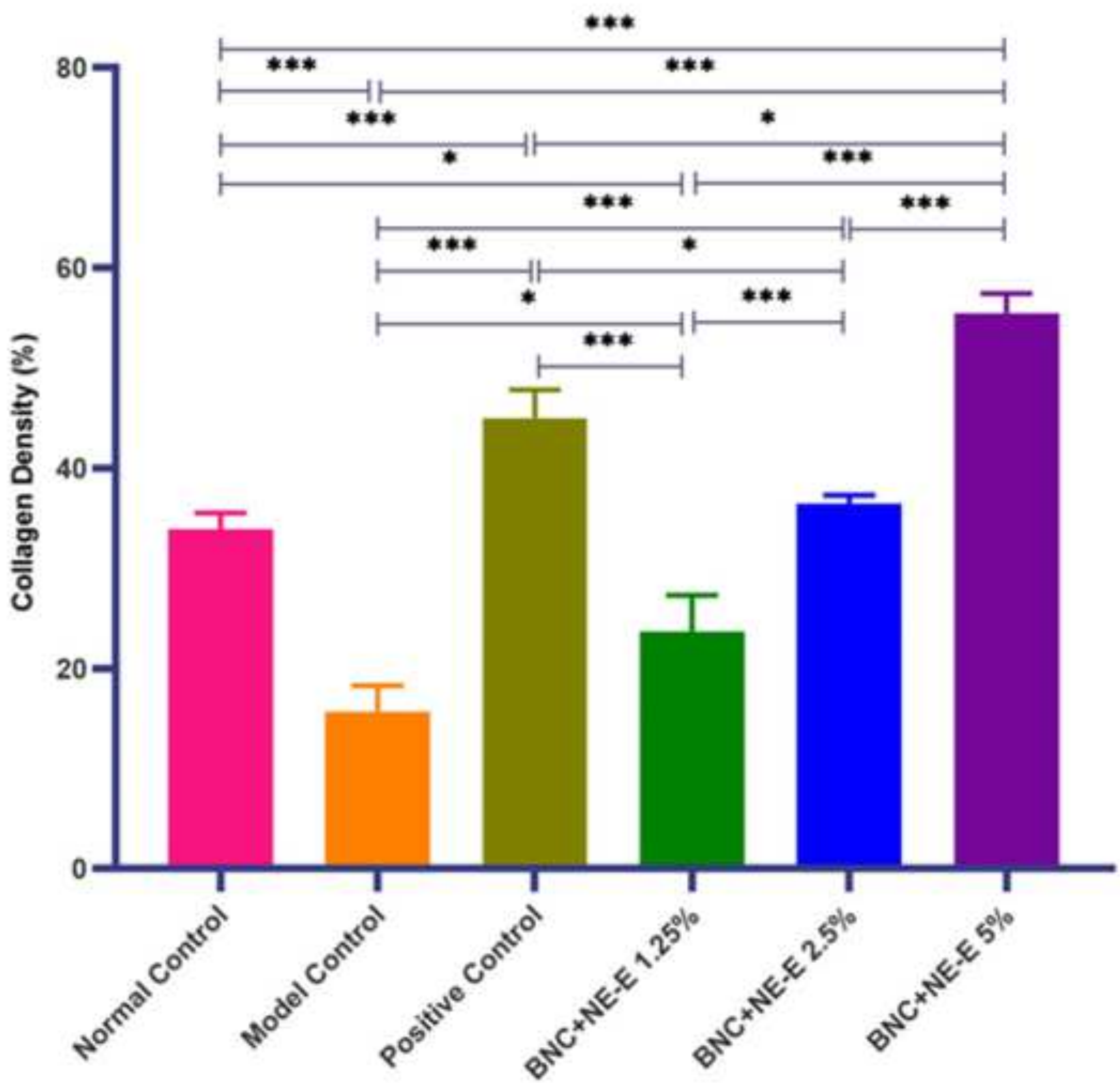

Click here to access/download;Figure;Figure 12a.tiff

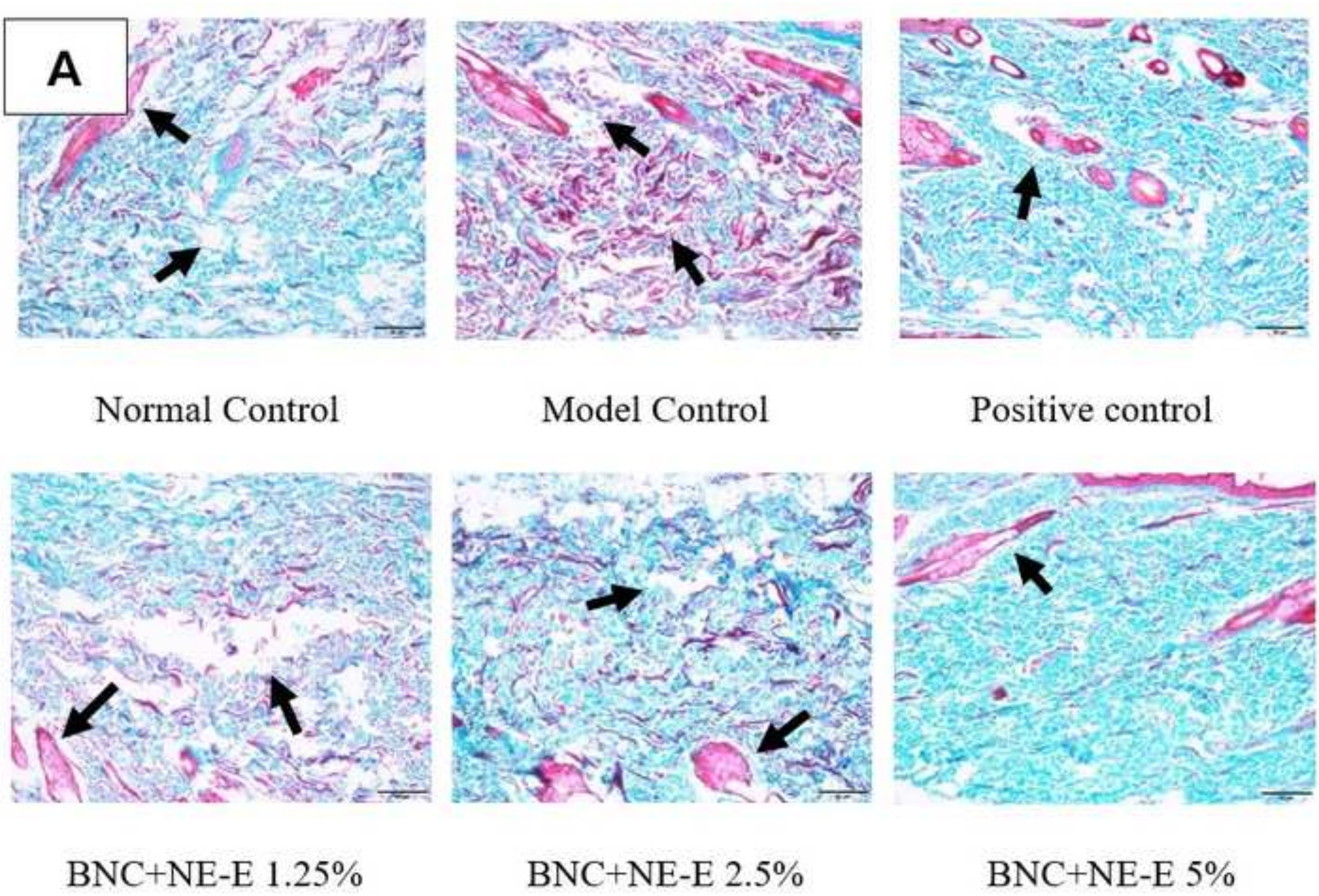

Click here to access/download;Figure;Figure 11.tif

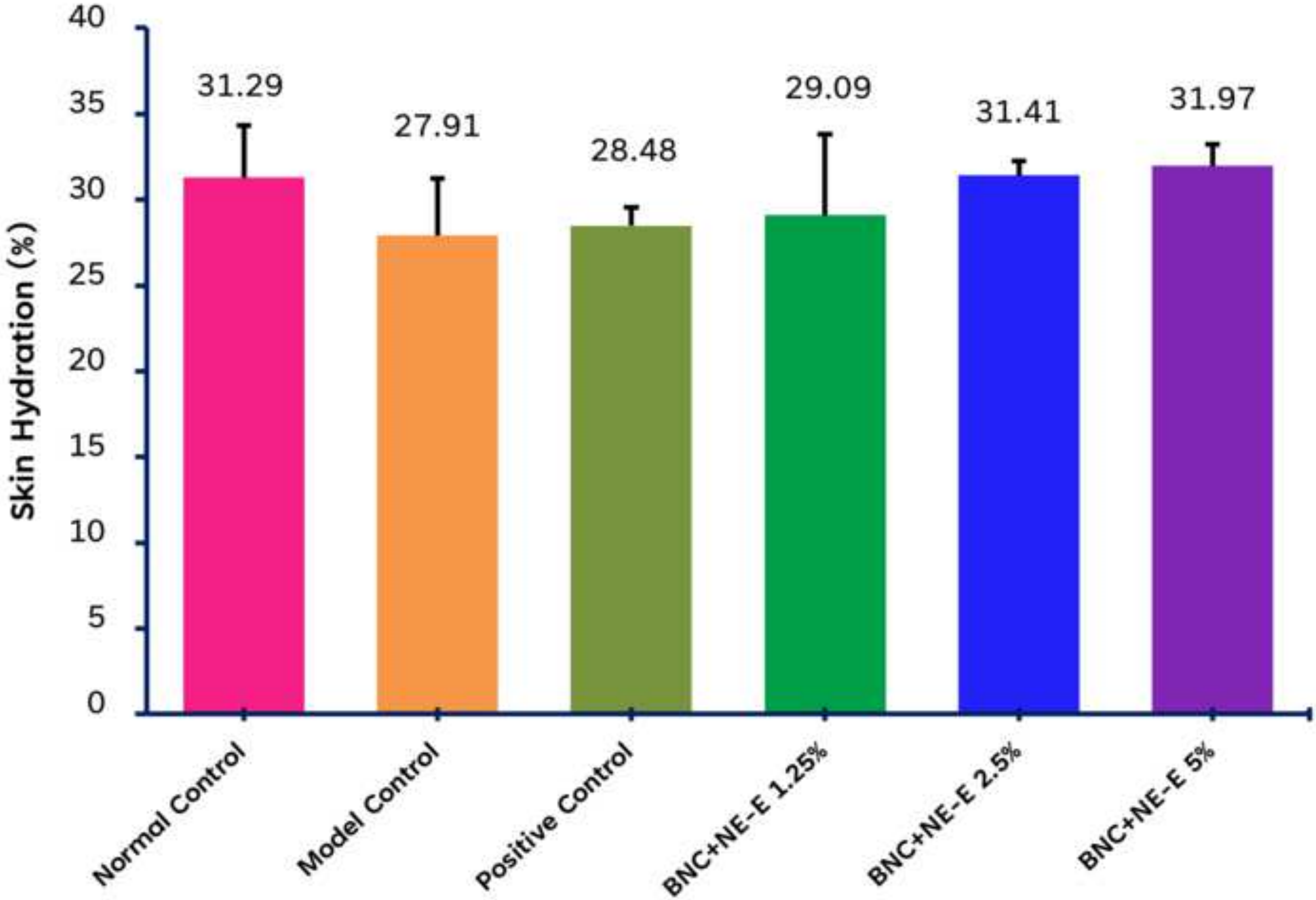

Click here to access/download;Figure;Figure 10.tif

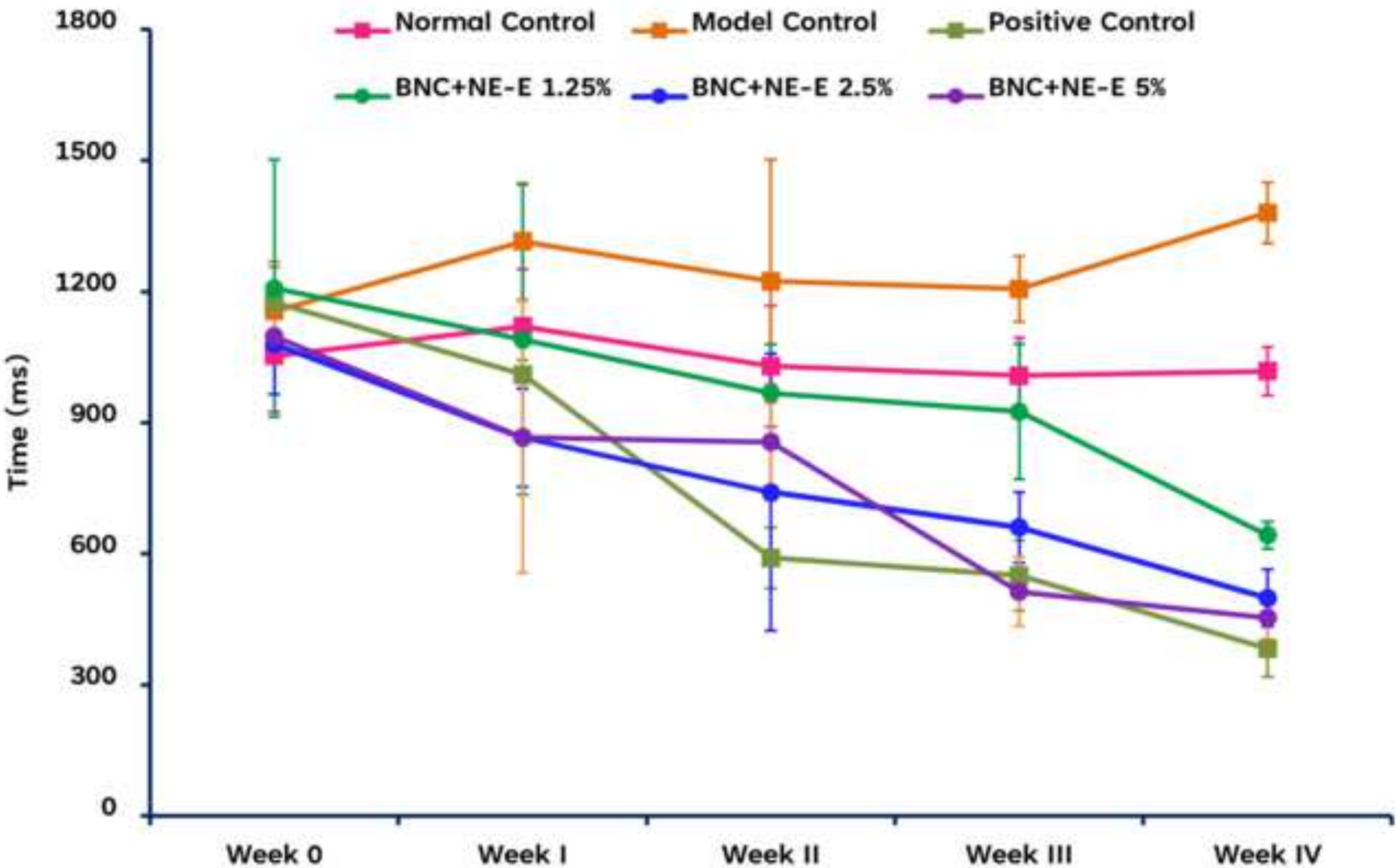

Click here to access/download;Figure;Figure 9b.tiff

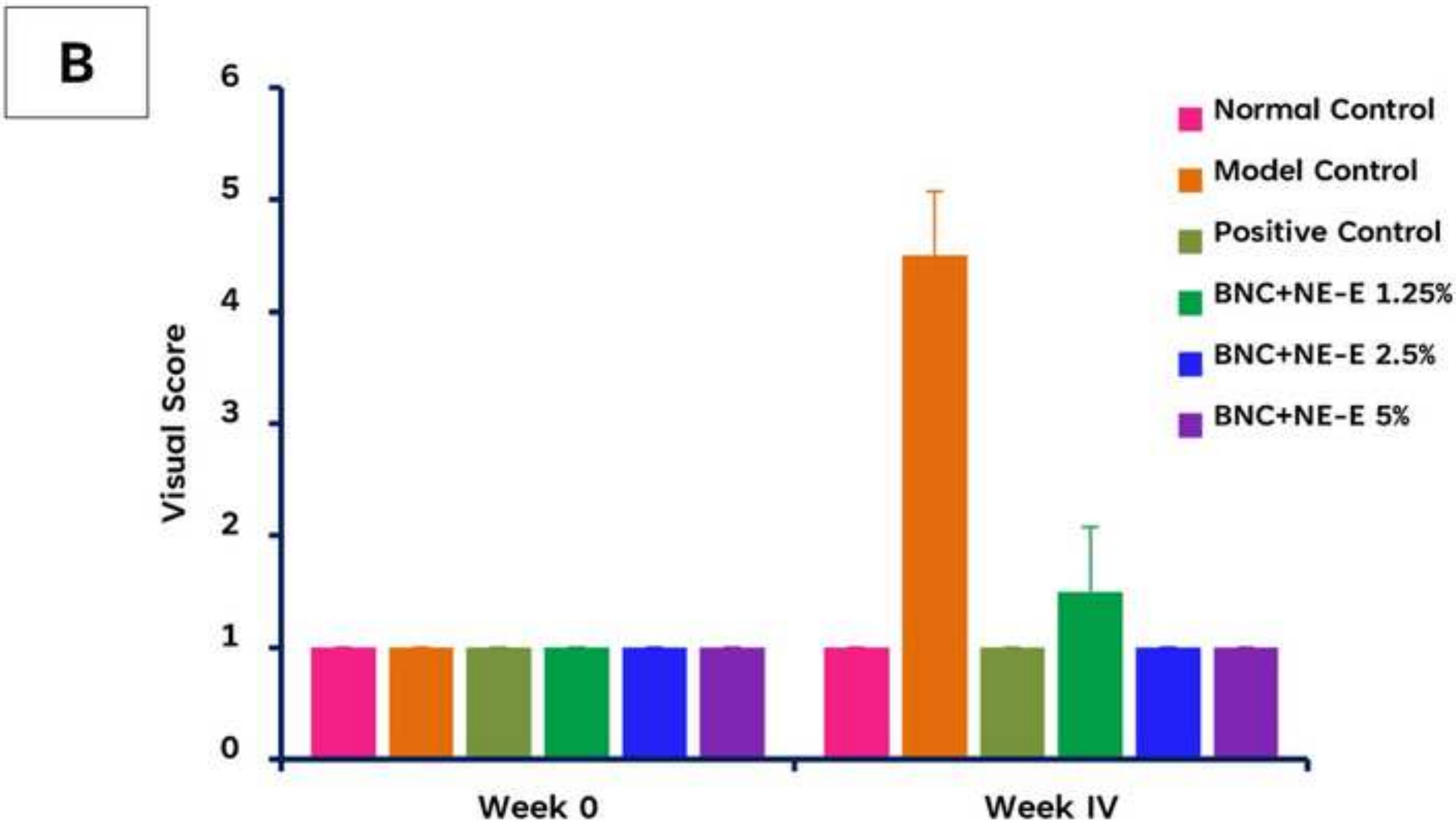

Click here to access/download;Figure;Figure 9b.tif

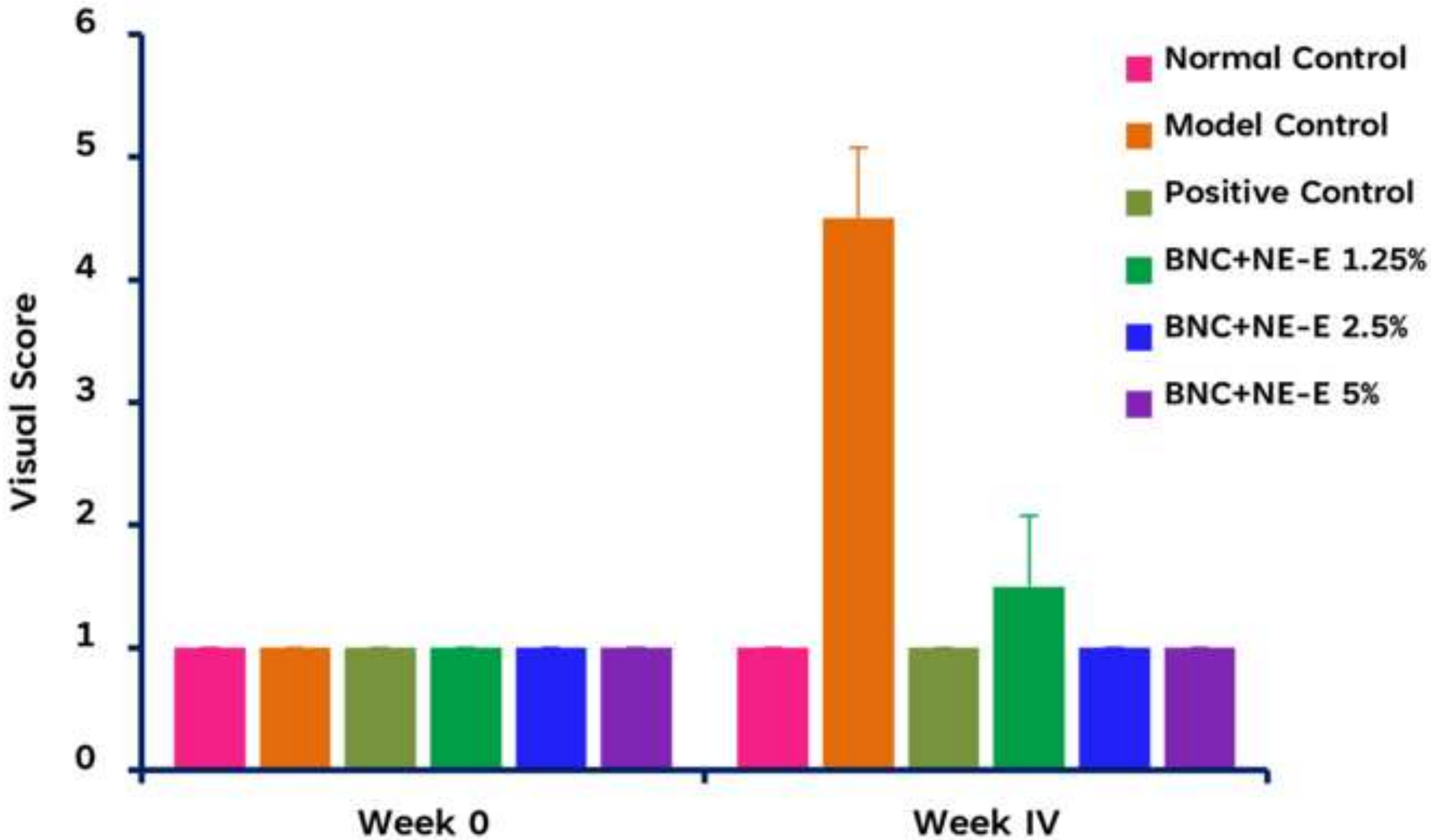

Click here to access/download;Figure;Figure 9a.tiff

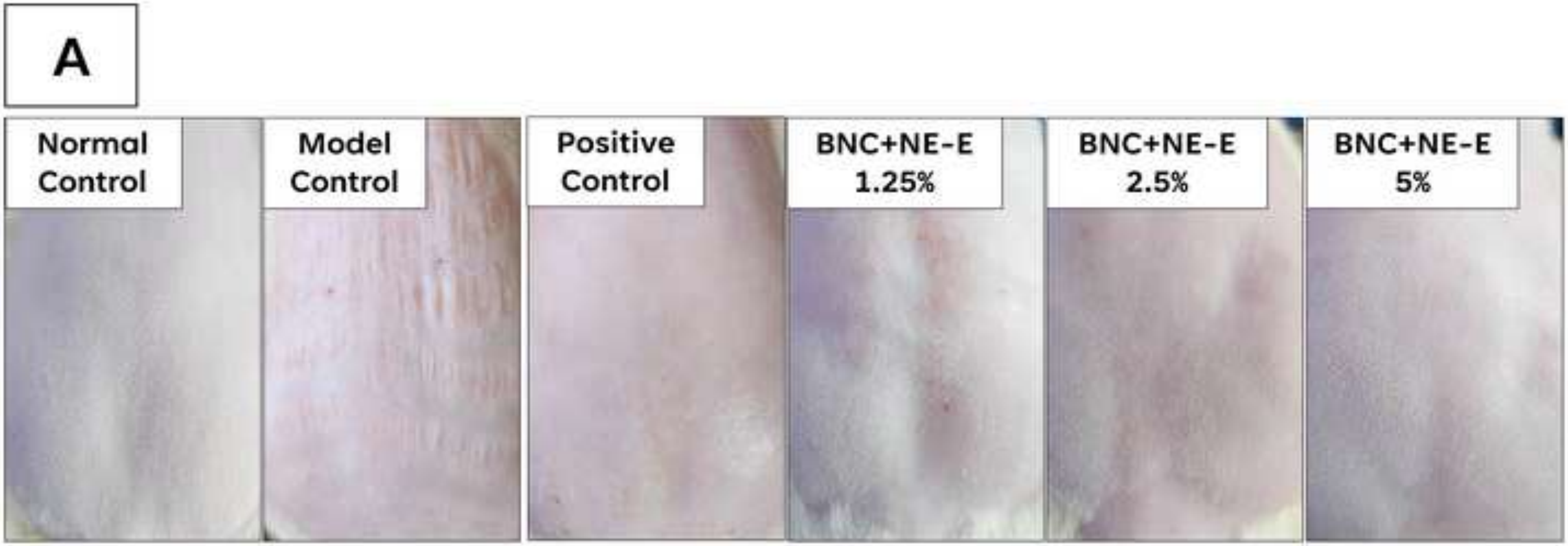

Click here to access/download;Figure;Figure 9.tiff

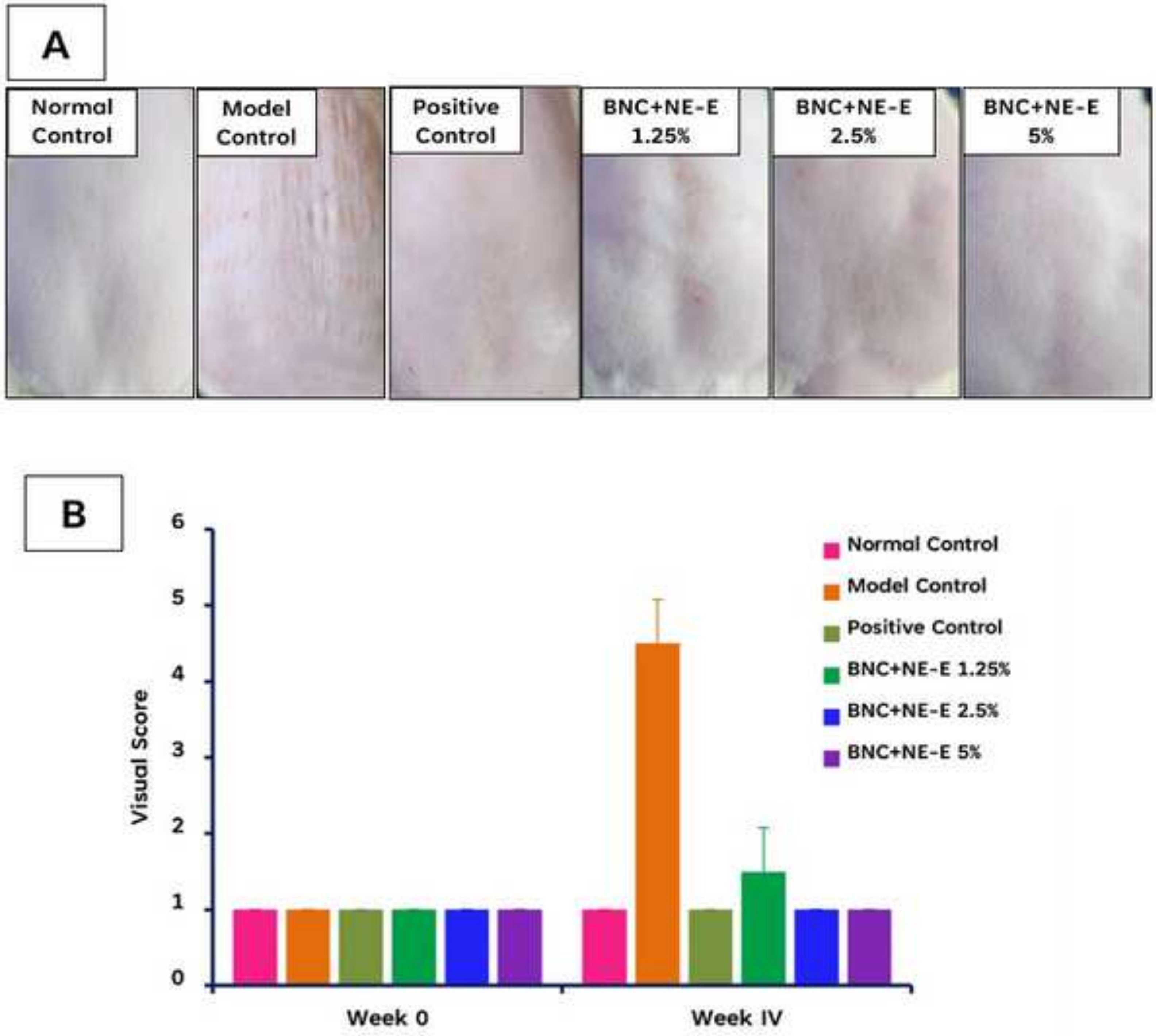

Click here to access/download;Figure;Figure 8.tif

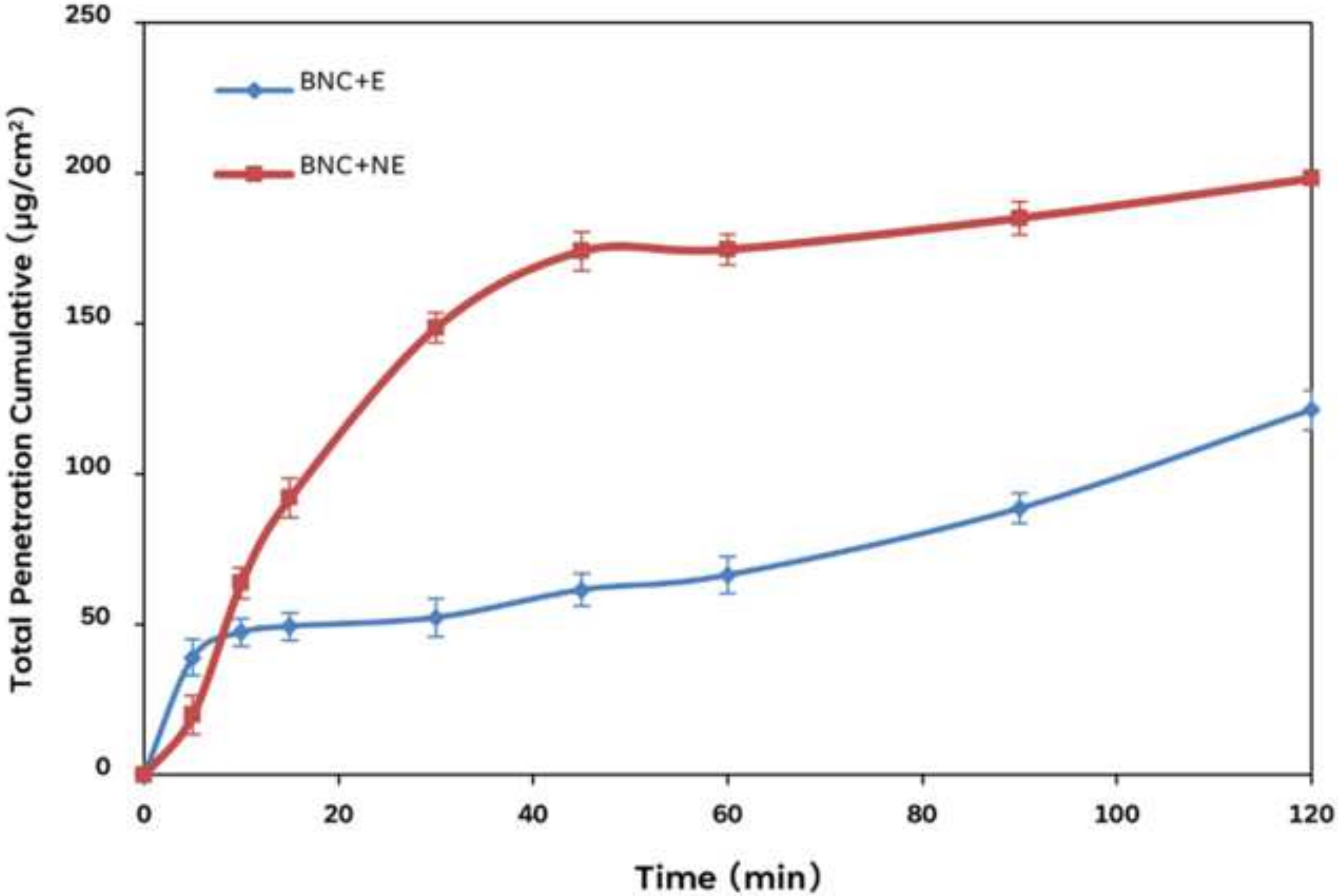

Click here to access/download;Figure;Figure 7.tif

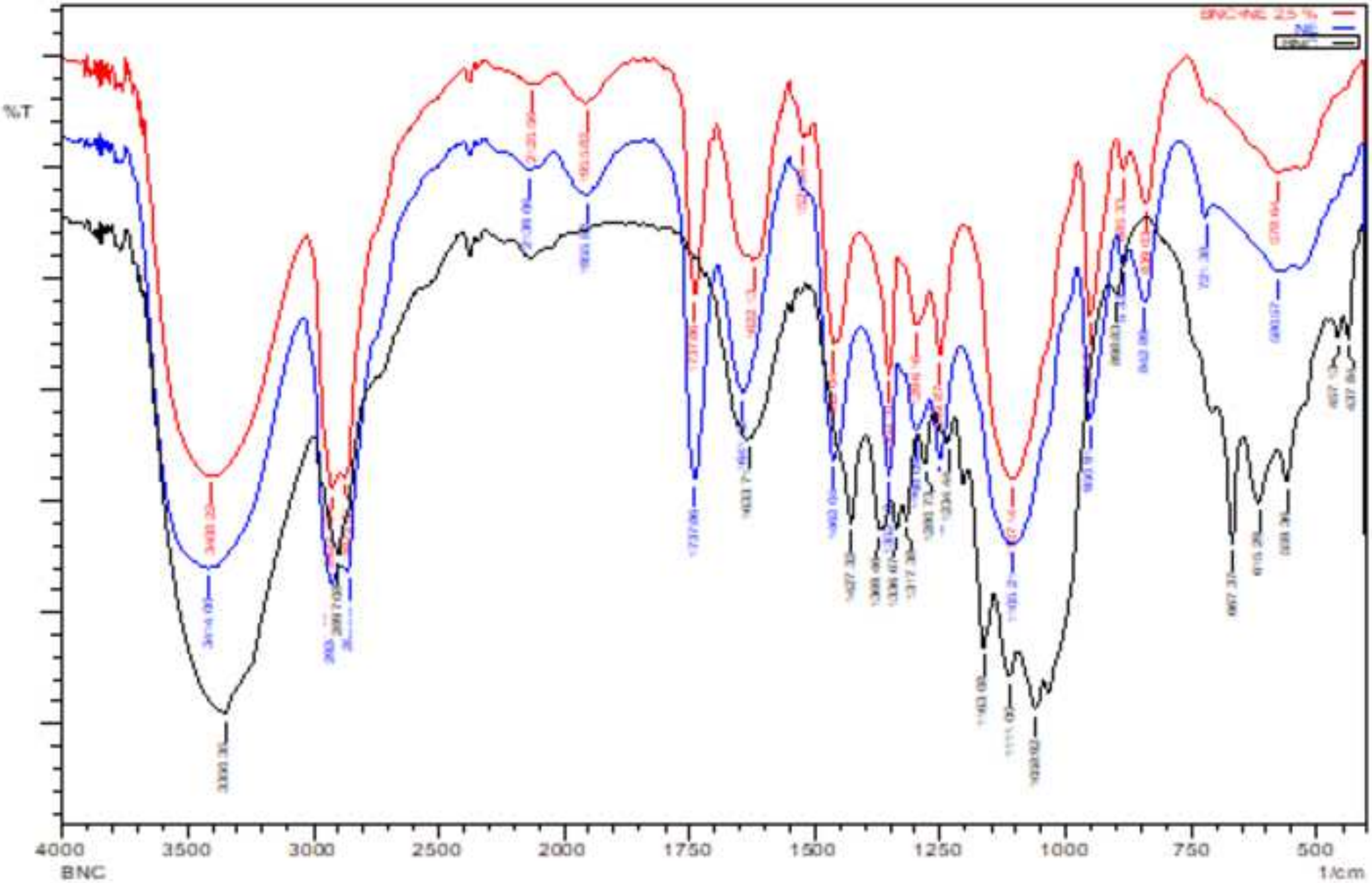

Click here to access/download;Figure;Figure 6.tif

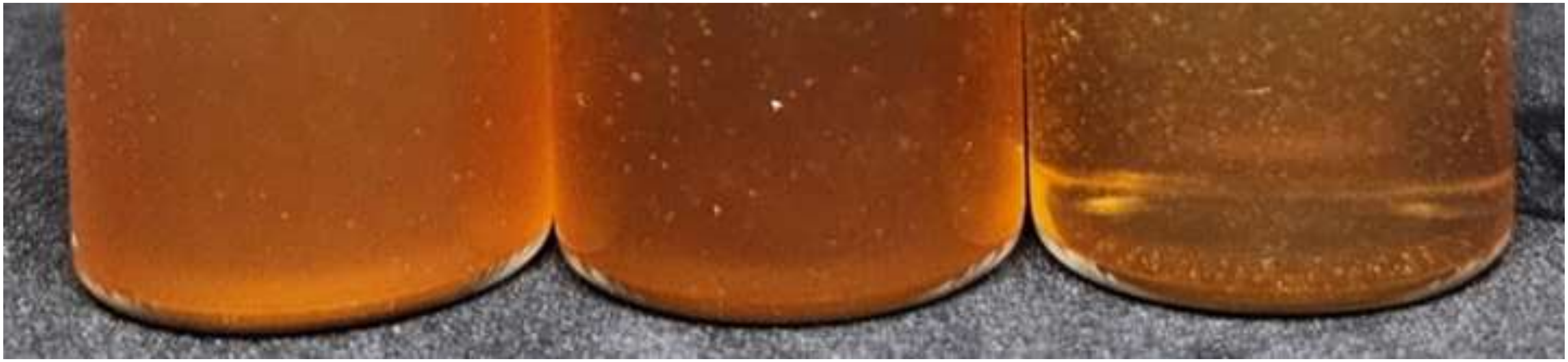

Click here to access/download;Figure;Figure 5.tiff

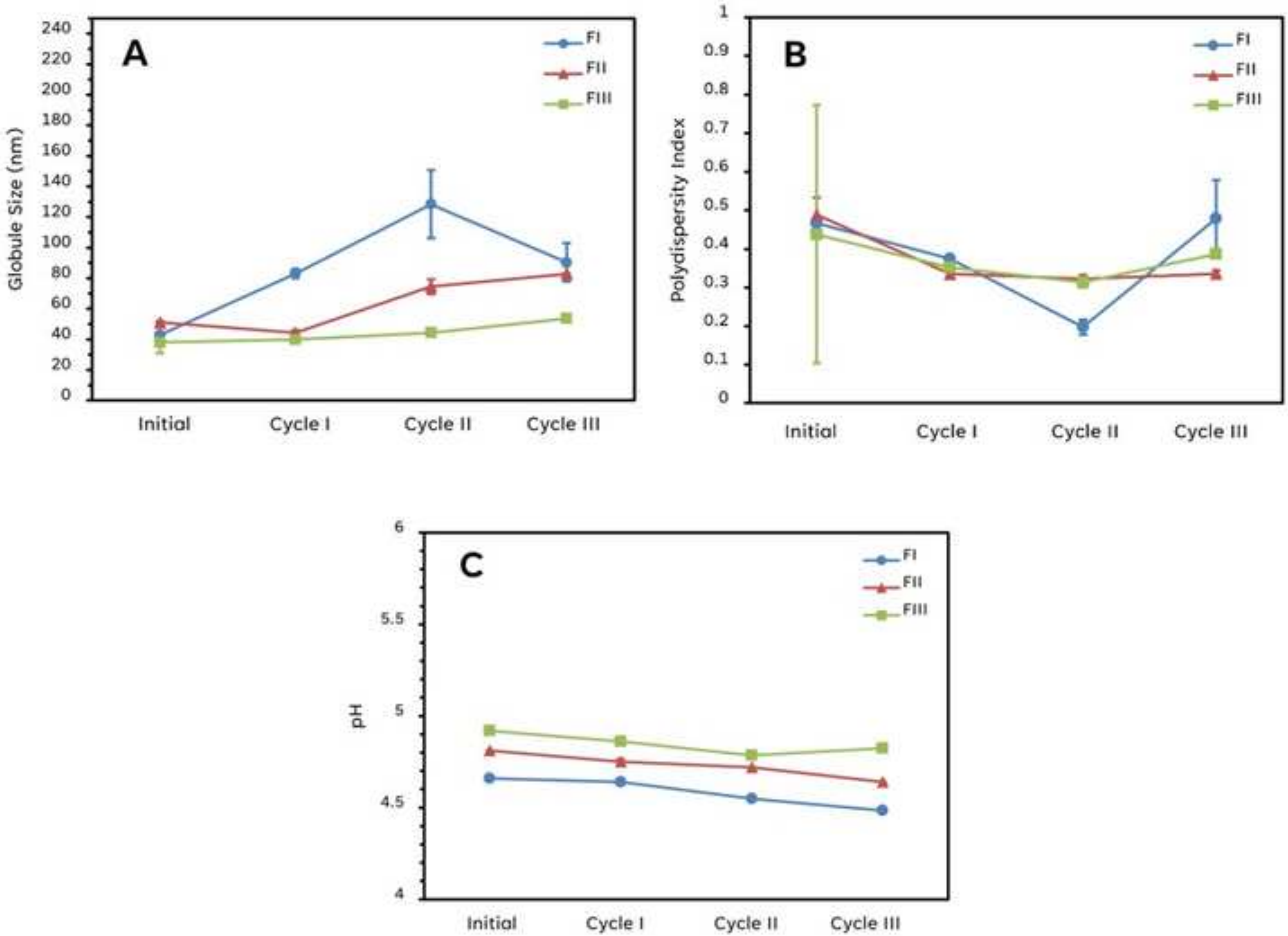

Click here to access/download;Figure;Figure 4.tiff

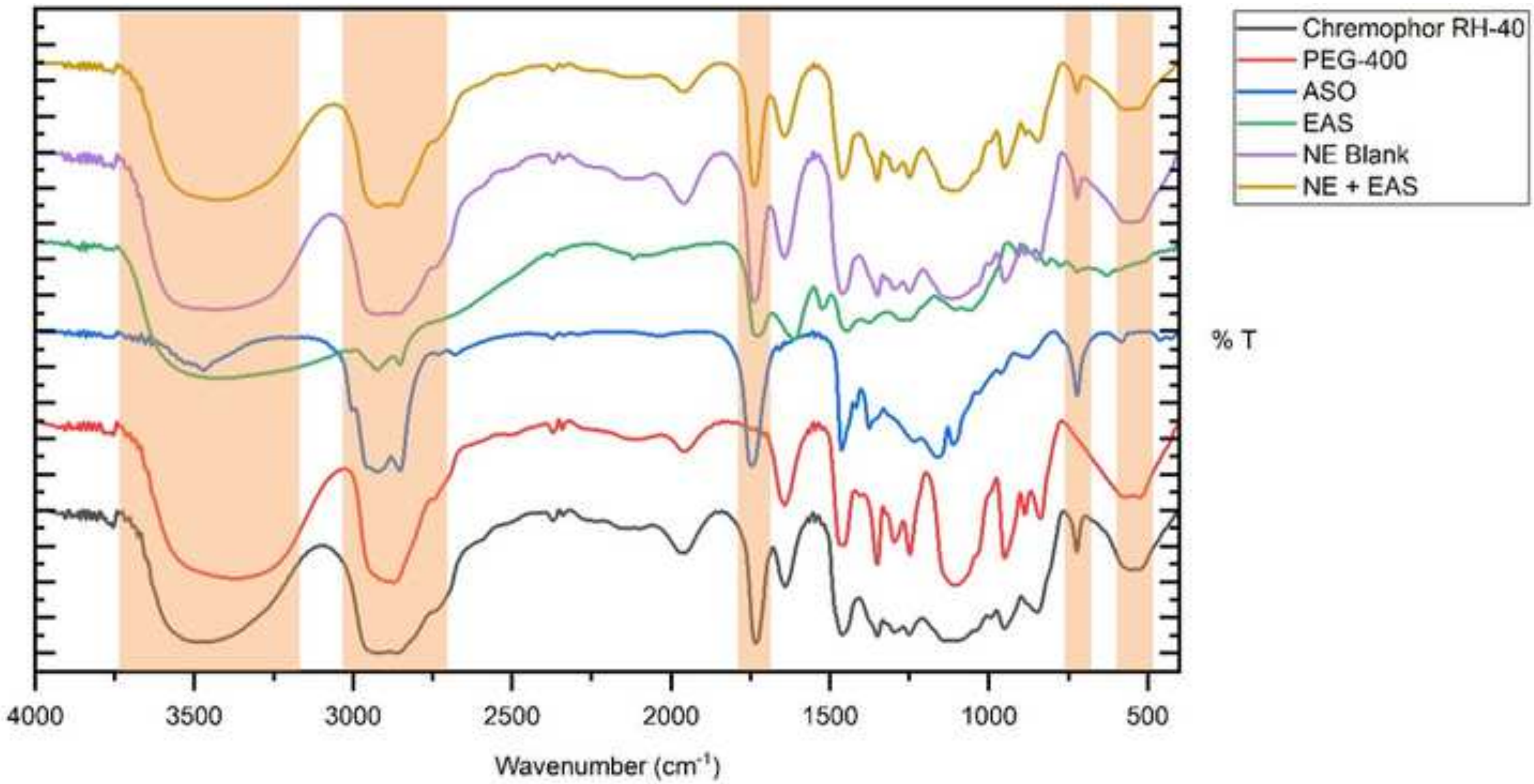

Click here to access/download;Figure;Figure 3.tiff

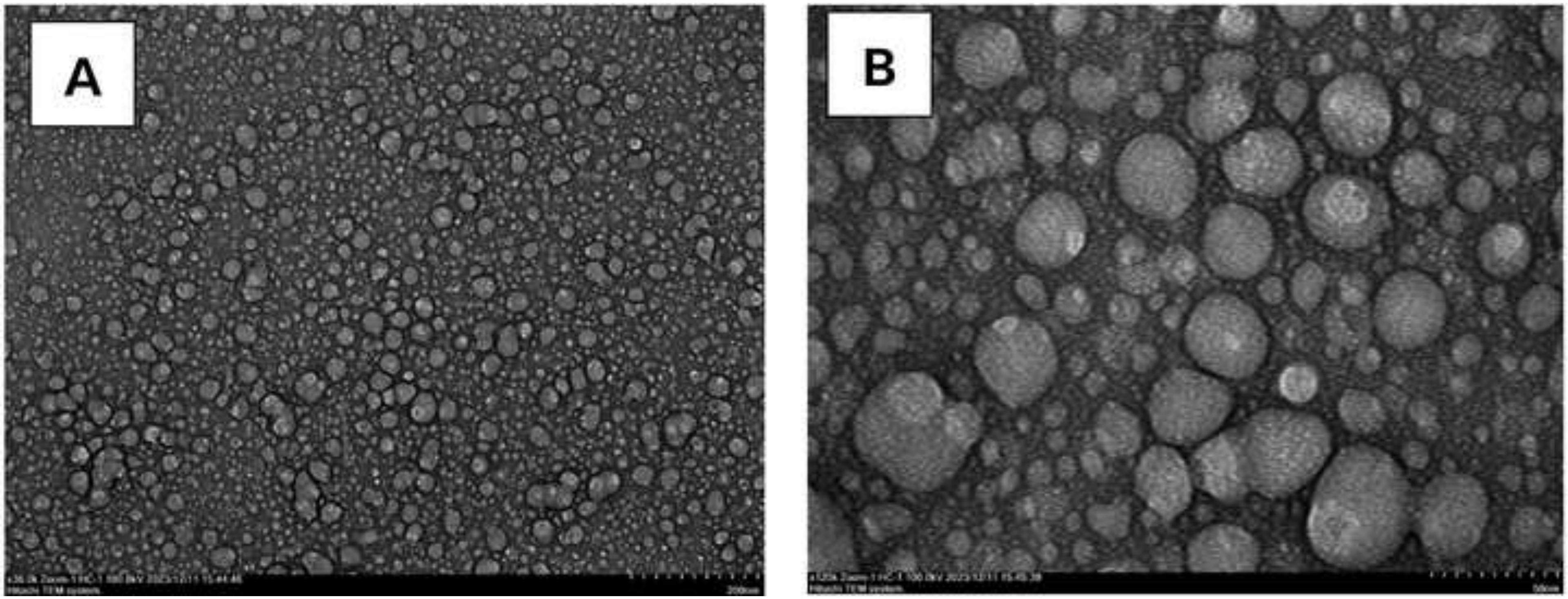

Click here to access/download;Figure;Figure 2.tiff

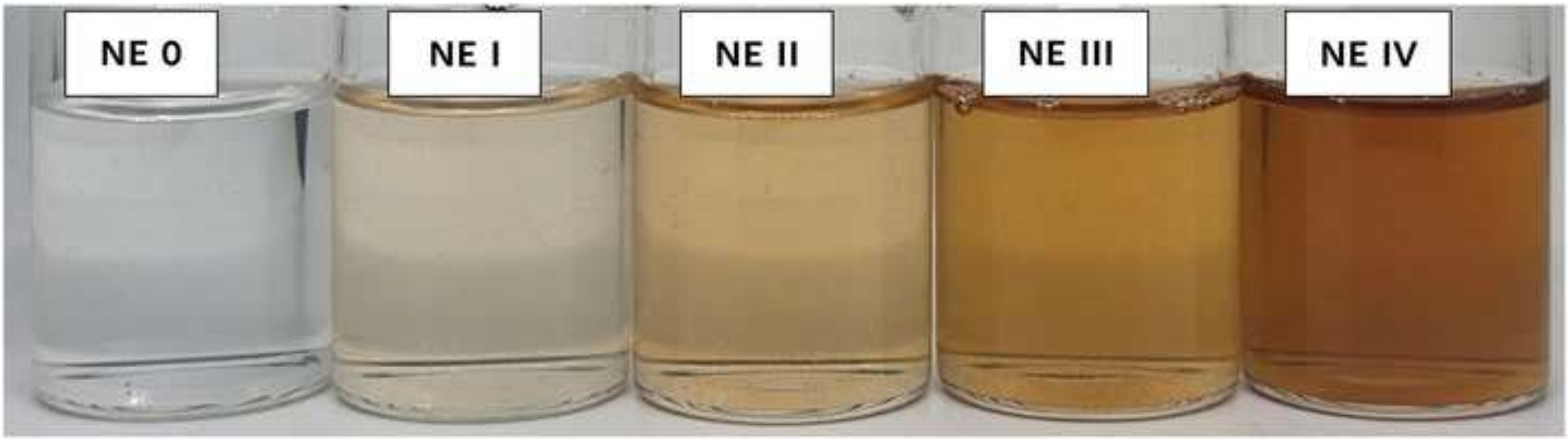

Click here to access/download;Figure;Figure 1.tiff

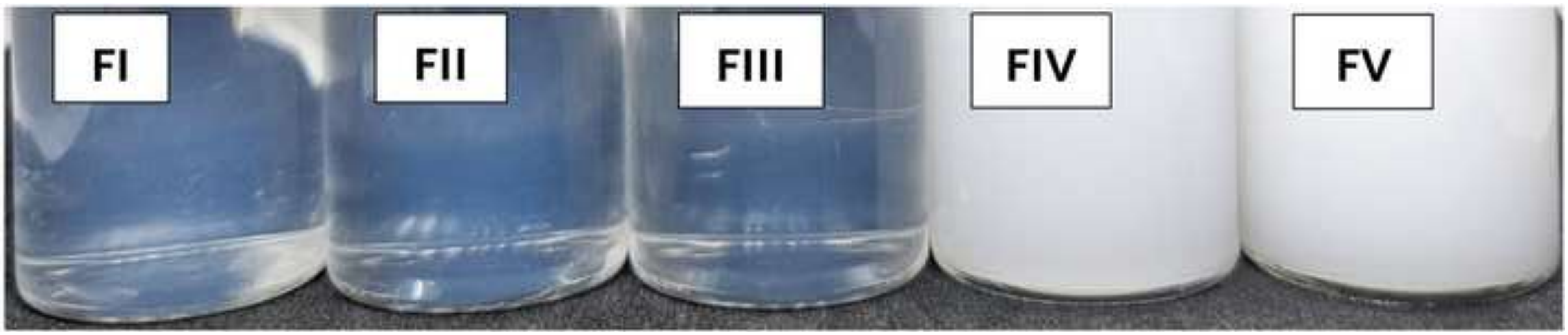

Supplement: Supplementary file 1 [file pone.0359807.s002.pdf]
