# Supplementary material for: Formulation and biological evaluation of a bacterial nanocellulose-based nanoemulsion hydrogel loaded with Persea americana Mill. seed extract: An anti-aging assessment
Source: PLoS One. 2026 Nov 5;21(11):e0359807. doi: 10.1371/journal.pone.0359807 (PMC13739224; doi:10.1371/journal.pone.0359807)
Supplement: Supplementary file 2 [file pone.0359807.s001.docx]

**Table 1.** Formulation development and selection framework for the BNC-based nanoemulsion hydrogel: material variables, process conditions, quality attributes, and selection considerations.

| **Development stage** | **Variable or condition** | **Formulation-development descriptor** | **Range or setting** | **Attribute evaluated / selection consideration** | **Decision or relevance to product performance and reproducibility** |
| --- | --- | --- | --- | --- | --- |
| Preliminary nanoemulsion screening | ASO:Cremophor® RH40:PEG 400 ratio | **Material variable (CMA-related)** | FI–FV ratios: 1:8:1, 1:7:2, 1:6:3, 2:7:1, and 3:6:1 | Clear appearance; globule size preferably <100 nm; PDI <0.3; high transmittance; skin-compatible pH | FIII (1:6:3) was selected as the nanoemulsion precursor because it showed a globule size of 20.3 ± 0.10 nm, PDI of 0.109 ± 0.029, transmittance of 99.62 ± 0.27%, and pH of 4.92 ± 0.01. |
| Nanoemulsion preparation | Order of ingredient addition | **Process condition (CPP-related)** | Oil-phase components were mixed before aqueous-phase addition; BNC was incorporated after nanoemulsion preparation | Component dispersion, formulation clarity, and homogeneity | A fixed sequence was applied to all formulations to support consistent nanoemulsion formation and subsequent BNC incorporation. |
| Nanoemulsion preparation | Oil-phase mixing speed and duration | **Process condition (CPP-related)** | 500 rpm for 2 h | Uniform dispersion of oil, surfactant, co-surfactant, and extract-loaded components | Fixed mixing condition used during preparation of the screened formulations. |
| Nanoemulsion preparation | Bath sonication time | **Process condition (CPP-related)** | 1 h | Formation of nanoscale droplets with a relatively narrow size distribution | Fixed sonication condition used during preparation of the screened formulations. |
| Nanoemulsion preparation | Oil-phase-to-water ratio | **Material/process variable** | 1:3 | Optical clarity, emulsification behavior, and globule-size characteristics | Fixed water-addition ratio used in the selected formulation. |
| Extract-loading screening | Avocado seed extract concentration | **Material variable (CMA-related)** | 0.1–5.0% (w/w) | Maintain clear appearance, nanoscale globule size, low PDI, and high transmittance while increasing extract loading | 5.0% was selected as the maximum acceptable loading under the tested conditions. The extract-loaded nanoemulsion showed a globule size of 23.7 ± 1.01 nm, PDI of 0.196 ± 0.045, transmittance of 98.55 ± 0.47%, and pH of 4.79 ± 0.01. |
| BNC-based hydrogel formation | BNC incorporation | **Material variable (CMA-related)** | BNC incorporated into the selected extract-loaded nanoemulsion formulation | Final formulation homogeneity, serum-like topical properties, viscosity, and spreadability | BNC served as the hydrated nanofibrillar matrix of the final topical hydrogel. |
| BNC-based hydrogel formation | Temperature and mixing during BNC incorporation | **Process condition (CPP-related)** | 75 °C, 300 rpm, 15 min | Uniform BNC dispersion and formation of a homogeneous hydrogel network | This process condition was used to facilitate complete hydration and uniform dispersion of BNC. |
| BNC-based hydrogel formation | Final mechanical homogenization | **Process condition (CPP-related)** | 700 rpm for 15 min | Uniform distribution of BNC and acceptable consistency of the final formulation | Fixed final homogenization condition used to support reproducibility of the BNC-based hydrogel. |
| Final product physicochemical evaluation | Visual appearance and homogeneity | **Quality attribute evaluated (CQA-related)** | Clear and homogeneous appearance; absence of visible phase separation | Acceptable physical appearance and product uniformity | Used to confirm physical acceptability of the final formulation. |
| Final product physicochemical evaluation | pH, viscosity, and spreadability | **Quality attribute evaluated (CQA-related)** | Measured in the final formulation | Skin-compatible pH and acceptable topical application performance | The BNC-based formulation showed pH 5.41, viscosity 352.62 mPa·s, and spreadability 5.30 ± 0.20 cm. |
| Comparative performance evaluation | In vitro permeation through a cellophane membrane using Franz diffusion cells | **Comparative performance attribute** | BNC-based nanoemulsion hydrogel (BNC+NE) compared with BNC-based conventional emulsion (BNC+E) | Cumulative in vitro permeation relative to the conventional emulsion comparator | At 120 min, BNC+NE reached 198.28 ± 0.54 µg/cm², compared with 121.35 ± 0.54 µg/cm² for BNC+E. |

ASO, avocado seed oil; BNC, bacterial nanocellulose; PDI, polydispersity index; NE, nanoemulsion; E, conventional emulsion.

*The terms CMA-related, CPP-related, and CQA-related are used descriptively to map the formulation-development and selection process. Formal criticality was not established through a Quality by Design or Design of Experiments investigation.*
