# Supplementary material for: Formulation and biological evaluation of a bacterial nanocellulose-based nanoemulsion hydrogel loaded with Persea americana Mill. seed extract: An anti-aging assessment
Source: PLoS One. 2026 Nov 5;21(11):e0359807. doi: 10.1371/journal.pone.0359807 (PMC13739224; doi:10.1371/journal.pone.0359807)
Supplement: Supplementary file 3 [file pone.0359807.s003.docx]

**Review of Attachments for Manuscript Number PONE-D-26-10500
"Formulation and biological evaluation of a bacterial nanocellulose-based nanoemulsion loaded with Persea americana Mill. seed extract: An anti-aging assessment"**

1. Is it possible to dry the herbal sample by using oven. What will happen to the volatile oils present in it.
2. Seeds are dried using oven at 50 degree for 5 days is stated by the researcher. So I want to know for how much time the sample is kept in oven.
