# Supplementary material for: Formulation and biological evaluation of a bacterial nanocellulose-based nanoemulsion hydrogel loaded with Persea americana Mill. seed extract: An anti-aging assessment
Source: PLoS One. 2026 Nov 5;21(11):e0359807. doi: 10.1371/journal.pone.0359807 (PMC13739224; doi:10.1371/journal.pone.0359807)
Supplement: Supplementary file 4 [file pone.0359807.s005.docx]

| **Journal requirements:** | | |
| --- | --- | --- |
| No. |  |  |
| 1 | Please ensure that your manuscript meets PLOS ONE's style requirements, including those for file naming. The PLOS ONE style templates can be found at | Thank you for the reminder. We have reviewed the PLOS ONE formatting templates, and revised the manuscript and accompanying files accordingly, including the required file naming and formatting style. |
| 2. | To comply with PLOS One submissions requirements, in your Methods section, please provide additional information regarding the experiments involving animals and ensure you have included details on methods of (1) anesthesia and/or analgesia, and (2) efforts to alleviate suffering | Thank you for this comment. The Methods section has been revised to clarify the anesthesia procedures and the efforts used to alleviate animal suffering. |
| 3. | In your Methods section, please include the following details:  1. The source, age, and weight of the rats used in the animal experiments.  2. The source of the avocados used in the study. | Thank you for this comment. We have revised the Methods section to specify the source, age, and body weight of the rats used in the animal experiments. We have also clarified the source and botanical identification of the avocado material used in this study. |
| 4. | “This work was supported by Beasiswa Pendidikan Indonesia (BPI) (Grant No. 202101121555), Center for Higher Education Funding and Assessment (PPAPT) under the Ministry of Higher Education, Science, and Technology, Indonesia and the Indonesia Endowment Funds for Education (LPDP) under the Ministry of Finance, Indonesia.”  Please state what role the funders took in the study.  If the funders had no role, please state: "The funders had no role in study design, data collection and analysis, decision to publish, or preparation of the manuscript."  If this statement is not correct you must amend it as needed.  Please include this amended Role of Funder statement in your cover letter; we will change the online submission form on your behalf. | Thank you for the clarification. The funders had no role in study design, data collection and analysis, decision to publish, or preparation of the manuscript. We have included this role of funder statement in the cover letter, as requested. |
| 5. | Thank you for stating the following in the Acknowledgments Section of your manuscript:  “This work was supported by Beasiswa Pendidikan Indonesia (BPI) (Grant No. 202101121555), Center for Higher Education Funding and Assessment (PPAPT) under the Ministry of Higher Education, Science, and Technology, Indonesia and the Indonesia Endowment Funds for Education (LPDP) under the Ministry of Finance, Indonesia.”  We note that you have provided funding information that is currently declared in your Funding Statement. However, funding information should not appear in the Acknowledgments section or other areas of your manuscript. We will only publish funding information present in the Funding Statement section of the online submission form.  Please remove any funding-related text from the manuscript and let us know how you would like to update your Funding Statement. Currently, your Funding Statement reads as follows:  “This work was supported by Beasiswa Pendidikan Indonesia (BPI) (Grant No. 202101121555), Center for Higher Education Funding and Assessment (PPAPT) under the Ministry of Higher Education, Science, and Technology, Indonesia and the Indonesia Endowment Funds for Education (LPDP) under the Ministry of Finance, Indonesia.”  Please include your amended statements within your cover letter; we will change the online submission form on your behalf. | Thank you for the clarification. We have removed all funding-related text from the Acknowledgments section and from all other sections of the manuscript. |
| 6. | PLOS requires an ORCID iD for the corresponding author in Editorial Manager on papers submitted after December 6th, 2016. Please ensure that you have an ORCID iD and that it is validated in Editorial Manager. To do this, go to ‘Update my Information’ (in the upper left-hand corner of the main menu), and click on the Fetch/Validate link next to the ORCID field. This will take you to the ORCID site and allow you to create a new iD or authenticate a pre-existing iD in Editorial Manager. | The corresponding author’s ORCID iD has been validated in Editorial Manager as requested. |
| 7. | Please include your full ethics statement in the ‘Methods’ section of your manuscript file. In your statement, please include the full name of the IRB or ethics committee who approved or waived your study, as well as whether or not you obtained informed written or verbal consent. If consent was waived for your study, please include this information in your statement as well. | Thank you for this clarification. We have added a complete ethics statement to the Methods section, including the full name of the animal ethics committee and the ethical approval number. As this study involved laboratory animals and did not involve human participants, informed consent was not applicable. |

| **Additional Editor Comments** | | |
| --- | --- | --- |
| No. |  |  |
| 1. | What is the advantage of combining bacterial nanocellulose with a nanoemulsion instead of using the extract or nanoemulsion alone | Advantage of combining BNC with nanoemulsion:  The combination provides dual functionality. The nanoemulsion improves the dispersion of avocado seed bioactive constituents and supports their in vitro diffusion, whereas BNC provides a hydrated three-dimensional nanofibrillar matrix that may support formulation consistency, moisture retention, and local retention at the application site. Compared with the BNC-based conventional emulsion, BNC+NE showed higher cumulative in vitro diffusion, reaching 198.28 ± 0.54 µg/cm^2^ at 120 min versus 121.35 ± 0.54 µg/cm^2^ for BNC+E. |
| 2. | The abstract is currently too general and lacks sufficient technical details. Please revise it according to the standard format of a research article, including the study objective, methodology, key findings with important numerical results, and the main conclusion. | Thank you for this helpful comment. The abstract has been substantially revised to follow a standard research-article format. It now clearly states the study objective methods, numerical findings, and conclusion. Numerical data to be added include optimized nanoemulsion globule size of 20.3 ± 0.10 nm, PDI 0.109 ± 0.029, transmittance 99.62 ± 0.27%, pH 4.92 ± 0.01, permeation of 198.28 ± 0.54 µg/cm² after 120 min, and significant collagen improvement in the UVB-induced photoaging model. |
| 3. | The Introduction is descriptive and lacks sufficient technical justification. Please clearly discuss the formulation strategy, ingredient selection, CQAs, CMAs, CPPs, optimization approach, evaluation parameters, expected outcomes, research gap, novelty, and objectives of the study. | The Introduction has been revised to strengthen the technical justification for the formulation strategy. The revised text clarifies the rationale for selecting the nanoemulsion system and BNC matrix, the key formulation and process variables, the sequential screening approach, evaluation parameters, expected outcomes, research gap, novelty, and study objectives. These revisions have been highlighted in the Introduction.  Detailed formulation development considerations are further summarized in Supplementary File. |
| 4. | The statement is technically inaccurate for a conventional serum formulation, as serum products are not necessarily dependent on gelling agents. Please clarify the developed formulation properties. | We agree that conventional serum products are not necessarily dependent on gelling agents. The statement has been revised to clarify that the developed product is not a conventional liquid serum, but a BNC-based nanoemulsion hydrogel with serum-like topical properties. The formulation exhibited a transparent appearance, relatively low viscosity, good spreadability, and a skin-compatible pH. These revisions have been highlighted in the Introduction, Methods, Results, and Discussion sections. |
| 5. | Verify reference 10. Many other references are required to be verified again for their correctness of use as per study. | Reference 10 and all others have been carefully re-checked for their relevance, accuracy, and appropriateness in supporting the corresponding statements. |
| 6. | These properties enable BNC not only to function as a gelling agent. How? Justify technically. | BNC contributes beyond simple viscosity enhancement. Its nanofibrillar network possesses high water-holding capacity, biocompatibility, film-forming ability, and a porous three-dimensional structure. These characteristics may support skin hydration, prolong local contact of the formulation with the skin, provide a reservoir effect, and modulate the release of incorporated nanoemulsion droplets. |
| 7. | The extraction procedure requires additional details to ensure reproducibility. Please specify the amount of avocado seed powder used, solvent-to-solid ratio, extraction volume, extraction temperature, agitation conditions (if any), extraction yield, and the final drying/storage conditions of the extract. The rationale for selecting ethanol and performing three maceration cycles should also be provided. | The extraction method has been revised to include the avocado seed powder weight, solvent-to-solid ration, extraction volume, extraction temperature, agitation condition, filtration method, concentration procedure, and storage condition.  Extraction yield result has been reported in the Results section. |
| 8. | How was the nanoemulsion design selected? No pseudo-ternary phase diagram. No screening study. Please provide data/results for those. | The nanoemulsion composition was selected through preliminary empirical screening rather than by construction of a pseudo-ternary phase diagram. Five formulations containing different ratios of avocado seed oil, Cremophor RH40, and PEG 400 were prepared and evaluated based on visual appearance, globule size, polydispersity index, transmittance, and pH. Among the tested formulations, FIII was selected because it produced the smallest globule size, the lowest PDI, high transmittance, and a suitable pH. The Methods and Results sections have been revised to clearly describe the screening approach, selection criteria, and supporting data. |
| 9. | Various concentrations of avocado seed extract, ranging from 0.1% to 5.0%,, on what basis? Justify. | The concentration range was selected to evaluate loading capacity and formulation tolerance. The results showed that up to 5.0% extract could be incorporated without loss of clarity, phase separation, or unacceptable increase in globule size/PDI. Therefore, 5.0% was considered the maximum acceptable loading under the tested conditions. |
| 10. | CPP information are missing. | The critical process parameters used during formulation preparation have been clarified in the Methods section.  Specifically, the nanoemulsion was prepared by mixing the oil phase at 500 rpm for 2 h, followed by bath sonication for 1 h and addition of water at an oil-phase-to-water ratio of 1:3. During BNC incorporation, the formulation was mixed at 300 rpm for 15 min, followed by mechanical homogenization at 700 rpm for 15 min. These parameters were controlled to obtain a homogeneous and stable BNC-based nanoemulsion hydrogel. The use of 75^o^C will be re-evaluated and justified or reduced if necessary. |
| 11. | Optimization criteria are missing for formulation. | Optimization criteria of nanoemulsion will be added: clear appearance, globule size preferably <100 nm, polydispersity index < 0.3, transmittance close to 100%, a skin-compatible pH, and absence of phase separation.  Optimization criteria of BNC-based nanoemulsion hydrogel will be (has been) added: absence of phase separation, acceptable viscosity and spreadability, physical stability, and superior permeation |
| 12. | The nanoemulsion development strategy, formulation optimization approach, critical material attributes (CMAs), critical process parameters (CPPs), and critical quality attributes (CQAs) are not adequately described and should be clearly presented to ensure scientific rigor and reproducibility. | The nanoemulsion development strategy, formulation optimization approach, critical material attributes (CMAs), critical process parameters (CPPs), and critical quality attributes (CQAs) have been clarified in the revised manuscript.  A detailed summary of these attributes, process conditions, optimization criteria, and their relevance to product performance and reproducibility has been provided in S1_Table file. |
| 13. | Verify Reference 29. | Reference 29 has been removed and replaced with an appropriate reference supporting the BNC preparation method. |
| 14. | The BNC preparation method lacks critical fermentation, purification, and characterization details. Please provide sufficient information regarding inoculum preparation, fermentation conditions, purification procedures, yield, and characterization to ensure reproducibility of the study. | The bacterial nanocellulose preparation method has been substantially expanded in the revised manuscript. The revised section now includes the bacterial strain source, starter-culture preparation, Hestrin–Schramm medium composition, inoculum activation conditions, inoculum proportion, fermentation temperature, duration, static culture condition, purification procedure, neutralization step, and storage condition before formulation use. |
| 15. | How much BNC was added? | Purified wet BNC was dispersed in water to obtain a 20% (w/w) wet BNC dispersion. This information has been clarified in the Methods section and Table 6. |
| 16. | After other raw materials--???? | We agree that the phrase “after other raw materials were added” was unclear. The Methods section has been revised to specify the preparation sequence. The ambiguous phrase has been removed |
| 17. | Why 75°C? Could affect nanoemulsion droplet size and stability. Could degrade phytoconstituents. Justify. | The BNC incorporation temperature of 75^o^C was adapted from the previously reported method for preparing BNC-containing plant-based facial serum (reference 31).  We agree that elevated temperature may affect nanoemulsion droplet characteristics and phytoconstituent stability, but the final formulation remained visually homogeneous, with no visible phase separation, and showed acceptable viscosity, spreadability, pH, and physical stability.  Therefore, this temperature has now been identified as a controlled process condition parameter in the Supplementary Files. |
| 18. | How does BNC convert the nanoemulsion into a serum? | BNC does not chemically convert the nanoemulsion. Instead, it physically entraps and immobilizes the nanoemulsion droplets within its hydrated nanofibrillar network, increasing viscosity and producing a serum-like hydrogel consistency suitable for topical application. |
| 19. | What is the final dosage form? | The final dosage form will be clearly defined as a BNC-based avocado seed extract nanoemulsion hydrogel for topical anti-aging application. |

| **Reviewer Comments** | | |
| --- | --- | --- |
| No. |  |  |
| 1. | Is it possible to dry the herbal sample by using oven. What will happen to the volatile oils present in it. | Drying herbal materials in an oven is a commonly used method to reduce moisture content and prevent microbial growth. In this study, avocado seeds were dried at a relatively low temperature (60°C) to minimize degradation of thermolabile constituents. Although some loss of volatile compounds may occur during oven drying, avocado seeds are not primarily valued for their volatile oil content. The major bioactive compounds targeted in this study are phenolic compounds, catechins, and fatty acids, which are relatively stable under the selected drying conditions. Therefore, oven drying was considered appropriate for obtaining a stable and reproducible raw material for extraction. The rationale has been clarified in the revised manuscript. |
| 2. | Seeds are dried using oven at 50 degree for 5 days is stated by the researcher. So I want to know for how much time the sample is kept in oven. | Thank you for pointing out the need for greater methodological detail. The drying process was continued until a constant weight was achieved, which required approximately five days (120 hours) at the selected temperature. This information has now been added to the Materials and Methods section for clarity and reproducibility. |
