# Supplementary material for: Formulation and biological evaluation of a bacterial nanocellulose-based nanoemulsion hydrogel loaded with Persea americana Mill. seed extract: An anti-aging assessment
Source: PLoS One. 2026 Nov 5;21(11):e0359807. doi: 10.1371/journal.pone.0359807 (PMC13739224; doi:10.1371/journal.pone.0359807)
Supplement: Peer review history

# Peer review: Decision Letter 0

**DOI:** 10.1371/journal.pone.0359807.r001

**Authors:** Nirav Patel

16 Jun 2026

Dear Dr. Heni Rachmawati,

Thank you for submitting your manuscript to PLOS ONE. After careful consideration, we feel that it has merit but does not fully meet PLOS ONE’s publication criteria as it currently stands. Therefore, we invite you to submit a revised version of the manuscript that addresses the points raised during the review process.

Please submit your revised manuscript by Jul 31 2026 11:59PM. If you will need more time than this to complete your revisions, please reply to this message or contact the journal office at plosone@plos.org. When you're ready to submit your revision, log on to https://www.editorialmanager.com/pone/ and select the 'Submissions Needing Revision' folder to locate your manuscript file.

- A letter that responds to each point raised by the academic editor and reviewer(s). You should upload this letter as a separate file labeled 'Response to Reviewers'.
- A marked-up copy of your manuscript that highlights changes made to the original version. You should upload this as a separate file labeled 'Revised Manuscript with Track Changes'.
- An unmarked version of your revised paper without tracked changes. You should upload this as a separate file labeled 'Manuscript'.

If you would like to make changes to your financial disclosure, please include your updated statement in your cover letter. Guidelines for resubmitting your figure files are available below the reviewer comments at the end of this letter.

If applicable, we recommend that you deposit your laboratory protocols in protocols.io to enhance the reproducibility of your results. Protocols.io assigns your protocol its own identifier (DOI) so that it can be cited independently in the future. For instructions see: https://journals.plos.org/plosone/s/submission-guidelines#loc-laboratory-protocols. Additionally, PLOS ONE offers an option for publishing peer-reviewed Lab Protocol articles, which describe protocols hosted on protocols.io. Read more information on sharing protocols at https://plos.org/protocols?utm_medium=editorial-email&utm_source=authorletters&utm_campaign=protocols.

As the corresponding author, your ORCID iD is verified in the submission system and will appear in the published article. PLOS supports the use of ORCID, and we encourage all coauthors to register for an ORCID iD and use it as well. Please encourage your coauthors to verify their ORCID iD within the submission system before final acceptance, as unverified ORCID iDs will not appear in the published article. *Only*  the individual author can complete the verification step; PLOS staff *cannot*  verify ORCID iDs on behalf of authors.

We look forward to receiving your revised manuscript.

Kind regards,

Nirav Patel

Academic Editor

PLOS One

Journal requirements:

When submitting your revision, we need you to address these additional requirements.

1. Please ensure that your manuscript meets PLOS ONE's style requirements, including those for file naming. The PLOS ONE style templates can be found at

https://journals.plos.org/plosone/s/file?id=wjVg/PLOSOne_formatting_sample_main_body.pdf and

https://journals.plos.org/plosone/s/file?id=ba62/PLOSOne_formatting_sample_title_authors_affiliations.pdf

2. To comply with PLOS One submissions requirements, in your Methods section, please provide additional information regarding the experiments involving animals and ensure you have included details on methods of (1) anesthesia and/or analgesia, and (2) efforts to alleviate suffering

3. In your Methods section, please include the following details:

1. The source, age, and weight of the rats used in the animal experiments.

2. The source of the avocados used in the study.

4. Thank you for stating the following financial disclosure:

“This work was supported by Beasiswa Pendidikan Indonesia (BPI) (Grant No. 202101121555), Center for Higher Education Funding and Assessment (PPAPT) under the Ministry of Higher Education, Science, and Technology, Indonesia and the Indonesia Endowment Funds for Education (LPDP) under the Ministry of Finance, Indonesia.”

Please state what role the funders took in the study.  If the funders had no role, please state: "The funders had no role in study design, data collection and analysis, decision to publish, or preparation of the manuscript."

If this statement is not correct you must amend it as needed.

Please include this amended Role of Funder statement in your cover letter; we will change the online submission form on your behalf.

5. Thank you for stating the following in the Acknowledgments Section of your manuscript:

“This work was supported by Beasiswa Pendidikan Indonesia (BPI) (Grant No. 202101121555), Center for Higher Education Funding and Assessment (PPAPT) under the Ministry of Higher Education, Science, and Technology, Indonesia and the Indonesia Endowment Funds for Education (LPDP) under the Ministry of Finance, Indonesia.”

We note that you have provided funding information that is currently declared in your Funding Statement. However, funding information should not appear in the Acknowledgments section or other areas of your manuscript. We will only publish funding information present in the Funding Statement section of the online submission form.

Please remove any funding-related text from the manuscript and let us know how you would like to update your Funding Statement. Currently, your Funding Statement reads as follows:

“This work was supported by Beasiswa Pendidikan Indonesia (BPI) (Grant No. 202101121555), Center for Higher Education Funding and Assessment (PPAPT) under the Ministry of Higher Education, Science, and Technology, Indonesia and the Indonesia Endowment Funds for Education (LPDP) under the Ministry of Finance, Indonesia.”

Please include your amended statements within your cover letter; we will change the online submission form on your behalf.

6. PLOS requires an ORCID iD for the corresponding author in Editorial Manager on papers submitted after December 6th, 2016. Please ensure that you have an ORCID iD and that it is validated in Editorial Manager. To do this, go to ‘Update my Information’ (in the upper left-hand corner of the main menu), and click on the Fetch/Validate link next to the ORCID field. This will take you to the ORCID site and allow you to create a new iD or authenticate a pre-existing iD in Editorial Manager.

7. Please include your full ethics statement in the ‘Methods’ section of your manuscript file. In your statement, please include the full name of the IRB or ethics committee who approved or waived your study, as well as whether or not you obtained informed written or verbal consent. If consent was waived for your study, please include this information in your statement as well.

If the reviewer comments include a recommendation to cite specific previously published works, please review and evaluate these publications to determine whether they are relevant and should be cited. There is no requirement to cite these works unless the editor has indicated otherwise.

Additional Editor Comments:

The study requires substantial revision, including clarification of the scientific rationale, formulation development strategy, experimental methodology, optimization approach, and supporting data. Additional experimental work and technical justification may be necessary to adequately support the proposed formulation and conclusions.

The authors should carefully address each comment separately and provide a detailed point-by-point response. All revisions should be clearly highlighted in the revised manuscript (preferably using yellow color) to facilitate evaluation.

1. What is the advantage of combining bacterial nanocellulose with a nanoemulsion instead of using the extract or nanoemulsion alone?

2. The abstract is currently too general and lacks sufficient technical details. Please revise it according to the standard format of a research article, including the study objective, methodology, key findings with important numerical results, and the main conclusion.

3. The Introduction is descriptive and lacks sufficient technical justification. Please clearly discuss the formulation strategy, ingredient selection, CQAs, CMAs, CPPs, optimization approach, evaluation parameters, expected outcomes, research gap, novelty, and objectives of the study.

4. The statement is technically inaccurate for a conventional serum formulation, as serum products are not necessarily dependent on gelling agents. Please clarify the developed formulation properties.

5. Verify reference 10. Many other references are required to be verified again for their correctness of use as per study.

6. These properties enable BNC not only to function as a gelling agent. How? Justify technically.

7. The extraction procedure requires additional details to ensure reproducibility. Please specify the amount of avocado seed powder used, solvent-to-solid ratio, extraction volume, extraction temperature, agitation conditions (if any), extraction yield, and the final drying/storage conditions of the extract. The rationale for selecting ethanol and performing three maceration cycles should also be provided.

8. How was the nanoemulsion design selected? No pseudo-ternary phase diagram. No screening study. Please provide data/results for those.

9. Various concentrations of avocado seed extract, ranging from 0.1% to 5.0%,, on what basis? Justify.

10. CPP information are missing.

11. Optimization criteria are missing for formulation.

12. The nanoemulsion development strategy, formulation optimization approach, critical material attributes (CMAs), critical process parameters (CPPs), and critical quality attributes (CQAs) are not adequately described and should be clearly presented to ensure scientific rigor and reproducibility.

13. Verify Reference 29.

14. The BNC preparation method lacks critical fermentation, purification, and characterization details. Please provide sufficient information regarding inoculum preparation, fermentation conditions, purification procedures, yield, and characterization to ensure reproducibility of the study.

15. How much BNC was added?

16. After other raw materials--????

17. Why 75°C? Could affect nanoemulsion droplet size and stability. Could degrade phytoconstituents. Justify.

18. How does BNC convert the nanoemulsion into a serum?

19. What is the final dosage form?

Reviewers' comments:

Reviewer's Responses to Questions

**Comments to the Author**

1. Is the manuscript technically sound, and do the data support the conclusions?

Reviewer #1: Partly

**********

2. Has the statistical analysis been performed appropriately and rigorously?

Reviewer #1: Yes

**********

3. Have the authors made all data underlying the findings in their manuscript fully available?

The PLOS Data policy

Reviewer #1: No

**********

4. Is the manuscript presented in an intelligible fashion and written in standard English?

Reviewer #1: Yes

**********

Reviewer #1: Hello sir/mam

After reviewing the articles I feel that the author has missed to tell for how many time the sample had to be in oven at 50 degree. Also what will happen to the essential and volatile oils present in the Avocado after heating in oven.

**********

what does this mean?). If published, this will include your full peer review and any attached files.

If you choose “no”, your identity will remain anonymous but your review may still be made public.

**Do you want your identity to be public for this peer review?**  For information about this choice, including consent withdrawal, please see our Privacy Policy

Reviewer #1: **Yes:**  ABHISHEK RAJ JOSHI

**********

[NOTE: If reviewer comments were submitted as an attachment file, they will be attached to this email and accessible via the submission site. Please log into your account, locate the manuscript record, and check for the action link "View Attachments". If this link does not appear, there are no attachment files.]

To ensure your figures meet our technical requirements, please review our figure guidelines: https://journals.plos.org/plosone/s/figures

You may also use PLOS’s free figure tool, NAAS, to help you prepare publication quality figures: https://journals.plos.org/plosone/s/figures#loc-tools-for-figure-preparation.

NAAS will assess whether your figures meet our technical requirements by comparing each figure against our figure specifications.

Attachment Submitted filename: PONE-D-26-10500.pdf

Attachment Submitted filename: Review of Attachments for Manuscript Number PONE.docx

# Author comment: Author response to Decision Letter 1

**DOI:** 10.1371/journal.pone.0359807.r002

19 Jul 2026

Dear Editors,

We are pleased to resubmit our revised manuscript entitled:

“Formulation and Biological Evaluation of a Bacterial Nanocellulose-Based Nanoemulsion Hydrogel Loaded with Persea americana Mill. Seed Extract: An Anti-Aging Assessment”

for consideration for publication in PLOS ONE.

We sincerely thank the Editor and Reviewers for their valuable comments and constructive suggestions. We have carefully considered all remarks and have revised the manuscript accordingly. We believe that these revisions have significantly improved the scientific quality, clarity, and reproducibility of our work.

All of the response included in the file "Response to Reviewers" that we upload prior in this online submission. We appreciate the Editor’s and Reviewers’ time and effort in evaluating our work. We hope that our response and the revised manuscript is now suitable for publication in PLOS ONE, and we look forward to your favorable consideration.

Sincerely,

Prof. Dr. apt. Heni Rachmawati, M.Si.

Department of Pharmaceutics, School of Pharmacy

Institut Teknologi Bandung

Bandung, West Java, Indonesia

E-mail: h_rachmawati@itb.ac.id

Attachment Submitted filename: Response to Reviewers.docx

# Peer review: Decision Letter 1

**DOI:** 10.1371/journal.pone.0359807.r003

**Authors:** Nirav Patel

17 Sep 2026

Formulation and biological evaluation of a bacterial nanocellulose-based nanoemulsion hydrogel loaded with Persea americana Mill. seed extract: An anti-aging assessment

PONE-D-26-10500R1

Dear Dr. Rachmawati,

We’re pleased to inform you that your manuscript has been judged scientifically suitable for publication and will be formally accepted for publication once it meets all outstanding technical requirements.

Within one week, you’ll receive an e-mail detailing the required amendments. When these have been addressed, you’ll receive a formal acceptance letter and your manuscript will be scheduled for publication.

An invoice will be generated when your article is formally accepted. Please note, if your institution has a publishing partnership with PLOS and your article meets the relevant criteria, all or part of your publication costs will be covered. Please make sure your user information is up-to-date by logging into Editorial Manager at Editorial Manager® and clicking the ‘Update My Information' link at the top of the page. For questions related to billing, please contact billing support.

If your institution or institutions have a press office, please notify them about your upcoming paper to help maximize its impact. If they’ll be preparing press materials, please inform our press team as soon as possible -- no later than 48 hours after receiving the formal acceptance. Your manuscript will remain under strict press embargo until 2 pm Eastern Time on the date of publication. For more information, please contact onepress@plos.org.

Kind regards,

Nirav Patel

Academic Editor

PLOS One

Additional Editor Comments (optional):

Reviewers' comments:

Reviewer's Responses to Questions

**Comments to the Author**

2. Is the manuscript technically sound, and do the data support the conclusions?

Reviewer #2: Yes

**********

3. Has the statistical analysis been performed appropriately and rigorously?

Reviewer #2: Yes

**********

4. Have the authors made all data underlying the findings in their manuscript fully available?

The PLOS Data policy

Reviewer #2: Yes

**********

5. Is the manuscript presented in an intelligible fashion and written in standard English?

Reviewer #2: Yes

**********

Reviewer #2: The manuscript entitled “Formulation and Biological Evaluation of a Bacterial Nanocellulose-Based Nanoemulsion Hydrogel Loaded with Persea americana Mill. Seed Extract: An Anti-Aging Assessment” presents a well-designed and systematically conducted study. The manuscript is clearly drafted, well organized, and written in a manner that facilitates understanding of the objectives, experimental approach, results, and conclusions.

The formulation development and characterization studies appear to have been carefully planned and appropriately executed. The experimental methods are suitable for addressing the stated objectives, and the in-vivo evaluation provides relevant evidence regarding the potential anti-aging activity of the developed formulation. The integration of bacterial nanocellulose, a nanoemulsion-based delivery system, and Persea americana Mill. seed extract represents an interesting formulation approach.

The results are presented clearly and are adequately supported by the experimental data. The discussion is generally consistent with the findings and provides a reasonable scientific explanation for the observed results. Importantly, the conclusions are supported by the data generated in the study.

Overall, the manuscript is scientifically sound, well written, and presents a complete set of studies relevant to the proposed formulation and its biological evaluation. I did not identify any major methodological, analytical, or interpretative concerns that would affect the validity of the findings.

**Do you want your identity to be public for this peer review?**  For information about this choice, including consent withdrawal, please see our Privacy Policy

Reviewer #2: No

**********

# Editor report: Acceptance letter

**DOI:** 10.1371/journal.pone.0359807.r004

**Authors:** Nirav Patel

PONE-D-26-10500R1

PLOS One

Dear Dr. Rachmawati,

I'm pleased to inform you that your manuscript has been deemed suitable for publication in PLOS One. Congratulations! Your manuscript is now being handed over to our production team.

At this stage, our production department will prepare your paper for publication. This includes ensuring the following:

* All references, tables, and figures are properly cited

* All relevant supporting information is included in the manuscript submission,

* There are no issues that prevent the paper from being properly typeset

You will receive further instructions from the production team, including instructions on how to review your proof when it is ready. Please keep in mind that we are working through a large volume of accepted articles, so please give us a few days to review your paper and let you know the next and final steps.

Lastly, if your institution or institutions have a press office, please let them know about your upcoming paper now to help maximize its impact. If they'll be preparing press materials, please inform our press team within the next 48 hours. Your manuscript will remain under strict press embargo until 2 pm Eastern Time on the date of publication. For more information, please contact onepress@plos.org.

You will receive an invoice from PLOS for your publication fee after your manuscript has reached the completed accept phase. If you receive an email requesting payment before acceptance or for any other service, this may be a phishing scheme. Learn how to identify phishing emails and protect your accounts at https://explore.plos.org/phishing.

If we can help with anything else, please email us at customercare@plos.org.

Thank you for submitting your work to PLOS One and supporting open access.

Kind regards,

PLOS One Editorial Office Staff

on behalf of

Dr. Nirav Patel

Academic Editor

PLOS One
